# Synthesis and Fungicidal Activity of 1-(Carbamoylmethyl)-2-aryl-3,1-benzoxazines

**DOI:** 10.3390/molecules22071103

**Published:** 2017-07-03

**Authors:** Zi-Long Tang, Lian Wang, Jing-Zhao Tan, Yi-Chao Wan, Yin-Chun Jiao

**Affiliations:** 1 Key Laboratory of Theoretical Organic Chemistry and Functional Molecule of Ministry of Education, Hunan University of Science and Technology, Xiangtan 411201, China; hnustxifeng@163.com (L.W.); 18672028977@163.com (J.-Z.T.); 07wanyichao@163.com (Y.-C.W.); 2 School of Chemistry and Chemical Engineering, Hunan University of Science and Technology, Xiangtan 411201, China; 1060023@hnust.edu.cn

**Keywords:** disubstituted-3,1-benzoxazine, heterocycles, synthesis, BF_3_·OEt_2_, fungicidal activity

## Abstract

A series of new 1-(carbamoylmethyl)-2-aryl-3,1-benzoxazines were prepared in moderate to good yields by BF_3_·OEt_2_-catalyzed reactions of aromatic aldehydes with 2-(*N*-substituted carbamoylmethylamino)benzyl alcohols. The structures of the target compounds were confirmed by IR, ^1^H-NMR, ^13^C-NMR, and elemental analyses. The fungicidal activities of the target compounds against plant fungi were preliminarily evaluated, and some of them exhibited good activity.

## 1. Introduction

3,1-Benzoxazine and 3,1-benzoxazinone derivatives have received growing attention due to their broad biological activities. 3,1-Benzoxazine derivatives show anticonvulsant [1], herbicidal [2], fungicidal [3,4], and anticancer activity [5], and some are potent progesterone receptor (PR) agonists [6] or DNA-binding antitumor agents [7]. 3,1-Benzoxazinones exhibit antihypertensive [8] and antiproliferative activities [9], or are potent PR agonists/antagonists [10,11], potent human leukocyte elastase inhibitors [12], serine protease inhibitors [13,14,15], long chain fatty acid elongase 6 inhibitors [16], NK_1_/NK_3_ receptor antagonists [17], α-chymotrypsin inhibitors [18], mineralocorticoid receptors antagonists [19], and are even used as anti-HIV-1 reverse transcriptase inhibitors [20,21]. Therefore, the synthesis of 3,1-benzoxazines and 3,1-benzoxazinone has attracted considerable interest. The condensation of 2-aminobenzyl alcohol or its derivatives with aldehydes using acetic acid or *p*-toluenesulfonic acid (TsOH) as catalyst is the widely-used way to synthesize 3,1-benzoxazines [6,22,23]. Palladium-catalyzed cyclization of 2-alkynylanilides also provides a route to substituted 3,1-benzoxazines [24]. Recently, hypervalent iodine-mediated oxygenation of tertiary amines afforded a new way [25]. As for 3,1-benzoxazinones, the frequently used protocol is the reaction of 2-aminobenzyl alcohol or its derivatives with phosgene [8,26]. More recently, silver-catalyzed incorporation of carbon dioxide into 2-alkynylanilides afforded a new route [27]. In spite of the progress in their preparation, the development of more efficient ways and the synthesis of novel 3,1-benzoxazine derivatives are still highly desirable for drug discovery, as well as medicinal and pesticide chemistry. To our knowledge, 3,1-benzoxazines have received less attention compared with 3,1-benzoxazinones. Particularly, there are only few reports about the activities of benzoxazines against plant fungi [3,4]. Thus, we present herein the synthesis of novel 1-(carbamoylmethyl)-2-aryl-3,1-benzoxazines, as a continuation of our ongoing project aimed at searching for novel fungicidal active compounds, by condensation reactions of 2-(*N*-substituted carbamoylmethylamino)benzyl alcohols with aldehydes in the presence of BF_3_·OEt_2_, and also report their fungicidal activities against plant fungi.

## 2. Results and Discussion

### 2.1. Chemistry

**Scheme 1:**
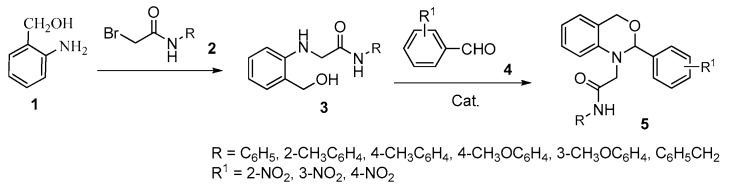
Synthesis of 1-(carbamoylmethyl)-2-aryl-3,1-benzoxazines **5**.

The synthetic route to the title compounds **5a**–**r** is shown in Scheme 1. The key intermediate 2-(*N*-substituted carbamoylmethylamino)benzyl alcohols **3** (also named as: *N*-substituted 2-(2-(hydroxymethyl)phenylamino)acetamide) were prepared by selective *N*-alkylation of 2-aminobenzyl alcohol with *N*-substituted 2-bromoacetamide **2**. The reaction exclusively occurred at the nitrogen atom giving products **3a**–**f** (^1^H-NMR and ^13^C-NMR Data in Supplementary materials) in 65–73% yields when the reaction was carried out in a mixture solvent of *N*,*N*-dimethyl formamide (DMF) and tetrahydrofuran (THF) (*v*/*v* = 1:2) with potassium carbonate as base. Then, we began to synthesize the target products **5a**–**r** (^1^H-NMR and ^13^C-NMR Data in Supplementary materials). The preparation of **5a** was selected as model to optimize the reaction conditions. Firstly, reaction of 2-(*N*-(2-methylphenyl)carbamoylmethylamino)benzyl alcohol **3a** with 3-nitrobenzaldehyde **4a** in the presence of BF_3_·OEt_2_ (10% mol) in THF under 65 °C gave the desired product **5a** in 35 yields (No. 1, Table 1). By optimizing the conditions, the yield was improved to 55% (No. 6). Under the same conditions, compounds **5b**–**r** were synthesized in 40–85% yields. As shown in Table 1, for compounds with an amide nitrogen connected to the benzyl group, a higher yield was achieved than for those with a phenyl group (No. 16–18 vs. No. 13–15, Table 1), and moreover, the reaction yields of the former depended on the position of the nitro group on the benzene ring in order of para > ortho > meta. But, the yields for those with an amide nitrogen connected with an aryl group presented the order of meta > para > ortho.

The structures of the products were established on the basis of their spectroscopic data (IR, ^1^H-NMR, ^13^C-NMR) and elemental analysis. All compounds exhibit characteristic signals appropriately (see experimental section). This can be illustrated with compound **5a**. In IR spectra, strong absorption at 1661 cm^−1^ corresponds to the stretching vibration of the C=O group; and absorption at 1586 and1536 cm^−1^ to the C=C bond. A singlet at 5.88 ppm observed in ^1^H-NMR spectra corresponds to OCHN proton of the benzoxazine ring. Particularly, the OCH_2_ protons within the benzoxazine ring absorb as two doublets at 4.74 and 4.97 ppm instead of singlet. These are characteristic signals indicating the formation of the benzoxazine ring [28]. Interestingly, the NCH_2_CO protons also absorb as two doublets at 3.88 and 4.13 ppm. A singlet at 2.06 ppm corresponds to CH_3_ protons. In ^13^C-NMR, a signal at 167.30 ppm corresponds to the C=O carbon, and signals at 88.59 and 64.74 ppm stand for the OCHN and OCH_2_ carbons, respectively. CH_3_ carbon absorbs at 17.37 ppm.

### 2.2. Fungicidal Activity Assay

According to standard method NY/T1156.5–2006 [29], the in vitro fungicidal activities of the prepared compounds **5a**–**r** were evaluated, adopting the mycelium growth rate test method. The fungicidal activity was tested against *Sclerotonia sclerotiorum*, *Botrytis cinerea*, *Rhizoctonia solani*, *Gibberella zeae* and *Phytophythora capsic* at 25 µg/mL, and against *Magnaporthe oryzae* at 50 µg/mL. The activities were expressed as inhibition rate (%), and the results are summarized in Table 2. All compounds exhibited certain activities against the tested fungi, and some showed good activities. Compounds **5d** and **5g** showed 60.1% and 54.5% activity against *M. oryzae*, respectively, which are all higher than that of chlorothalonil (53.8%). The activity of compound **5o** (51.7%) is closed to that of chlorothalonil. Compound **5i** showed 71.9% activity against *S. sclerotiorum* at 25 µg/mL, which is close to that of chlorothalonil (84.9%). In addition, the compounds with the amide nitrogen atom connected with an aryl group were more active than those with a benzyl group. For *S. sclerotiorum* and *R. solani*, when the substituent at the 4-position of the benzene ring was connected with the amide nitrogen, the compounds with a NO_2_ group at the 4-position of the benzene ring exhibited higher activities than those with a NO_2_ group at 3- or 2-position.

## 3. Experimental Section 

### 3.1. Materials and Reagents

All solvents were dried by standard procedure. Aromatic aldehydes, 2-aminobenzyl alcohol, and substituted anilines were commercially available. Infrared spectra were recorded on a Nicolet-6700 FT-IR. ^1^H- and ^13^C-NMR spectra were recorded on Bruker Avance-500 MHz spectrometer. Elemental analysis was measured on PE 2400 II CHNS instrument. Melting points were determined on a WRS-1B digital melting point instrument and uncorrected.

### 3.2. Chemical Synthesis

#### 3.2.1. Synthesis of *N*-Substituted 2-(2-(hydroxymethyl)phenylamino)acetamides **3a**–**f**

*General Procedure:* Into a 150 mL round bottom flask equipped with a condenser, *N*-(2-methylphenyl)-2-bromoacetamide (2.270 g, 10 mmol), 2-aminobenzyl alcohol (1.476 g, 12 mmol), potassium carbonate (1.932 g, 14 mmol) and mixed solvent of DMF and THF (45 mL, *v*:*v* = 1:2) were added with stirring. The mixture was heated at 65 °C for 12 h (checked by TLC). Then, the solvent was evaporated under reduced pressure. Saturated brine (50 mL) was added to the residue and extracted with ethyl acetate (3 × 50 mL). The organic phase was washed sequentially with water (2 × 50 mL), saturated brine (2 × 50 mL), and dried over Na_2_SO_4_, and filtered. The filtrate was evaporated under reduced pressure, the obtained residue purified by silica gel flash chromatography with ethyl acetate–petroleum ether (*v*/*v* = 1:2) as eluent, giving the product **3a** (73% yield) as a white solid.

*N-(2-Methylphenyl)-2-(2-(hydroxymethyl)phenylamino)acetamide* (**3a**): Yield 73%. White solid, m.p.: 115.1–118.3 °C; ^1^H-NMR (500 MHz, CDCl_3_) *δ*: 8.53 (s, 1H), 7.92 (d, *J* = 5 Hz,1H), 7.23–7.26 (m, 2H), 7.11 (d, *J* = 7.5 Hz, 2H), 7.09 (t, *J* = 7.5 Hz, 1H),6.79 (t, *J* = 7.0 Hz, 1H), 6.67 (d, *J* = 8.0 Hz, 1H), 5.66 (s, 1H), 4.76 (d, *J* = 5 Hz, 2H), 3.98 (s, 2H), 2.04 (s, 1H), 1.97 (s, 3H, -CH_3_); ^13^C-NMR (125 MHz, CDCl_3_) *δ*: 168.69, 146.20, 135.26, 131.52, 130.37, 129.91, 129.24, 126.80, 125.15, 125.00, 122.20, 118.84, 111.48, 64.75, 48.89, 17.11; IR (KBr, cm^−1^) ν: 3309, 3257, 1670, 1585, 1541, 1264, 1011, 748.

*N-(4-Methylphenyl)-2-(2-(hydroxymethyl)phenylamino)acetamide* (**3b**): Eluent: acetate/petroleum ether (*v*/*v* = 1:2); Yield 73%. White solid, m.p.: 123.4–124.5 °C; ^1^H-NMR (500 MHz, CDCl_3_) *δ*: 8.51 (s, 1H), 7.33 (d, *J* = 8.0 Hz, 2H),7.20 (t, *J* = 8.0 Hz, 1H) 7.06–7.09 (m, 3H), 6.77 (t, *J* = 6.0 Hz, 1H), 6.58 (s, 1H), 5.52 (s, 1H), 4.72 (d, *J* = 5 Hz,2H), 3.86 (s, 2H), 2.28 (s,3H, -CH_3_); ^13^C-NMR (125 MHz, CDCl_3_) *δ*: 169.60, 146.40, 134.60, 134.39, 129.79, 129.48 (2C),129.41, 125.19, 120.31 (2C), 118.59, 111.30, 64.27, 48.87, 20.90; IR (KBr, cm^−1^) ν: 3398, 3321, 3229, 1677, 1609, 1552, 1525, 1313, 992, 818, 738.

*N-(4-Methyloxyphenyl)-2-(2-(hydroxymethyl)phenylamino)acetamide* (**3c**): Eluent: acetate/petroleum ether (*v*/*v* = 1:2); Yield 68%. Pale yellow solid, m.p.: 107.0–107.2 °C; ^1^H-NMR (500 MHz, CDCl_3_) *δ*: 8.59 (s, H), 7.34 (d, *J* = 7.0Hz, 2H), 7.20 (s, 1H), 7.08 (s, 1H), 6.76–6.80 (m, 3H), 6.56 (t, *J* = 5.0 Hz, 1H), 5.51 (s, 1H), 4.69 (s, 2H), 3.83 (s, 2H), 3.74 (s, 3H, -OCH_3_); ^13^C-NMR (125 MHz, CDCl_3_) *δ*: 169.38, 156.62, 146.31, 130.17, 129.68, 129.30, 125.06, 121.97, 118.46, 114.00 (2C), 111.16, 64.21, 55.36, 48.67; IR (KBr, cm^−1^) ν: 3399, 3298, 1669, 1609, 1507, 1453, 1300, 1236, 1000, 825, 743.

*N-(3-Methyloxyphenyl)-2-(2-(hydroxymethyl)phenylamino)acetamido* (**3d**): Eluent: acetate/petroleum ether (*v*/*v* = 1:2); Yield 65%. Pale yellow solid, m.p.: 120.9–124.4 °C; ^1^H-NMR (500 MHz, CDCl_3_) *δ*: 8.48 (s, 1H), 7.08–7.18 (m, 3H), 7.02 (d, *J* = 7.0 Hz, 1H), 6.87 (d, *J* = 8.0 Hz, 1H), 6.71 (t, *J* = 7.0 Hz, 1H), 6.56–6.58 (m, 1H), 6.52 (d, *J* = 8.0 Hz, 1H), 4.68 (s, 2H), 3.83 (s, 2H), 3.69 (s, 3H, -OCH_3_); ^13^C-NMR (125 MHz, CDCl_3_) *δ*: 169.36, 160.11, 146.44, 138.42, 129.96, 129.68, 129.36, 125.12, 118.83, 112.22, 111.51, 110.32, 105.85, 64.62, 55.34, 49.17; IR (KBr, cm^−1^) ν: 3391, 3336, 1672, 1601, 1560, 1516, 1455, 1430, 1050, 1006, 780, 749.

*N-Phenyl-2-(2-(hydroxymethyl)phenylamino)acetamido* (**3e**): Eluent: acetate/petroleum ether (*v*/*v* = 1:2); Yield 70%. White solid, m.p.: 117.2–119.3 °C; ^1^H-NMR (500 MHz, CDCl_3_) *δ*: 8.59 (s, 1H), 7.45 (d, *J* = 8.0 Hz, 2H), 7.26 (t, *J* = 7.5 Hz, 2H), 7.19 (td, *J* = 8.0 Hz, 1.0 Hz, 1H), 7.08 (t, *J* = 6.5 Hz, 2H), 6.77 (t, *J* = 7.5 Hz, 1H), 6.57(d, *J* = 8.0 Hz, 1H), 5.52 (br, 1H), 4.72 (s, 2H), 3.86 (s, 2H), 2.77 (br, 1H); ^13^C-NMR (125 MHz, CDCl_3_) *δ*: 169.61, 146.39, 137.18, 129.87, 129.41, 129.00 (2C), 125.18, 124.70, 120.20 (2C), 118.72, 111.37, 64.41, 48.98; IR (KBr, cm^−1^) ν: 3342, 3265, 1678, 1608, 1563, 1513, 1498, 1444, 1313, 1254, 1003, 751.

*N-Benzyl-2-(2-(hydroxymethyl)phenylamino)acetamido* (**3f**): Eluent: acetate/petroleum ether (*v*/*v* = 1:2); Yield 66%. Pale yellow solid, m.p.: 107.7–108.8 °C; ^1^H-NMR (500 MHz, CDCl_3_) *δ*: 7.13–7.25 (m, 8H), 7.02 (d, *J* = 6.0 Hz, 1H), 6.73 (s, 1H, NH), 6.51 (s, 1H, NH), 4.60 (s, 2H), 4.37 (s, 2H), 3.77 (s, 2H); ^13^C-NMR (125 MHz, CDCl_3_) *δ*: 171.14, 146.48, 138.01, 129.63, 129.28, 128.62 (2C), 127.45 (2C), 127.41, 125.07, 118.27, 111.11, 64.35, 48.19, 43.01; IR (KBr, cm^−1^) ν: 3375, 3254, 1645, 1585, 1530, 1505, 1450, 1427, 1365, 1311, 1252, 1016, 751.

#### 3.2.2. Synthesis of 2,4-Dihydro-1*H*-3,1-benzoxazines **5a–r**

*General Procedure:* Under nitrogen, into a 100 mL three-necked round bottom flask equipped with a condenser, *N*-(2-methylphenyl)-2-(2-(hydroxymethyl)phenylamino)acetamide **3a** (0.405 g, 1.5 mmol), 3-nitrobenzaldehyde (0.339 g, 2.25 mmol), THF (30 mL), BF_3_·OEt_2_ (0.031 g, 0.3 mmol) and molecular sieve 4Å (0.250 g) were added with stirring. The solution was heated at 65 °C for 10 h (checked by TLC). Then, the solvent was evaporated under reduced pressure. Ethyl acetate (70 mL) was added to the residue, and the obtained solution washed sequentially with water (2 × 40 mL) and saturated brine (2 × 40 mL). The organic phase was dried over Na_2_SO_4_ and filtered. The filtrate was evaporated under reduced pressure and the obtained residue purified by silica gel flash chromatography with ethyl acetate–petroleum ether (*v*/*v* = 1:5) as eluent, giving the product **5a** (55% yield) as a yellow solid.

*1-((2-Methylphenyl)carbamoylmethyl)-2-(3-nitrophenyl)-2,4-dihydro-1H-3,1-benzoxazine* (**5a**): Yield 55%. Yellow solid; m.p.: 151.2–152.2 °C; ^1^H-NMR (500 MHz, CDCl_3_) *δ*: 8.50 (s, 1H), 8.34 (s, 1H), 8.23 (d, *J* = 9.0 Hz, 1H), 7.84 (d, *J* = 8.0 Hz, 1H), 7.78 (d, *J* = 7.5 Hz, 1H), 7.58 (t, *J* = 8.0 Hz, 1H), 7.24 (d, *J* = 7.0 Hz, 1H), 7.19 (t, *J* = 8.0 Hz, 1H), 7.13 (t, *J* = 7.0 Hz, 1H), 7.05 (t, *J* = 7.5 Hz, 1H), 6.93–6.97 (m, 3H), 5.88 (s, 1H, NCHO), 4.97 (d, *J* = 15 Hz, 1H), 4.74 (d, *J* = 15 Hz, 1H), 4.13 (d, *J* = 18 Hz, 1H), 3.88 (d, *J* = 18 Hz, 1H), 2.06 (s, 3H); ^13^C-NMR (125 MHz, CDCl_3_) *δ*: 167.30 (C=O), 148.67, 141.92, 139.47, 135.04, 133.57, 130.53, 130.08, 128.70, 128.54, 126.86, 125.32, 125.09, 124.28, 122.82, 122.47, 122.23, 121.18, 115.20, 88.59, 64.74, 54.86, 17.37; IR (KBr, cm^−1^) ν: 3268, 1661 (C=O), 1586, 1536, 1497, 1458, 1397, 1346, 1259, 1208, 1070, 757, 733; Anal. Calcd. for C_23_H_21_N_3_O_4_: C, 68.47; H, 5.25; N, 10.42; Found: C, 68.16; H, 5.22; N, 10.37.

*1-((2-Methylphenyl)carbamoylmethyl)-2-(2-nitrophenyl)-2,4-dihydro-1H-3,1-benzoxazine* (**5b**): Eluent: acetate/petroleum ether (*v*/*v* = 1:5); Yield 45%. Yellow solid, m.p.: 162.6–162.9 °C; ^1^H-NMR (500 MHz, CDCl_3_) *δ*: 8.51(s, 1H), 7.88 (d, *J* = 7.5 Hz, 1H), 7.78 (d, *J* = 8.0 Hz, 1H), 7.55–7.59 (m, 3H), 7.16–7.24 (m, 2H), 7.09 (d, *J* = 7.0 Hz, 1H), 7.03 (t, *J* = 7.0 Hz,1H), 6.89–6.91 (m, 3H), 6.44 (s, 1H, NCHO), 4.91 (d, *J* = 15 Hz, 1H), 4.61 (d, *J* = 15 Hz, 1H), 4.13 (d, *J* = 18 Hz, 1H) 3.96 (d, *J* = 18 Hz, 1H), 1.99 (s, 3H); ^13^C-NMR (125 MHz, CDCl_3_) *δ*: 167.39 (C=O), 149.06, 141.80, 135.07, 133.07, 131.15, 130.50, 130.40, 129.34, 128.75, 128.58, 126.76, 125.24, 125.07, 125.05, 122.34, 121.99, 120.76, 114.34, 85.18, 65.30, 54.26, 17.29; IR (KBr, cm^−1^) ν: 3396, 2846, 1677 (C=O), 1606, 1533, 1515, 1499, 1466, 1459, 1354, 1326, 1186, 959, 759; Anal. Calcd. for C_23_H_21_N_3_O_4_: C, 68.47; H, 5.25; N, 10.42; Found: C, 68.75; H, 5.23; N, 10.38.

*1-((2-Methylphenyl)carbamoylmethyl)-2-(4-nitrophenyl)-2,4-dihydro-1H-3,1-benzoxazine* (**5c**): Eluent: acetate/petroleum ether (*v*/*v* = 1:5); Yield 49%. Yellow solid, m.p.: 153.4–153.8 °C; ^1^H-NMR (500 MHz, CDCl_3_) *δ*: 8.47 (s, 1H), 8.24 (d, *J* = 8.5 Hz, 2H), 7.84 (d, *J* = 8.0 Hz, 1H), 7.62 (d, *J* = 8.5 Hz, 2H), 7.18–7.25 (m, 2H), 7.13 (d, *J* = 7.5 Hz, 1H), 7.06 (t, *J* = 7.5 Hz, 1H), 6.93–6.96 (m, 3H), 5.89 (s, 1H, NCHO), 4.94 (d, *J* = 15 Hz, 1H), 4.69 (d, *J* = 15 Hz, 1H), 4.14 (d, *J* = 18 Hz, 1H), 3.88 (d, *J* = 18 Hz, 1H), 2.03 (s, 3H); ^13^C-NMR (125 MHz, CDCl_3_) *δ*: 167.28 (C=O), 148.42, 144.07, 141.68, 135.03, 130.54, 128.68 (3C), 128.50, 126.90, 125.36, 125.12, 124.15 (2C), 122.36, 122.29, 121.06, 114.90, 88.52, 64.46, 54.78, 17.39; IR (KBr, cm^−1^) ν: 3308, 1663 (C=O), 1608, 1585, 1531, 1502, 1459, 1354, 1291, 1257, 1080, 859, 741; Anal. Calcd. for C_23_H_21_N_3_O_4_: C, 68.47; H, 5.25; N, 10.42; Found: C, 68.15; H, 5.27; N, 10.46.

*1-((4-Methylphenyl)carbamoylmethyl)-2-(3-nitrophenyl)-2,4-dihydro-1H-3,1-benzoxazine* (**5d**): Eluent: acetate/petroleum ether (*v*/*v* = 1:5); Yield 56%. Yellow solid, m.p.: 168.5–169.1 °C; ^1^H-NMR (500 MHz, CDCl_3_) *δ*: 8.49 (s, 1H), 8.31 (s, 1H), 8.13 (d, *J* = 8.0 Hz, 1H), 7.72 (d, *J* = 8.0 Hz, 1H), 7.49 (t, *J* = 8.0 Hz, 1H), 7.25 (d, *J* = 8.0 Hz, 2H), 7.16–7.19 (m, 1H), 7.03 (d, *J* = 8.0 Hz, 2H), 6.88 (d, *J* = 6.5 Hz, 2H), 6.85 (d, *J* = 6.5 Hz, 1H), 5.76 (s, 1H, NCHO), 4.96 (d, *J* = 15Hz, 1H), 4.72 (d, *J* = 15 Hz, 1H), 3.92 (d, *J* = 18 Hz, 1H), 3.78 (d, *J* = 18 Hz, 1H), 2.22 (s, 3H); ^13^C-NMR (125 MHz, CDCl_3_) *δ*: 167.36 (C=O), 148.64, 142.61, 139.42, 134.48, 134.44, 133.57, 130.05, 129.57 (2C), 128.65, 124.99, 124.31, 122.80 (2C), 121.41, 119.84 (2C), 115.95, 88.81, 65.27, 55.33, 20.89; IR (KBr, cm^−1^) ν: 3318, 1681 (C=O), 1602, 1528, 1505, 1349, 1309, 1257, 1241, 1056, 964, 814, 739; Anal. Calcd. for C_23_H_21_N_3_O_4_: C, 68.47; H, 5.25; N, 10.42; Found: C, 68.13; H, 5.27; N, 10.48.

*1-((4-Methylphenyl)carbamoylmethyl)-2-(2-nitrophenyl)-2,4-dihydro-1H-3,1-benzoxazine* (**5e**): Eluent: acetate/petroleum ether (*v*/*v* = 1:5); Yield 40%. Yellow solid, m.p.: 136.0–138.5 °C; ^1^H-NMR (500 MHz, CDCl_3_) *δ*: 8.65 (s, 1H), 7.81 (d, *J* = 8.0 Hz, 1H), 7.64 (d, *J* = 8.0 Hz, 1H), 7.54 (t, *J* = 7.5 Hz, 1H), 7.45 (t, *J* = 7.5 Hz, 1H), 7.27 (d, *J* = 8.5 Hz, 2H), 7.15–7.19 (m, 1H), 7.01 (d, *J* = 7.0 Hz, 2H), 6.88 (d, *J* = 3.5 Hz, 2H), 6.83 (d, *J* = 8.5 Hz, 1H), 6.28 (s, 1H, NCHO), 4.94 (d, *J* = 15 Hz, 1H), 4.67 (d, *J* = 15 Hz, 1H), 3.92 (d, *J* = 18 Hz, 1H), 3.86 (d, *J* = 18 Hz, 1H), 2.22 (s, 3H); ^13^C-NMR (125 MHz, CDCl_3_) *δ*: 167.67 (C=O), 148.88, 143.17, 134.66, 134.24, 133.18, 131.02, 130.40, 129.55, 129.49 (2C), 128.47, 124.99, 124.94, 122.92, 121.42, 119.75 (2C), 116.10, 85.39, 66.14, 55.03, 20.90; IR (KBr, cm^−1^) ν: 3337, 2919, 1671 (C=O), 1587, 1524, 1488, 1455, 1352, 1206, 1029, 923, 738; Anal. Calcd. for C_23_H_21_N_3_O_4_: C, 68.47; H, 5.25; N, 10.42; Found: C, 68.90; H, 5.22; N, 10.36.

*1-((4-Methylphenyl)carbamoylmethyl)-2-(4-nitrophenyl)-2,4-dihydro-1H-3,1-benzoxazine* (**5f**): Eluent: acetate/petroleum ether (*v*/*v* = 1:5); Yield 48%. Yellow solid, m.p.: 157.2–158.5 °C; ^1^H-NMR (500 MHz, CDCl_3_) *δ*: 8.53 (s, 1H), 8.22 (d, *J* = 8.5 Hz, 2H), 7.64 (d, *J* = 8.5 Hz, 2H), 7.31 (d, *J* = 8.5 Hz, 2H), 7.21–7.25 (m, 1H), 7.10 (d, *J* = 8.0 Hz, 2H), 6.93–6.95 (m, 3H), 5.85 (s, 1H, NCHO), 4.99 (d, *J* = 15 Hz, 1H), 4.75 (d, *J* = 15 Hz, 1H), 4.03 (d, *J* = 18Hz, 1H), 3.85 (d, *J* = 18 Hz, 1H), 2.30 (s, 3H); ^13^C-NMR (125 MHz, CDCl_3_) *δ*: 167.38 (C=O), 148.38, 144.01, 142.34, 134.52, 134.49, 129.59 (2C), 128.66 (2C), 128.63, 125.04, 124.13 (2C), 122.78, 121.34, 119.83 (2C), 115.77, 88.68, 64.88, 55.34, 20.89; IR (KBr, cm^−^^1^) ν: 3369, 2973, 1672 (C=O), 1605, 1519, 1348, 1329, 1191, 1067, 966, 813, 745; Anal. Calcd. for C_23_H_21_N_3_O_4_: C, 68.47; H, 5.25; N, 10.42; Found: C, 68.08; H, 5.22; N, 10.47.

*1-((4-Methyloxyphenyl)carbamoylmethyl)-2-(3-nitrophenyl)-2,4-dihydro-1H-3,1-benzoxazine* (**5g**): Eluent: acetate/petroleum ether (*v*/*v* = 1:5); Yield 85%. Yellow solid, m.p.: 167.0–167.2 °C; ^1^H-NMR (500 MHz, CDCl_3_) *δ*: 8.49 (s, 1H), 8.31 (s, 1H), 8.13 (d, *J* = 8.0 Hz, 1H), 7.72 (d, *J* = 7.5 Hz, 1H), 7.49 (t, *J* = 8.0 Hz, 1H), 7.25 (d, *J* = 7.5 Hz, 2H), 7.18 (t, *J* = 6.5 Hz, 1H), 7.02 (d, *J* = 8.0 Hz, 2H), 6.84–6.88 (m, 3H), 5.76 (s, 1H, NCHO), 4.96 (d, *J* = 15Hz, 1H), 4.71 (d, *J* = 15 Hz, 1H), 3.91 (d, *J* = 18 Hz, 1H), 3.77 (d, *J* = 18 Hz, 1H), 2.22 (s, 3H); ^13^C-NMR (125 MHz, CDCl_3_) *δ*: 167.36 (C=O), 148.63, 142.62, 139.42, 134.47, 134.45, 133.59, 130.06, 129.57 (2C), 128.65, 124.99, 124.31, 122.80 (2C), 121.40, 119.84 (2C), 115.95, 88.81, 65.29, 55.32, 20.91; IR (KBr, cm^−1^) ν: 3319, 1682 (C=O), 1603, 1527, 1504, 1444, 1404, 1350, 1309, 1257, 1242, 1179, 1057, 965, 931, 814, 740; Anal. Calcd. for C_23_H_21_N_3_O_5_: C, 65.86; H, 5.05; N, 10.02; Found: C, 65.56; H, 5.03; N, 10.06.

*1-((4-Methyloxyphenyl)carbamoylmethyl)-2-(2-nitrophenyl)-2,4-dihydro-1H-3,1-benzoxazine (**5h**)*: Eluent: acetate/petroleum ether (*v*/*v* = 1:5); Yield 66%. Yellow solid, m.p.: 156.1–158.2 °C; ^1^H-NMR (500 MHz, CDCl_3_) *δ*: 8.67 (s, 1H), 7.89 (d, *J* = 8.0 Hz, 1H), 7.70 (d, *J* = 7.5 Hz, 1H), 7.62 (t, *J* = 7.5 Hz, 1H), 7.53 (t, *J* = 7.5 Hz, 1H), 7.36 (d, *J* = 9.0 Hz, 2H), 7.22–7.23 (m, 1H), 6.96 (d, *J* = 4.5 Hz, 2H), 6.92 (d, *J* = 8.5 Hz, 1H), 6.83 (d, *J* = 9.0 Hz, 2H), 6.36 (s, 1H, NCHO), 5.01 (d, *J* = 15 Hz, 1H), 4.74 (d, *J* = 15 Hz, 1H), 3.99 (d, *J* = 15 Hz, 1H), 3.93 (d, *J* = 15 Hz, 1H), 3.77 (s, 3H); ^13^C-NMR (125 MHz, CDCl_3_) *δ*: 167.53 (C=O), 156.67, 148.88, 143.15, 133.17, 131.03, 130.40, 130.37, 129.56, 128.46, 124.99, 124.95, 122.89, 121.47 (2C), 121.39, 116.04, 114.14 (2C), 85.38, 66.11, 55.49, 54.93; IR (KBr, cm^−1^) ν: 3339, 2942, 1674 (C=O), 1605, 1531, 1506, 1465, 1405, 1346, 1316, 1246, 1175, 1038, 962, 839, 744; Anal. Calcd. for C_23_H_21_N_3_O_5_: C, 65.86; H, 5.05; N, 10.02; Found: C, 65.46; H, 5.08; N, 9.97.

*1-((4-Methyloxyphenyl)carbamoylmethyl)-2-(4-nitrophenyl)-2,4-dihydro-1H-3,1-benzoxazine (**5i**)*: Eluent: acetate/petroleum ether (*v*/*v* = 1:5); Yield 80%. Pale yellow solid, m.p.: 156.6–158.5 °C; ^1^H-NMR (500 MHz, CDCl_3_) *δ*: 8.42 (s, 1H), 8.16 (d, *J* = 8.5 Hz, 2H), 7.57 (d, *J* = 8.5 Hz, 2H), 7.26 (d, *J* = 9.0 Hz, 2H), 7.15–7.19 (m, 1H), 6.85–6.88 (m, 3H), 6.76 (d, *J* = 9.0 Hz, 2H), 5.78 (s, 1H, NCHO), 4.92 (d, *J* = 15 Hz, 1H), 4.66 (d, *J* = 15 Hz, 1H), 3.96 (d, *J* = 18 Hz, 1H), 3.78 (d, *J* = 18 Hz, 1H), 3.71 (s, 3H); ^13^C-NMR (125 MHz, CDCl_3_) *δ*: 167.22 (C=O), 156.75, 148.39, 144.00, 142.30, 130.61, 130.13, 128.65 (2C), 125.04, 124.14 (2C), 122.72, 121.58 (2C), 121.31, 115.69, 114.25 (2C), 88.67, 64.84, 55.51, 55.24; IR (KBr, cm^−1^) ν: 3379, 2934, 1684 (C=O), 1601, 1523, 1494, 1347, 1307, 1264, 1240, 1039, 865, 763; Anal. Calcd. for C_23_H_21_N_3_O_5_: C, 65.86; H, 5.05; N, 10.02; Found: C, 66.24; H, 5.02; N, 10.07.

*1-((3-Methyloxyphenyl)carbamoylmethyl)-2-(3-nitrophenyl)-2,4-dihydro-1H-3,1-benzoxazine* (**5j**): Eluent: acetate/petroleum ether (*v*/*v* = 1:5); Yield 74%. Pale yellow solid, m.p.: 56.6–58.4 °C; ^1^H-NMR (500 MHz, CDCl_3_) *δ*: 8.65 (s, 1H), 8.39 (s, 1H), 8.21 (d, *J* = 8.0 Hz, 1H), 7.80 (d, *J* = 7.5 Hz, 1H), 7.58 (t, *J* = 7.5 Hz, 1H), 7.18–7.26 (m, 3H), 6.92–6.97 (m, 4H), 6.66 (d, *J* = 8.0 Hz, 1H), 5.84 (s, 1H, NCHO), 5.04 (d, *J* = 15 Hz, 1H), 4.80 (d, *J* = 15 Hz, 1H) ,4.00 (d, *J* = 18 Hz, 1H), 3.85 (d, *J* = 18 Hz, 1H), 3.79 (s, 3H); ^13^C-NMR (125 MHz, CDCl_3_) *δ*: 167.60 (C=O), 160.17, 148.62, 142.57, 139.36, 138.21, 133.62, 130.13, 129.83, 128.70, 125.05, 124.37, 122.87, 122.79, 121.55, 116.09, 111.87, 110.61, 105.41, 88.81, 65.29, 55.50, 55.39; IR (KBr, cm^−1^) ν: 3327, 3078, 2937, 2837, 1674 (C=O), 1606, 1530, 1494, 1458, 1349, 1290, 1220, 1155, 1085, 1046, 960, 754; Anal. Calcd. for C_23_H_21_N_3_O_5_: C, 65.86; H, 5.05; N, 10.02; Found: C, 65.52; H, 5.07; N, 9.98.

*1-((3-Methyloxyphenyl)carbamoylmethyl)-2-(2-nitrophenyl)-2,4-dihydro-1H-3,1-benzoxazine* (**5k**): Eluent: acetate/petroleum ether (*v*/*v* = 1:5); Yield 41%. Brown yellow solid, m.p.: 50.9–52.4 °C; ^1^H-NMR (500 MHz, CDCl_3_) *δ*: 8.73 (s, 1H), 7.80 (d, *J* = 8.0 Hz, 1H), 7.64 (d, *J* = 8.0 Hz, 1H), 7.54 (t, *J* = 7.5 Hz, 1H), 7.44 (t, *J* = 8.0 Hz, 1H), 7.18 (s, 1H), 7.12–7.15 (m, 2H), 7.09 (d, *J* = 8.0 Hz, 1H), 6.86–6.88 (m, 2H), 6.83 (d, *J* = 8.0 Hz, 1H), 6.57 (d, *J* = 8.0 Hz, 1H), 6.27 (s, 1H, NCHO), 4.94 ( d, *J* = 15 Hz, 1H), 4.67 (d, *J* = 15 Hz, 1H), 3.87 (d, *J* = 5 Hz, 2H), 3.70 (s, 3H); ^13^C-NMR (125 MHz, CDCl_3_) *δ*: 168.00 (C=O), 160.12, 148.84, 143.23, 138.40, 133.26, 130.98, 130.46, 129.73, 129.60, 128.50, 125.03, 124.99, 123.06, 121.59, 116.31, 111.88, 110.44, 105.38, 85.44, 66.21, 55.36, 55.19; IR (KBr, cm^−1^) ν: 3361, 2974, 2895, 1680 (C=O), 1607, 1532, 1494, 1457, 1377, 1088, 1049, 881, 753; Anal. Calcd. for C_23_H_21_N_3_O_5_: C, 65.86; H, 5.05; N, 10.02; Found: C, 65.55; H, 5.03; N, 10.06.

*1-((3-methyloxyphenyl)carbamoylmethyl)-2-(4-nitrophenyl)-2,4-dihydro-1H-3,1-benzoxazine* (**5l**): Eluent: acetate/petroleum ether (*v*/*v* = 1:5); Yield 60%. Yellow solid, m.p.: 128.7–129.9 °C; ^1^H-NMR (500 MHz, CDCl_3_) *δ*: 8.54 (s, 1H), 8.14 (d, *J* = 8.5 Hz, 2H),7.56(d,*J* =8.5 Hz, 2H), 7.13–7.18 (m, 2H), 7.10 (d, *J* = 8.0 Hz, 1H), 6.81–6.87 (m, 4H), 6.58 (td, *J* = 8.0, 2.0 Hz, 1H), 5.78 (s, 1H, NCHO), 4.92 (d, *J* = 15 Hz, 1H), 4.67 (d, *J* = 15 Hz, 1H), 3.96 (d, *J* = 18 Hz, 1H), 3.78 (d, *J* = 18 Hz, 1H) ,3.70 (s, 3H); ^13^C-NMR (125 MHz, CDCl_3_) *δ*: 167.63 (C=O), 160.20, 148.35, 143.99, 142.30, 138.30, 129.80, 128.67 (2C), 128.62, 125.07, 124.13 (2C), 122.85, 121.40, 115.86, 111.85, 110.39, 105.64, 88.65, 64.88, 55.44, 55.35; IR (KBr, cm^−1^) ν: 3366, 1666 (C=O), 1602, 1593, 1520, 1456, 1434, 1348, 1330, 1270, 1157, 1073, 1052, 972, 855, 776; Anal. Calcd. for C_23_H_21_N_3_O_5_: C, 65.86; H, 5.05; N, 10.02; Found: C, 66.15; H, 5.03; N, 9.99.

*2-(3-Nitrophenyl)-1-(phenylcarbamoylmethyl)-2,4-dihydro-1H-3,1-benzoxazine* (**5m**): Eluent: acetate/petroleum ether (*v*/*v* = 1:5); Yield 52%. Yellow solid, m.p.: 130.6–131.5 °C; ^1^H-NMR (500 MHz, CDCl_3_) *δ*: 8.59 (s, 1H), 8.35 (s, 1H), 8.18 (d, *J* = 8.0 Hz, 1H), 7.76 (d, *J* = 8.0 Hz, 1H), 7.53 (t, *J* = 8.0 Hz, 2H), 7.42 (d, *J* = 8.0 Hz, 2H), 7.27 (t, *J* = 7.5 Hz, 2H), 7.19–7.23 (m, 1H), 7.08 (t, *J* = 7.5 Hz, 1H), 6.90–6.94 (m, 2H), 5.81 (s, 1H, NCHO), 5.01 (d, *J* = 15 Hz, 1H), 4.76 (d, *J* = 15 Hz, 1H), 3.99 (d, *J* = 18 Hz, 1H), 3.84 (d, *J* = 15 Hz, 1H); ^13^C-NMR (125 MHz, CDCl_3_) *δ*: 167.61 (C=O), 148.68, 142.59, 139.42, 137.05, 133.61, 130.11, 129.14 (2C), 128.71, 125.06, 124.83, 124.35, 122.89, 122.83, 121.52, 119.83 (2C), 116.05, 88.84, 65.27, 55.44; IR (KBr, cm^−1^) ν: 3302, 3069, 1669 (C=O), 1602, 1532, 1496, 1444, 1349, 1301, 1256, 1174, 1079, 751; Anal. Calcd. for C_22_H_19_N_3_O_4_: C, 67.86; H, 4.92; N, 10.79; Found: C, 67.59; H, 4.90; N, 10.75.

*2-(2-Nitrophenyl)-1-(phenylcarbamoylmethyl)-2,4-dihydro-1H-3,1-benzoxazine* (**5n**): Eluent: acetate/petroleum ether (*v*/*v* = 1:5); Yield 44%. Yellow solid, m.p.: 157.5–158.8 °C; ^1^H-NMR (500 MHz, CDCl_3_), *δ*: 8.73 (s, 1H), 7.80 (d, *J* = 8.5 Hz, 1H), 7.64 (d, *J* = 8.0 Hz, 1H), 7.53 (t, *J* = 7.5 Hz, 1H), 7.44 (t, *J* = 7.5 Hz, 1H), 7.39 (d, *J* = 8.0 Hz, 2H), 7.18–7.23 (m, 2H), 7.12–7.14 (m, 1H), 7.02 (t, *J* = 7.5 Hz, 1H), 6.86 (d, *J* = 4.5 Hz, 2H), 6.84 (d, *J* = 8.0 Hz, 1H), 6.28 (s, 1H, NCHO), 4.95 (d, *J* = 15 Hz, 1H), 4.67 (d, *J* = 15 Hz, 1H), 3.93 (d, *J* = 18 Hz, 1H), 3.88 (d, *J* = 18 Hz, 1H); ^13^C-NMR (125 MHz, CDCl_3_) *δ*: 167.95 (C=O), 148.91, 143.17, 137.25, 133.23, 131.01, 130.46, 129.61, 129.05 (2C), 128.52, 125.02 (2C), 124.63, 123.00, 121.53, 119.76 (2C), 116.18, 85.46, 66.16, 55.13; IR (KBr, cm^−1^) ν: 3316, 3207, 1676 (C=O), 1603, 1553, 1528, 1495, 1441, 1346, 1311, 1252, 1197, 1075, 750; Anal. Calcd. for C_22_H_19_N_3_O_4_: C, 67.86; H, 4.92; N, 10.79; Found: C, 67.54; H, 4.90; N, 10.74.

*2-(4-Nitrophenyl)-1-(phenylcarbamoylmethyl)-2,4-dihydro-1H-3,1-benzoxazine* (**5o**): Eluent: acetate/petroleum ether (*v*/*v* = 1:5); Yield 46%. Yellow solid, m.p.: 142.6–144.0 °C; ^1^H-NMR (500 MHz, CDCl_3_) *δ*: 8.66 (s, 1H), 8.22 (d, *J* = 8.5 Hz, 2H), 7.64 (d, *J* = 8.5 Hz, 2H), 7.44 (d, *J* = 7.5 Hz, 2H), 7.30 (t, *J* = 8.0 Hz, 2H), 7.21–7.25 (m, 1H), 7.12 (t, *J* = 7.5 Hz, 1H), 6.92–6.95 (m, 3H), 5.86 (s, 1H, NCHO), 5.01 (d, *J* = 15 Hz, 1H), 4.75 (d, *J* = 15 Hz, 1H), 4.05 (d, *J* = 18 Hz, 1H), 3.86 (d, *J* = 18Hz, 1H); ^13^C-NMR (125 MHz, CDCl_3_) *δ*: 167.56 (C=O), 148.46, 143.99, 142.29, 137.10, 129.19 (2C), 128.73, 128.70 (2C), 125.13, 124.88, 124.23 (2C), 122.87, 121.51, 119.79 (2C), 115.91, 88.75, 64.90, 55.52; IR (KBr, cm^−1^) ν: 3365, 1666 (C=O), 1600, 1519, 1444, 1350, 1331, 1263, 853, 762; Anal. Calcd. for C_22_H_19_N_3_O_4_: C, 67.86; H, 4.92; N, 10.79; Found: C, 67.55; H, 4.94; N, 10.83.

*1-(Benzylcarbamoylmethyl)-2-(3-nitrophenyl)-2,4-dihydro-1H-3,1-benzoxazine* (**5p**): Eluent: acetate/petroleum ether (*v*/*v* = 1:5); Yield 56%. Yellow solid, m.p.: 103.3–105.7 °C; ^1^H-NMR (500 MHz, CDCl_3_) *δ*: 8.28 (s, 1H), 8.20 (d, *J* = 8.0 Hz, 1H), 7.68 (d, *J* = 7.5 Hz, 1H), 7.48 (t, *J* = 8.0 Hz, 1H), 7.22–7.26 (m, 4H), 7.15 (s, 1H), 7.07–7.09 (m, 2H), 6.92–6.93 (m, 2H), 6.85 (d, *J* = 8.0 Hz, 1H), 5.75 (s, 1H, NCHO), 4.91 (d, *J* = 15 Hz, 1H), 4.69 (d, *J* = 15 Hz, 1H), 4.49 (dd, *J* = 14.5, 6.0 Hz, 1H), 4.32 (dd, *J* = 14.5, 5.0 Hz, 1H), 3.95 (d, *J* = 18 Hz, 1H), 3.78 (d, *J* = 18 Hz, 1H); ^13^C-NMR (125 MHz, CDCl_3_) *δ*: 169.25 (C=O), 148.56, 142.52, 139.52, 137.69, 133.65, 130.00, 128.75 (2C), 128.53, 127.62, 127.56 (2C), 124.96, 124.26, 122.85, 122.47, 120.96, 115.43, 88.80, 65.24, 54.46, 43.37; IR (KBr,cm^-1^) ν: 3329, 1650 (C=O), 1530, 1494, 1459, 1426, 1353, 1328, 1248, 1084, 744; Anal. Calcd. for C_23_H_21_N_3_O_4_: C, 68.47; H, 5.25; N, 10.42; Found: C, 68.09; H, 5.22; N, 10.37.

*1-(Benzylcarbamoylmethyl)-2-(2-nitrophenyl)-2,4-dihydro-1H-3,1-benzoxazine* (**5q**): Eluent: acetate/petroleum ether (*v*/*v* = 1:5); Yield 58%. Yellow solid, m.p.: 96.2–97.3 °C; ^1^H-NMR (500 MHz, CDCl_3_) *δ*: 7.82 (s, 1H), 7.47–7.53 (m, 3H), 7.21–7.25 (m, 4H), 7.05 (d, *J* = 4.5 Hz, 2H), 6.88–6.91 (m, 2H), 6.84 (d, *J* = 8.0 Hz, 1H), 6.32 (s, 1H, NCHO), 4.84 (d, *J* = 15.0 Hz, 1H), 4.57 (d, *J* = 15.0 Hz, 1H), 4.52 (dd, *J* = 15.0, 7.0 Hz, 1H), 4.27 (dd, *J* = 15.0, 5.0 Hz, 1H), 4.03 (d, *J* = 18.0 Hz, 1H), 3.89 (d, *J* = 18.0 Hz, 1H); ^13^C-NMR (125 MHz, CDCl_3_) *δ*: 169.42 (C=O), 148.95, 142.46, 137.84, 133.02, 131.15, 130.24, 129.46, 128.64 (2C), 128.39, 127.49 (2C), 127.41, 124.91 (2C), 122.20, 120.67, 114.97, 85.14, 65.48, 54.20, 43.18; IR (KBr, cm^−1^) ν: 3394, 1675 (C=O), 1607, 1530, 1501, 1466, 1358, 1325, 1263, 1188, 1067, 960, 847, 755; Anal. Calcd. for C_23_H_21_N_3_O_4_: C, 68.47; H, 5.25; N, 10.42; Found: C, 68.13; H, 5.23; N, 10.38.

*1-(Benzylcarbamoylmethyl)-2-(4-nitrophenyl)-2,4-dihydro-1H-3,1-benzoxazine* (**5r**): Eluent: acetate/petroleum ether (*v*/*v* = 1:5); Yield 65%. Yellow solid, m.p.: 147.7–148.5 °C; ^1^H-NMR (500 MHz, CDCl_3_) *δ*: 8.03 (d, *J* = 8.5 Hz, 2H), 7.45 (d, *J* = 8.5 Hz, 2H), 7.14–7.20 (m, 4H), 7.05 (br, 1H, NH), 6.99–7.01 (m, 2H), 6.82–6.85 (m, 2H), 6.77 (d, *J* = 8.5 Hz, 1H), 5.67 (s, 1H, NCHO), 4.81 (d, *J* = 15 Hz, 1H), 4.59 (d, *J* = 15 Hz, 1H), 4.4 (dd, *J* = 15.0, 7.0 Hz, 1H), 4.22 (dd, *J* = 15.0, 5.0 Hz, 1H), 3.86 (d, *J* = 18.0 Hz, 1H), 3.69 (d, *J* = 18.0 Hz, 1H); ^13^C-NMR (125 MHz, CDCl_3_) *δ*: 169.15 (C=O), 148.31, 144.11, 142.51, 137.75, 128.72 (2C), 128.67 (2C), 128.48, 127.69, 127.62 (2C), 124.95, 124.06 (2C), 122.49, 120.89, 115.33, 88.78, 65.06, 54.52, 43.32; IR (KBr, cm^−1^) ν: 3405, 1681 (C=O), 1604, 1518, 1495, 1463, 1425, 1347, 1298, 1074, 1027, 857, 761; Anal. Calcd. for C_23_H_21_N_3_O_4_: C, 68.47; H, 5.25; N, 10.42; Found: C, 68.20; H, 5.23; N, 10.39.

### 3.3. Fungicidal Activity Testing

The in vitro inhibition of mycelium in the agar culture medium caused by the title compounds against six phytopathogenic fungi: *Sclerotonia sclerotiorum*, *Botrytis cinerea*, *Rhizoctonia solani*, *Gibberella zeae, Phytophythora capsic* and *Magnaporthe oryzae* was performed. Referring to the standard method NY/T1156.5–2006, antifungal activity assays adopted the mycelium growth rate test method. Chlorothalonil was used as a reference compound. A stock solution of every test compound was prepared in acetone and then diluted to the required test concentrations (500 µg/mL) with sorporl-144 (concentration: 200 µg/mL). Solutions of the test compounds (1 mL) were added to potato dextrose agar (PDA) medium (9 mL, 45 °C) to provide the final concentration of 25 µg/mL, but 50 µg/mL for *Magnaporthe oryzae*. The mixed medium without sample was used as the blank control. The inocula, 4 mm in diameter, were removed from the margins of actively growing colonies of mycelium and placed in the centers of the above plates. 4 replicates were performed per treatment. Percentages of growth inhibition were calculated by comparing the mean value of the diameters of the mycelia in the test plates after placement in a 24 °C biochemical incubator thermostat for 3 days. The inhibition percent was calculated according to the following equation:

*I* = [(D_1_ − D_0_)/D_1–4_] × 100%

where *I* is the inhibition rate, D_1_ is the average diameter of mycelia the blank test, and D_0_ is the average diameter of mycelia in the presence of compounds. The results are given in Table 2.

## 4. Conclusions

In summary, we have prepared a series of novel 1-(carbamoylmethyl)-2-aryl-3,1-benzoxazines by aza-acetalizations of 2-(*N*-substituted carbamoylmethylamino)benzyl alcohols with aromatic aldehydes in the presence of BF_3_·OEt_2_. The fungicidal activities of the prepared compounds against plant fungi were preliminarily evaluated, and some compounds showed good activities. Compounds **5d** and **5g** showed higher activity against *M. Oryzae* than chlorothalonil. The activity of compound **5i** against *S. Sclerotiorum* is close to that of chlorothalonil.

## Figures and Tables

**Table 1:** The results of the preparation of 3,1-benzoxazines **5a** ^a^.

No.	R	R^1^	Conditions	Product	Yield/% ^b^
1^c^	2-CH_3_C_6_H_4_	3-NO_2_	BF_3_·OEt_2_(10%), 65 °C, 6 hn(**3**):n(**4**) = 1:1.3	**5a**	35
2^c^	2-CH_3_C_6_H_4_	3-NO_2_	BF_3_·OEt_2_(20%), 65 °C, 6 hn(**3**):n(**4**) = 1:1.3	**5a**	45
3	2-CH_3_C_6_H_4_	3-NO_2_	BF_3_·OEt_2_(20%), 65 °C, 6 hn(**3**):n(**4**) = 1:1.3	**5a**	47
4	2-CH_3_C_6_H_4_	3-NO_2_	BF_3_·OEt_2_(20%), 65 °C, 8 hn(**3**):n(**4**) = 1:1.3	**5a**	50
5	2-CH_3_C_6_H_4_	3-NO_2_	BF_3_·OEt_2_(20%), 65 °C, 10 hn(**3**):n(**4**) = 1:1.3	**5a**	53
6	2-CH_3_C_6_H_4_	3-NO_2_	BF_3_·OEt_2_(20%), 65 °C, 14 hn(**3**):n(**4**) = 1:1.3	**5a**	44
7	2-CH_3_C_6_H_4_	3-NO_2_	BF_3_·OEt_2_(20%), 65 °C, 10 hn(**3**):n(**4**) = 1:1.5	**5a**	55
8	2-CH_3_C_6_H_4_	2-NO_2_	BF_3_·OEt_2_(20%), 65 °C, 10 h	**5b**	45
9	2-CH_3_C_6_H_4_	4-NO_2_	BF_3_·OEt_2_(20%), 65 °C, 10 h	**5c**	49
10	4-CH_3_C_6_H_4_	3-NO_2_	BF_3_·OEt_2_(20%), 65 °C, 10 h	**5d**	56
11	4-CH_3_C_6_H_4_	2-NO_2_	BF_3_·OEt_2_(20%), 65 °C, 10 h	**5e**	40
12	4-CH_3_C_6_H_4_	4-NO_2_	BF_3_·OEt_2_(20%), 65 °C, 10 h	**5f**	48
13	4-CH_3_OC_6_H_4_	3-NO_2_	BF_3_·OEt_2_(20%), 65 °C, 10 h	**5g**	85
14	4-CH_3_OC_6_H_4_	2-NO_2_	BF_3_·OEt_2_(20%), 65 °C, 10 h	**5h**	66
15	4-CH_3_OC_6_H_4_	4-NO_2_	BF_3_·OEt_2_(20%), 65 °C, 10 h	**5i**	80
16	3-CH_3_OC_6_H_4_	3-NO_2_	BF_3_·OEt_2_(20%), 65 °C, 10 h	**5j**	74
17	3-CH_3_OC_6_H_4_	2-NO_2_	BF_3_·OEt_2_(20%), 65 °C, 10 h	**5k**	41
18	3-CH_3_OC_6_H_4_	4-NO_2_	BF_3_·OEt_2_(20%), 65 °C, 10 h	**5l**	60
19	C_6_H_5_	3-NO_2_	BF_3_·OEt_2_(20%), 65 °C, 10 h	**5m**	52
20	C_6_H_5_	2-NO_2_	BF_3_·OEt_2_(20%), 65 °C, 10 h	**5n**	44
21	C_6_H_5_	4-NO_2_	BF_3_·OEt_2_(20%), 65 °C, 10 h	**5o**	46
22	C_6_H_5_CH_2_	3-NO_2_	BF_3_·OEt_2_(20%), 65 °C, 10 h	**5p**	56
23	C_6_H_5_CH_2_	2-NO_2_	BF_3_·OEt_2_(20%), 65 °C, 10 h	**5q**	58
24	C_6_H_5_CH_2_	4-NO_2_	BF_3_·OEt_2_(20%), 65 °C, 10 h	**5r**	65

^a^ Unless mentioned in the table, the reaction conditions were: the mole ratio of n (**3**):n (**4**) = 1:1.5; BF_3_·OEt_2_: 20 mol % based on compound **3**; Solvent: THF; Molecular sieve 4Å added; Reaction time: 10 h; Temperature: 65 °C. ^b^ Isolated yield. ^c^ Without molecular sieve 4Å.

**Table 2:** Fungicidal activities of compounds **5a**–**r**.

Compd.	*S. sclerotiorum*/%	*B. cinerea*/%	*R. solani*/%	*G. zeae*/%	*P. capsici*/%	*M. oryzae*/% ^a^
**5a**	33.3	43.8	21.1	12.5	7.7	18.2
**5b**	33.3	37.5	18.4	37.5	30.8	27.3
**5c**	33.3	31.3	5.3	12.5	15.4	13.6
**5d**	14.3	43.8	26.3	31.3	15.4	60.1
**5e**	14.3	31.3	21.1	6.3	38.5	36.4
**5f**	47.6	56.3	28.9	31.3	15.4	22.7
**5g**	57.1	12.5	21.1	25.0	15.4	54.5
**5h**	23.8	18.8	31.6	31.3	38.5	22.7
**5i**	71.9	25.0	31.6	12.5	15.4	31.8
**5j**	14.3	12.5	18.4	37.5	30.8	22.7
**5k**	42.9	25.0	21.1	12.5	7.7	22.7
**5l**	23.8	43.8	31.6	12.5	15.4	27.3
**5m**	14.3	50.0	26.3	37.5	15.4	27.3
**5n**	28.6	37.5	18.4	18.8	38.5	22.7
**5o**	27.3	23.8	51.7	53.8	33.3	51.7
**5p**	11.6	11.5	19.6	17.1	11.1	41.2
**5q**	46.5	38.5	25.5	17.1	29.6	41.2
**5r**	16.3	30.8	15.7	12.2	11.1	11.8
Chlorothalonil ^b^	84.9	92.9	85.2	67.6	78.6	53.8

^a^ The value measured at concentration of 50 µg/mL. ^b^ Chlorothalonil used as reference compound.

## Supplementary Materials

Supplementary materials are available online: ^1^H-NMR and ^13^C-NMR Data for compounds **3a**–**3f**, **5a**–**5r.**

## References

[1] Kuch H., Schmitt K., Seudl G., Hoffmann I. (1973). 2-Amino-4,4-di-substituted-4*H*-3,1-benzoxazines. U.S. Patent.

[2] Kobzina J.W., Creek W. (1977). Herbicidal *N*-Haloacetyl-1,2-dihydro-4*H*-3,1-benzoxazine. U.S. Patent.

[3] Sugiyama H., Hosoda K., Kumagai Y., Takeuchi M., Okada M. (1986). 4*H*-3,1-Benzoxazine Derivatives, Process for Producing the Same and Agricultural or Horticultural Fungicide Containing the Same. U.S. Patent.

[4] Tang Z., Wang L., Huang T., Gao W., Luo X. (2015). The Fungicidal Activities and Application of 1-(Carbamoylmethyl)-2-aryl-2,4-dihydro-3,1-benzoxazines.

[5] Charmantray F., Demeunynck M., Carrez D., Croisy A., Lansiaux A., Bailly C., Colson P. (2003). 4-Hydroxymethyl-3-aminoacridine derivatives as a new family of anticancer agents. J. Med. Chem..

[6] Zhang P., Terefenko E.A., Fensome A., Zhang Z., Zhu Y., Cohen J., Winneker R., Wrobel J., Yardley J. (2002). Potent nonsteroidal progesterone receptor agonists: Synthesis and SAR study of 6-aryl-benzoxazines. Bioorg. Med. Chem. Lett..

[7] Dias N., Goossens J., Baldeyrou B., Lansiaux A., Colson P., Di Salvo A., Bernal J., Turnbull A., Mincher D.J., Bailly C. (2005). Oxoazabenzo[de]anthracenes conjugated to amino acids: Synthesis and evaluation as DNA-binding antitumor agents. Bioconj. Chem..

[8] Clark R.D., Caroon J.M., Kluge A.F., Repke D.B., Roszkowski A.P., Strosberg A.M., Baker S., Bitter S.M., Okada M.D. (1983). Synthesis and antihypertensive activity of 4′-substituted spiro[4*H*-3,1-benzoxazine-4,4’-piperidin]-3(1*H*)-ones. J. Med. Chem..

[9] Badolato M., Carullo G., Armentano B., Panza S., Malivindi R., Aiello F. (2017). Synthesis and anti-proliferative activity of a small library of 7-substituted 5*H*-pyrrole [1,2-*a*][3,1]benzoxazin-5-one derivatives. Bioorg. Med. Chem. Lett..

[10] Zhang P., Terefenko E.A., Fensome A., Wrobel J., Winneker R., Lundeen S., Marschke K.B., Zhang Z. (2002). 6-Aryl-1,4-dihydro-benzo[*d*][1,3]oxazin-2-ones: A novel class of potent, selective, and orally active nonsteroidal progesterone receptor antagonists. J. Med. Chem..

[11] Kern J.C., Terefenko E.A., Fensome A., Unwallla R., Wrobel J., Zhu Y., Cohen J., Winneker R., Zhang Z., Zhang P. (2007). SAR studies of 6-(arylamino)-4,4-disubstituted-1-methyl-1,4-dihydro-benzo[*d*][1,3]-oxazin-2-ones as progesterone. Bioorg. Med. Chem. Lett..

[12] Krantz A., Spencer R.W., Tam T.F., Liak T.J., Copp L.J., Thomas E.M., Rafferty S.P. (1990). Design and synthesis of 4*H*-3,1-benzoxazin-4-one as potent alternate substrate inhibitors of human leukocyte elastase. J. Med. Chem..

[13] Gilmore J.L., Hays S.J., Caprathe B.W., Lee C., Emmerling M.R., Micheal W., Jean J.C. (1996). Synthesis and valuation of 2-aryl-4*H*-3,1-benzoxazin-4-ones as C1r serine protease inhibitors. Bioorg. Med. Chem. Lett..

[14] Hays S.J., Caprathe B.W., Gilmore J.L., Amin N., Emmerling M.R., Micheal W., Nadimpalli R., Nath R., Raser K.J., Stafford D. (1998). 2-Amino-4*H*-3,1-benzoxazi-4-ones as C1r serine protease. J. Med. Chem..

[15] Gutschow M., Neumann U., Sieler J., Eger K. (1998). Studies on 2-benzyloxy-4*H*-3,1-bnezoxazin-4-ones as serine protease inhibitors. Pharm. Acta Helv..

[16] Mizutani T., Ishikawa S., Nagase T., Takahashi H., Fujimura T., Sasaki T., Nagumo A., Shimamura K., Miyamoto Y., Kitazawa H. (1990). Discovery of novel benzoxazinones as potent and orally active long chain fatty acid elongase 6 inhibitors. J. Med. Chem..

[17] Hanessian S., Jennequin T., Boyer N., Babonneau V., Soma U., La Cour C.M., Millan M.J., De Nanteuil G. (2014). Design, synthesis, and optimization of balanced dual NK_1_/NK_3_ receptor antagonists. ACS Med. Chem. Lett..

[18] Marasini B.P., Rahim F., Perveen S., Karim A., Khan K.M., Rahman A., Choudhary M.L. (2017). Synthesis, structure-activity relationships studies of benzoxazine derivatives as *α*-chymoitrypsin inhibitor. Bioorg. Chem..

[19] Martin-Martinez M., Perez-Gordillo F., Alvarez de la Rosa D., Rodriguez Y., Gerona-Navarro G., Gonzalez-Muniz R., Zhou M.-M. (2017). Modulating mineralocorticoid receptor with non-steroidal antagonists. New opportunities for the development of potent and selective ligands without off-target side effects. J. Med. Chem..

[20] Patel M., Ko S.S., McHugh R.J., Markwalder J.A., Srivasta A.S., Cordova B.C., Klabe R.M., Erickson-Viitanen S., Trainor G.L., Seitz S.P. (1999). Synthesis and evaluation of analogs of Efavirenz (Sustiva^TM^) as HIV-1 reverse transcriptase inhibitors. Bioorg. Med. Chem. Lett..

[21] Cocuzza A.J., Chidester D.R., Cordova B.C., Jeffrey S., Parsons R.L., Bacheler L.T., Erickson-Viitanen S. (2001). Synthesis and evaluation of Efavirenz (Sustiva^TM^) analogues as HIV-1 reverse transcriptase inhibitors: Replacement of the cyclopropylacetylene side chain. Bioorg. Med. Chem. Lett..

[22] Holly F.W., Cope A.C. (1944). Condensation products of aldehydes and ketones with *o*-aminobenzyl alcohol and *o*-hydroxybenzylamine. J. Am. Chem. Soc..

[23] Nguyen T.T., Amey R.L., Martin J.C. (1982). Tridentate ligand useful in stabilizing coordination states of nonmetallic elements. An aryldialkoxydifluoroperiodinane. J. Org. Chem..

[24] Saito T., Ogawa S., Takei N., Kutsumura N., Otani T. (2011). Palladium-catalyzed highly regio- and stereoselective synthesis of 4-alkylidene-4*H*-3,1-benzoxazines from *N*-acyl-*o*-alkynylanilines. Org. Lett..

[25] Zhang N., Cheng R., Zhang-Negrerie D., Du Y., Zhao K. (2014). Hypervalent iodine- mediated oxygenation of *N*,*N*-diaryl tertiary amines: Intramolecular functionalization of sp^3^ C-H bonds adjacent to nitrogen. J. Org. Chem..

[26] Lagu B., Pio B., Lebedev R., Yang M., Pelton P.D. (2007). RXR-LXR heterodimer modulators for the potential treatment of dyslipidemia. Bioorg. Med. Chem. Lett..

[27] Ishida T., Kikuchi S., Tsubo T., Yamada T. (2013). Silver-catalyzedincorporation of carbon dioxide into *o*-alkynylanilides derivatives. Org. Lett..

[28] Tang Z., Chen W., Zhu Z., Liu H. (2011). Synthesis of 2,3-diaryl-3,4-dihydro-2*H*-1,3-benzoxazines and their fungicidal activities. J. Heterocycl. Chem..

[29] Tang Z., Zhu Z., Xia Z., Liu H., Chen J., Xiao W., Ou X. (2012). Synthesis and fungicidal activity of novel 2,3-disubstituted-1,3-benzoxazines. Molecules.

