# Supplementary material for: Synthesis and Fungicidal Activity of 1-(Carbamoylmethyl)-2-aryl-3,1-benzoxazines
Source: Molecules. 2017 Jul 3;22(7):1103. doi: 10.3390/molecules22071103 (PMC6152388; doi:10.3390/molecules22071103)
Supplement: Supplementary file 1 [file molecules-22-01103-s001.pdf]

Supporting Information for

Synthesis and Fungicidal Activity of 1-(Carbamoylmethyl)-2-aryl-3,1-benzoxazines

Zi-Long Tang[1,2,*] , Lian Wang[1,2], Jing-Zhao Tan[1,2] , Yi-Chao Wan[1] , Yin-Chun Jiao[2]

1 Key Laboratory of Theoretical Organic Chemistry and Functional Molecule of Ministry of Education, Hunan University of Science and Technology, Xiangtan 411201, China;

2 School of Chemistry and Chemical Engineering, Hunan University of Science and Technology, Xiangtan 411201, China

Table of contents

1 ^{1}H NMR and ^{13}C NMR Data for compounds **3a-3f** P2-P13

2 ^{1}H NMR and ^{13}C NMR Data for compounds **5a-5r** P14-P49

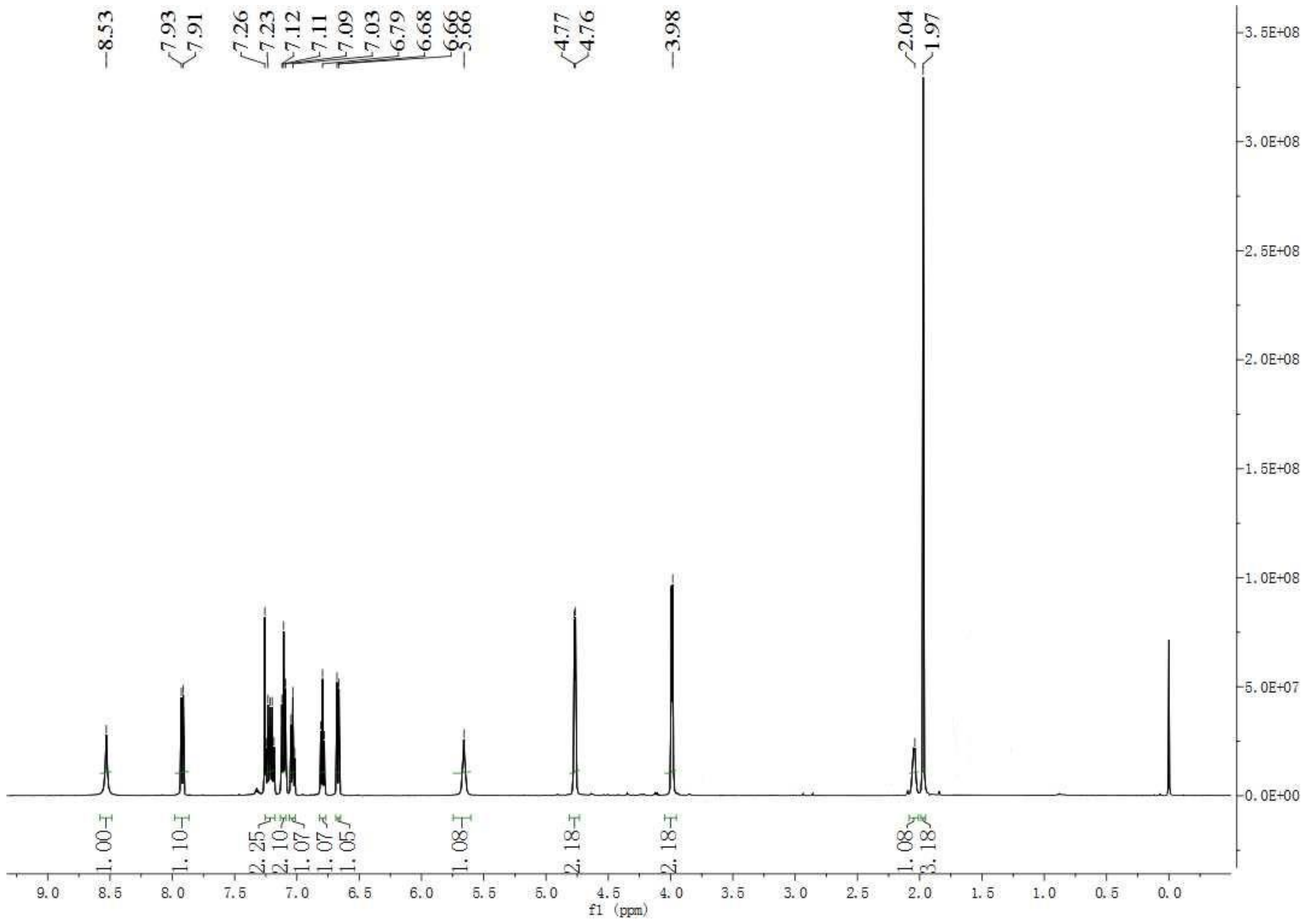

^{1}H NMR of compound **3a**

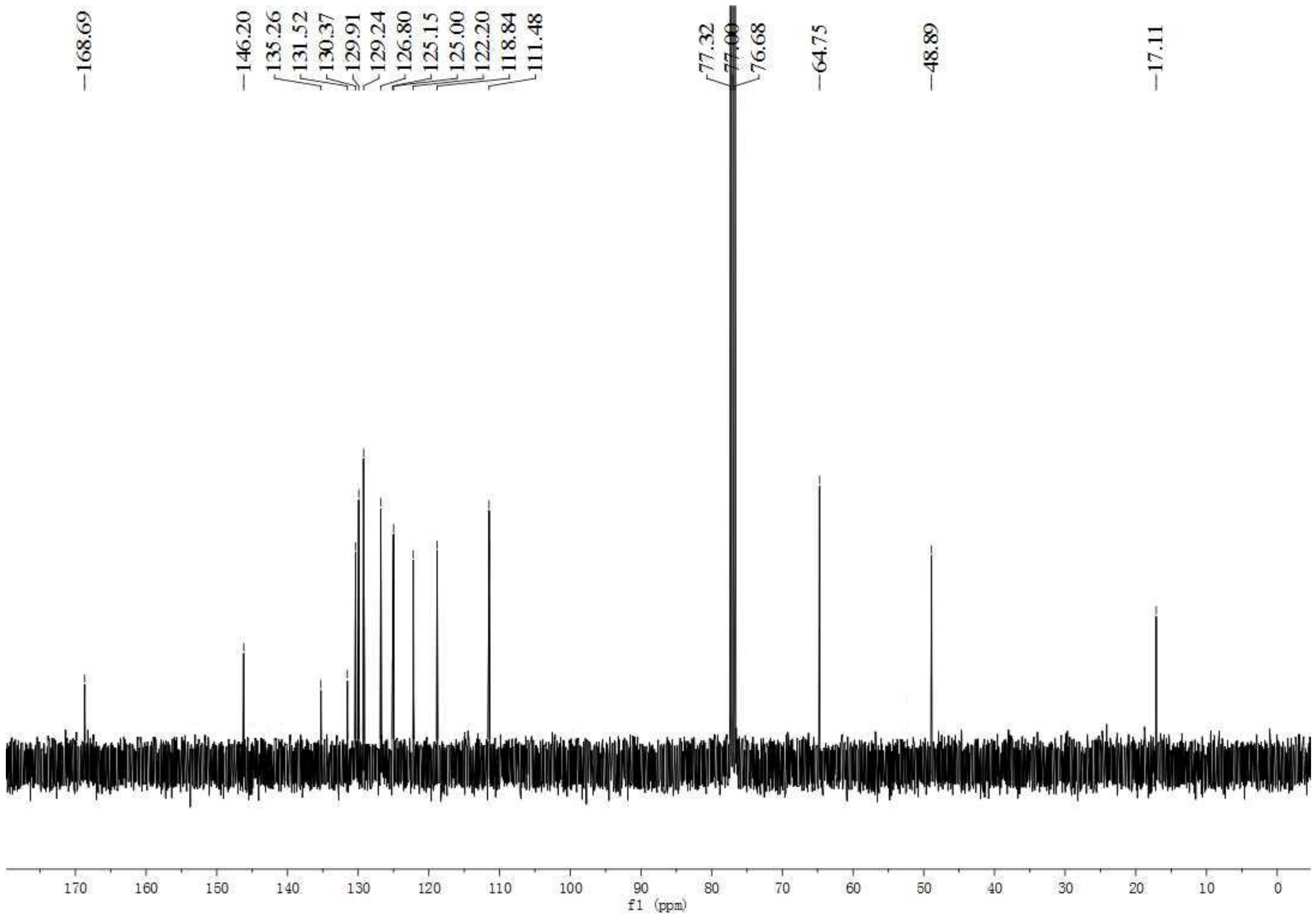

^{13}C NMR of compound **3a**

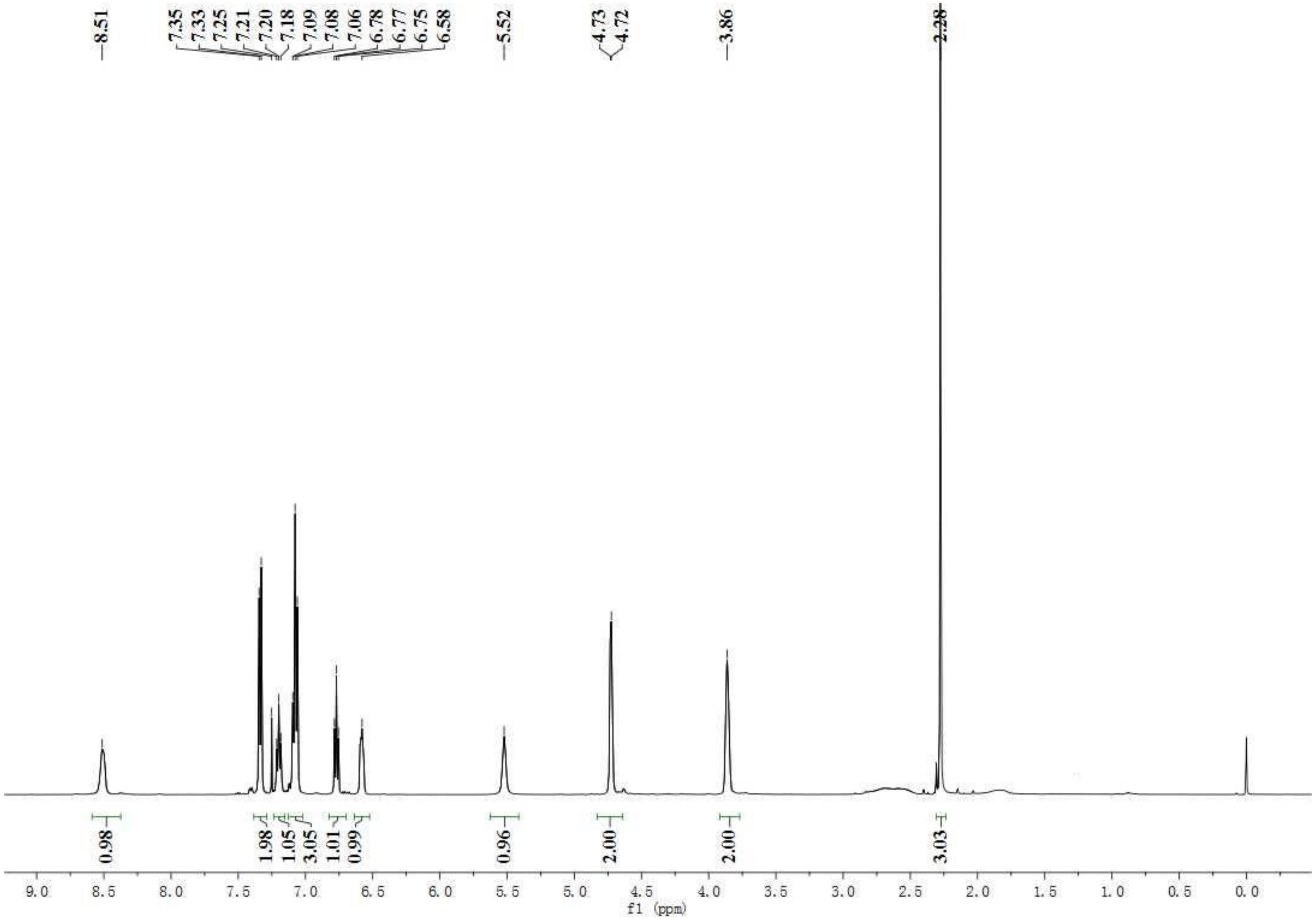

^{1}H NMR of compound **3b**

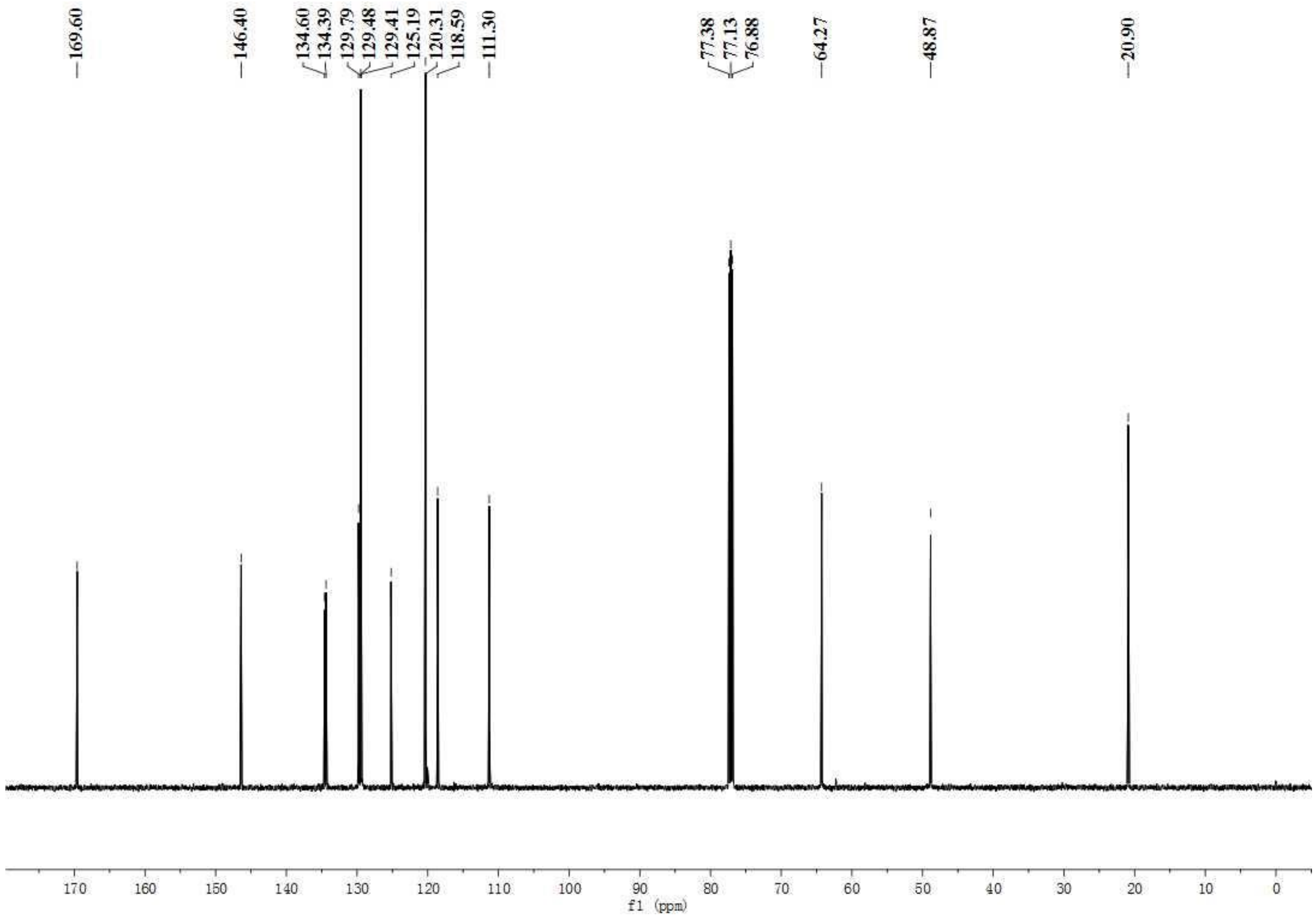

^{13}C NMR of compound **3b**

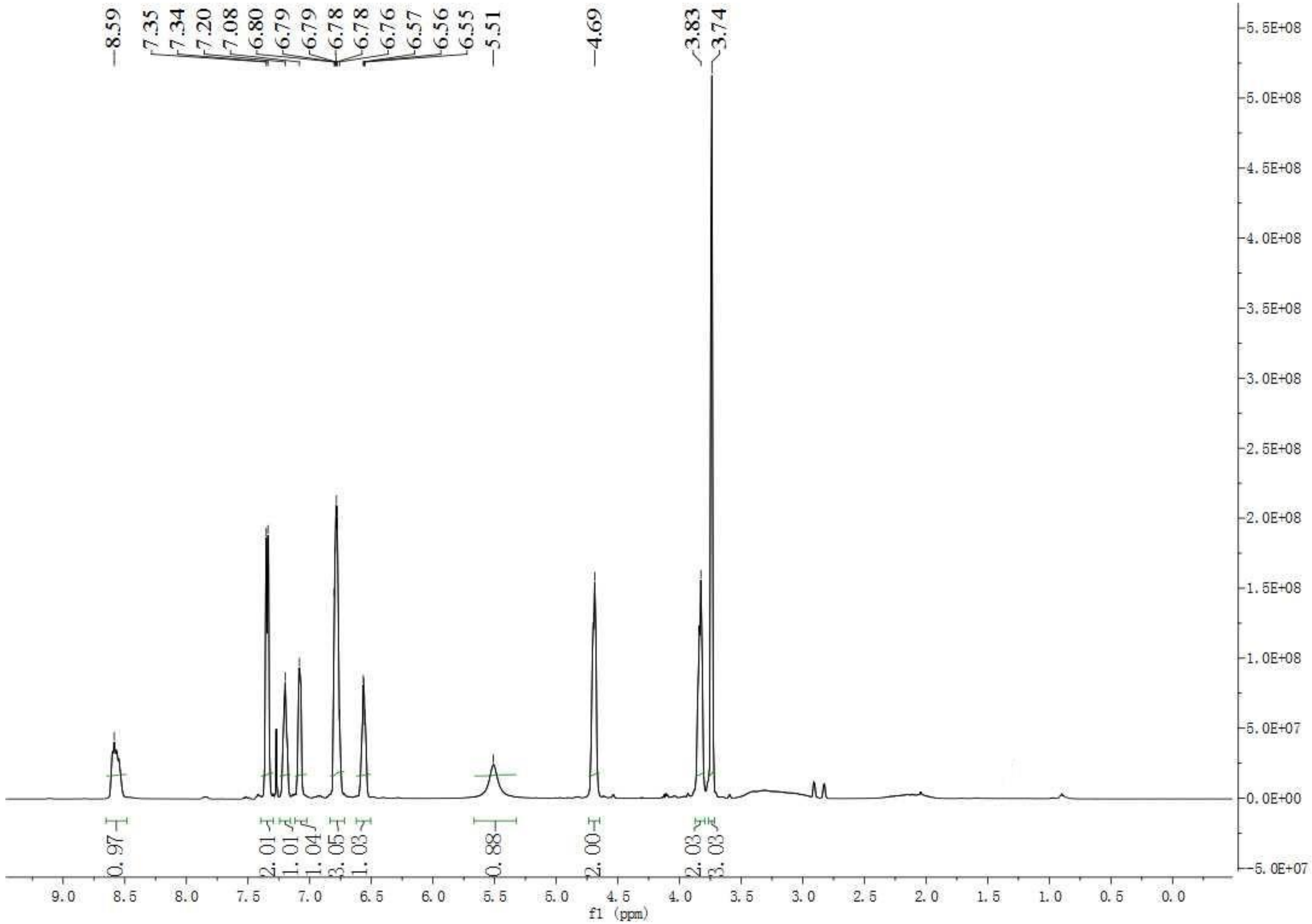

^{1}H NMR of compound **3c**

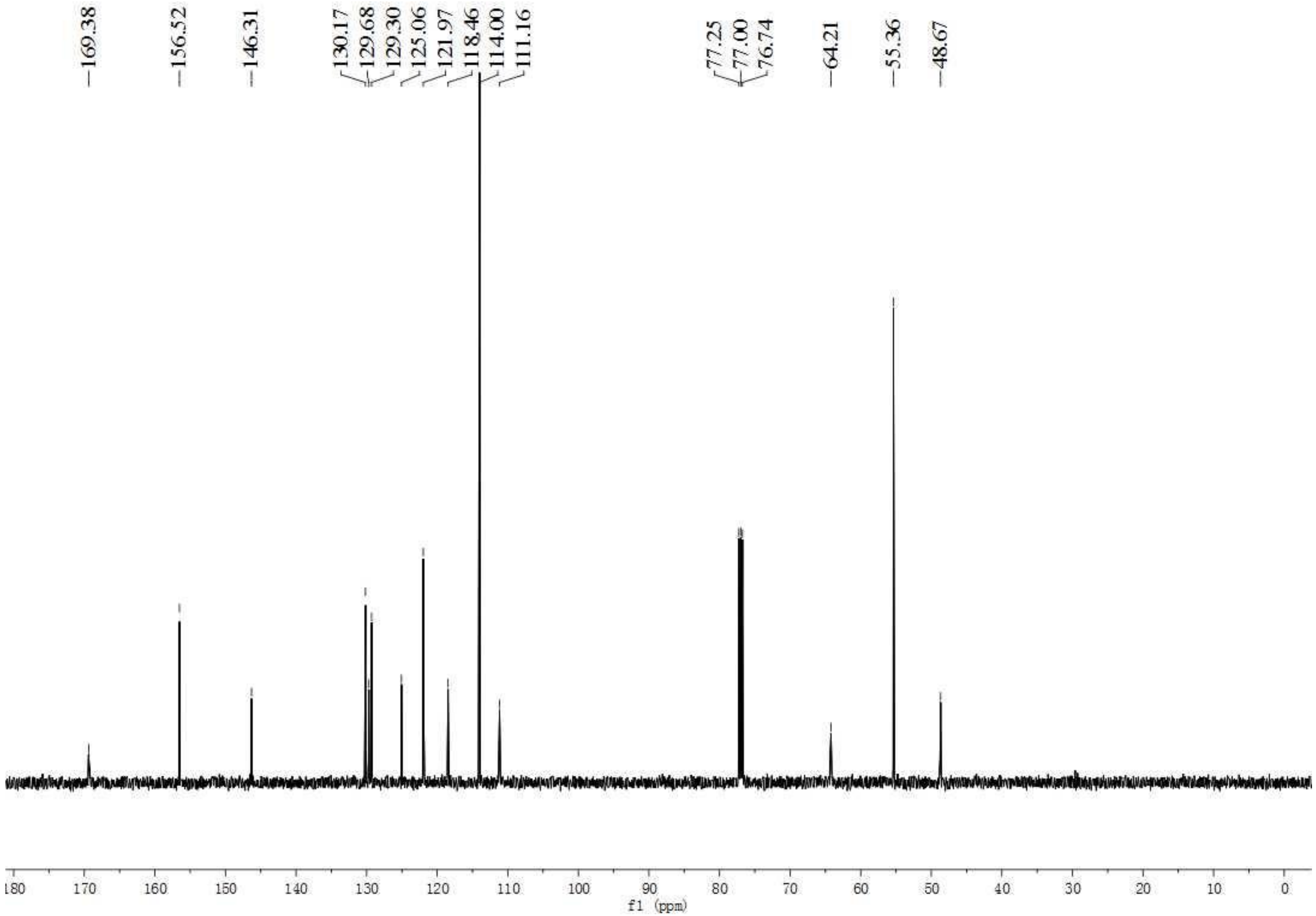

^{13}C NMR of compound **3c**

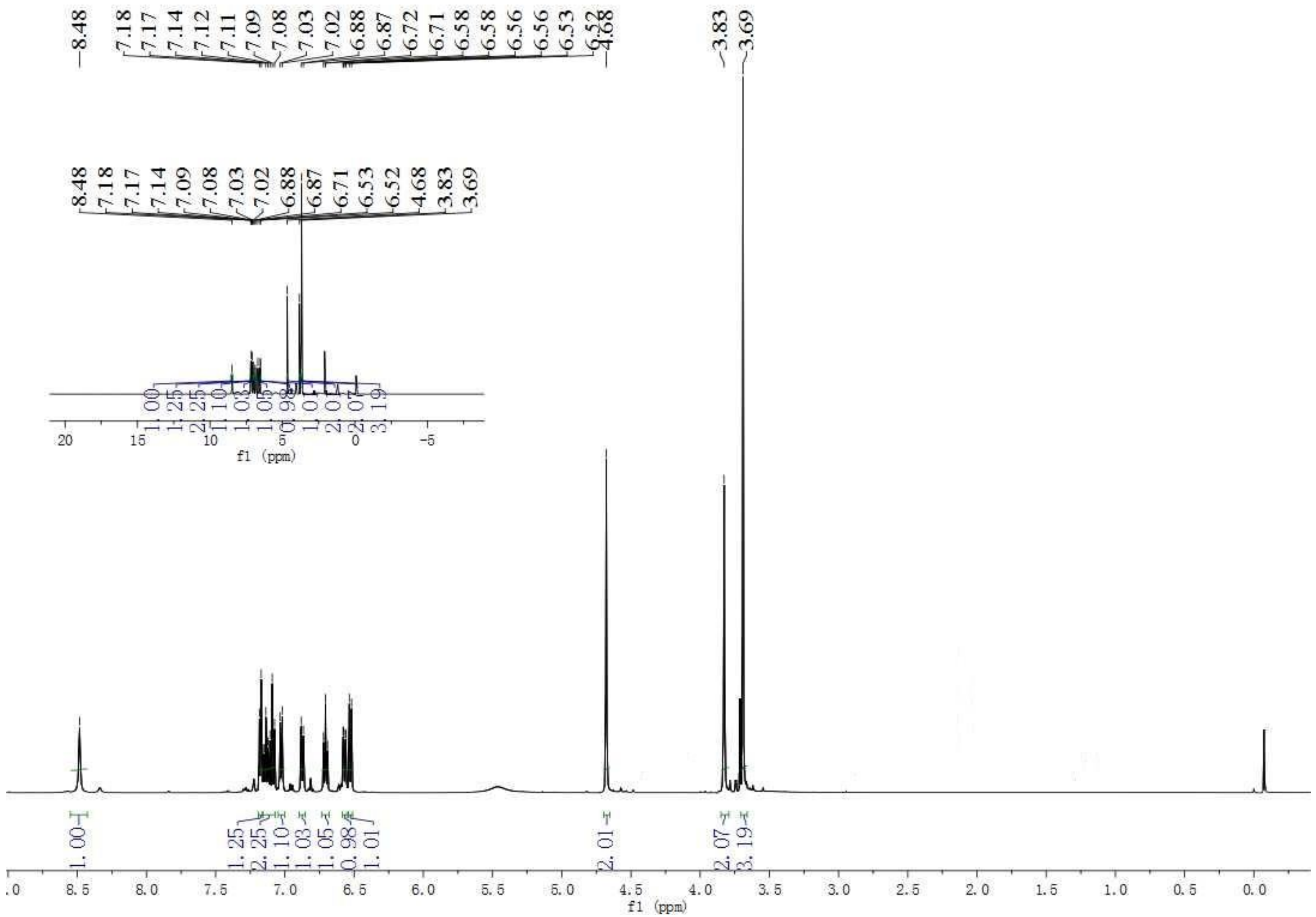

^{1}H NMR of compound **3d**

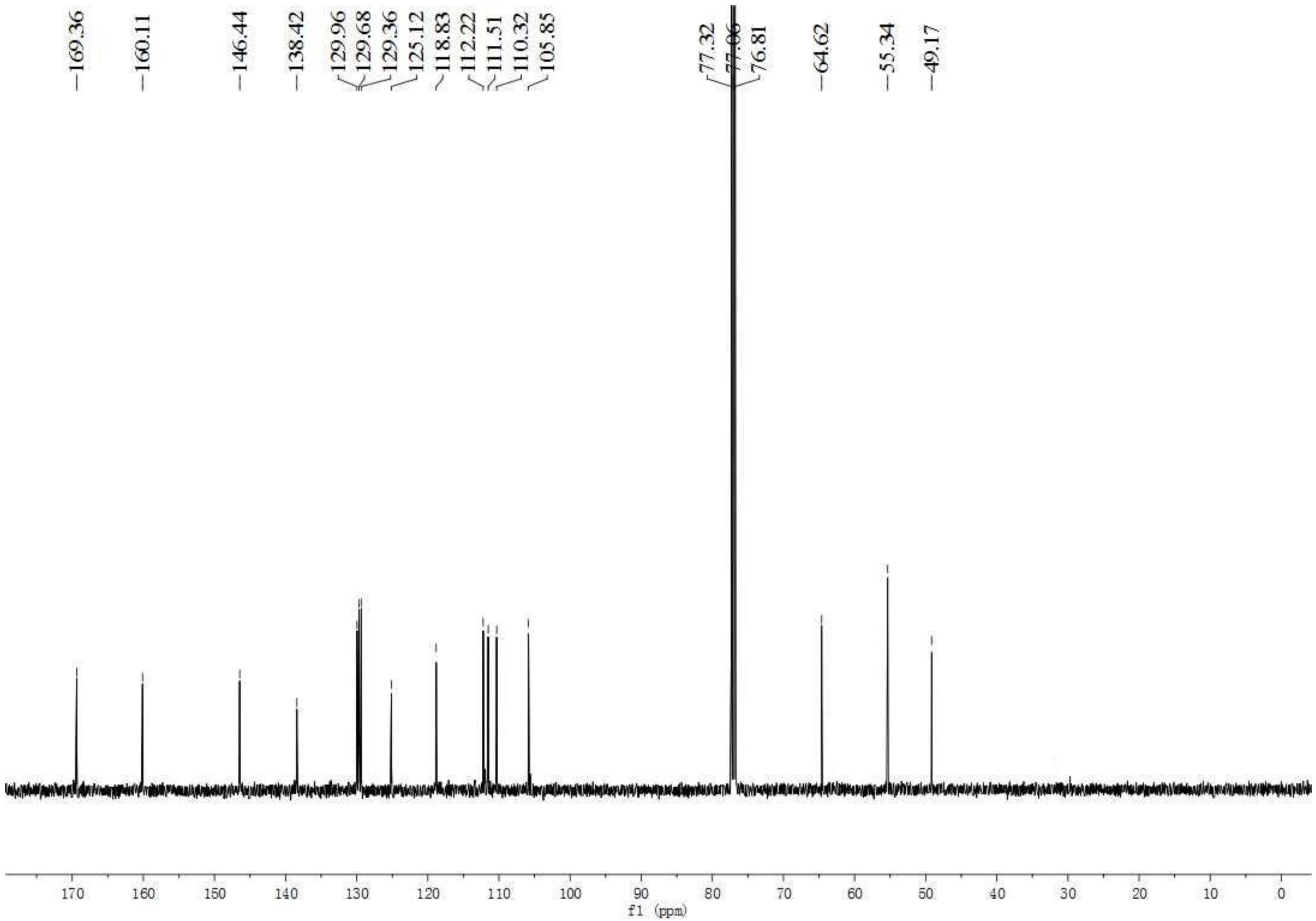

^{13}C NMR of compound **3d**

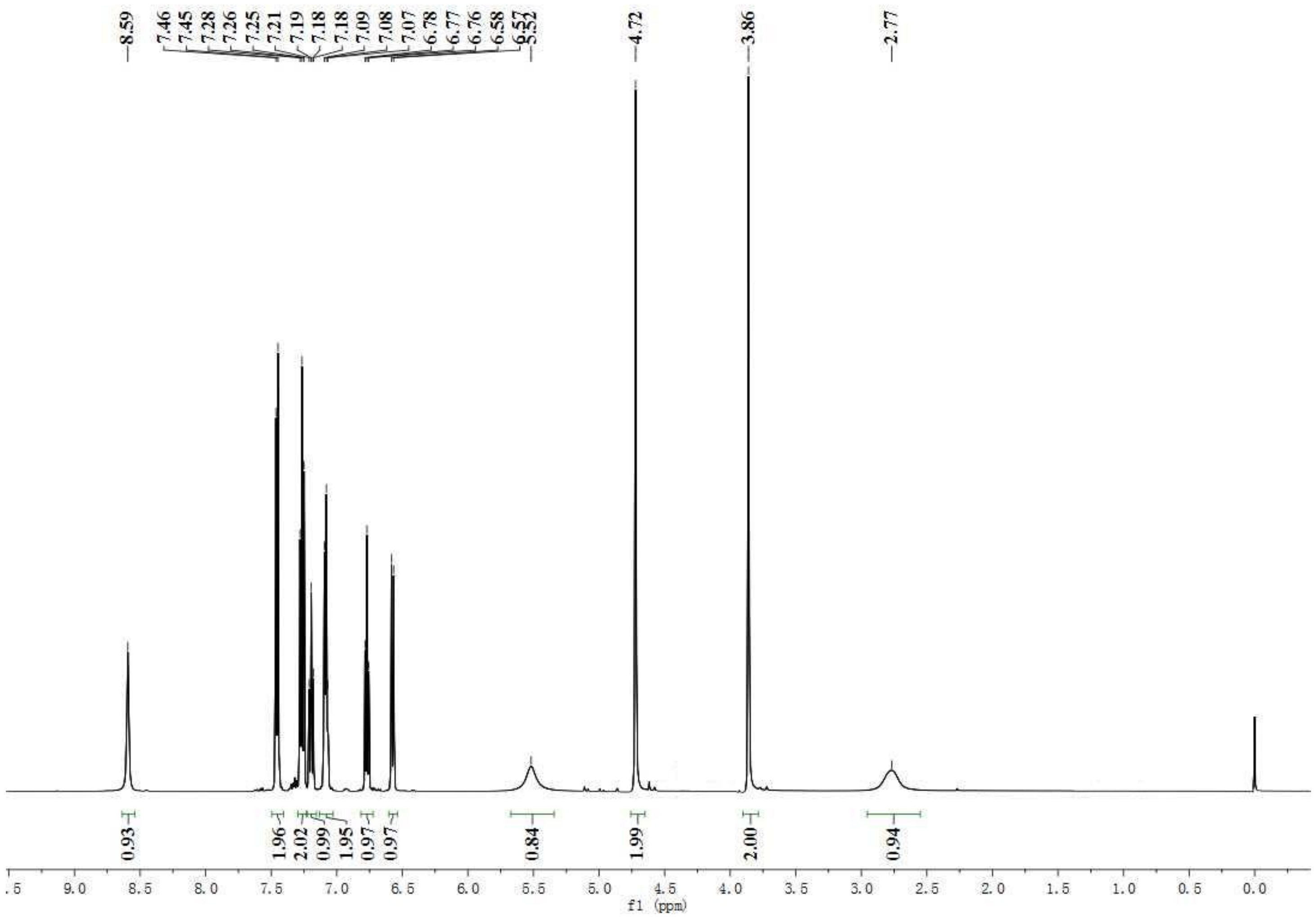

^{1}H NMR of compound **3e**

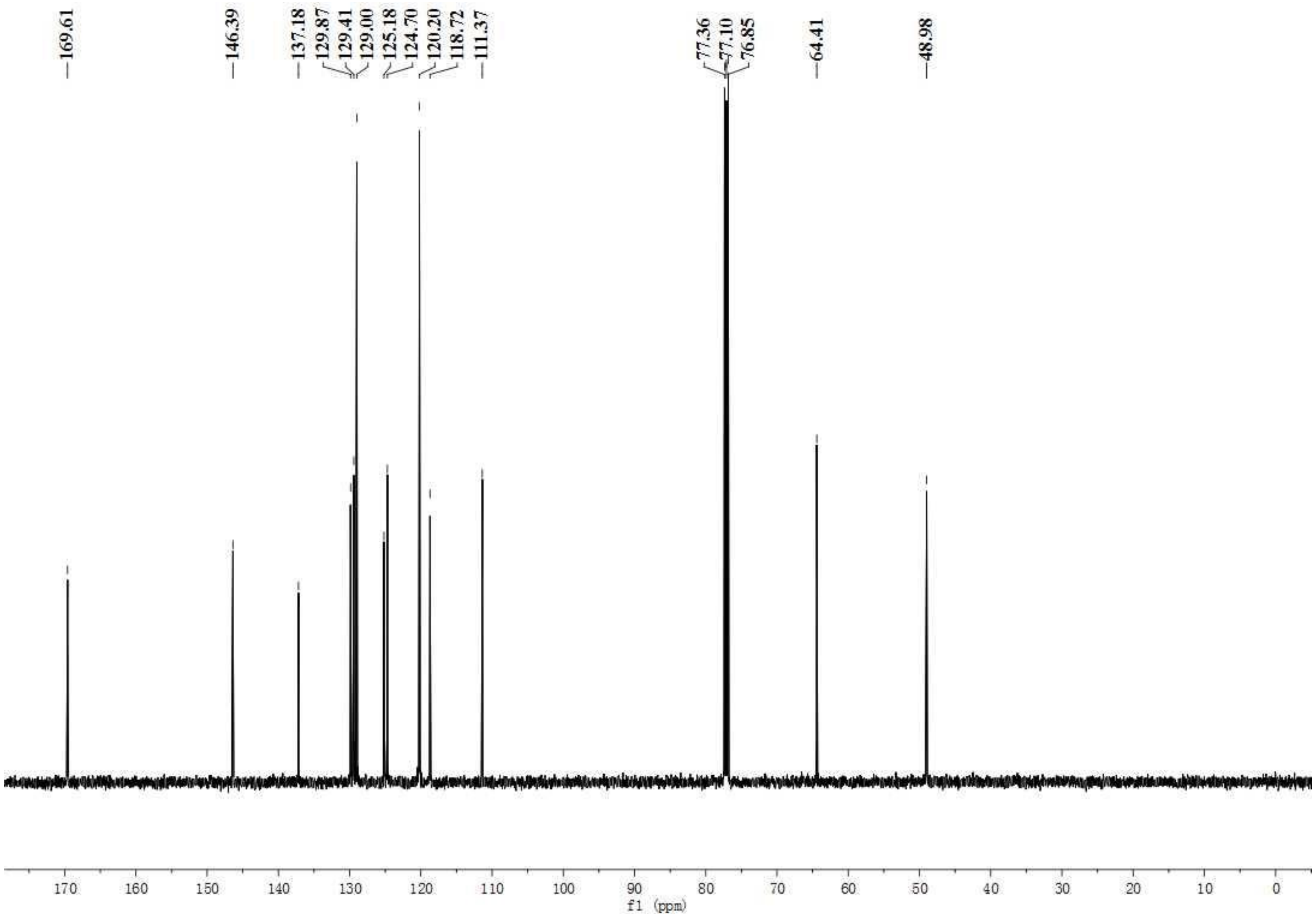

^{13}C NMR of compound **3e**

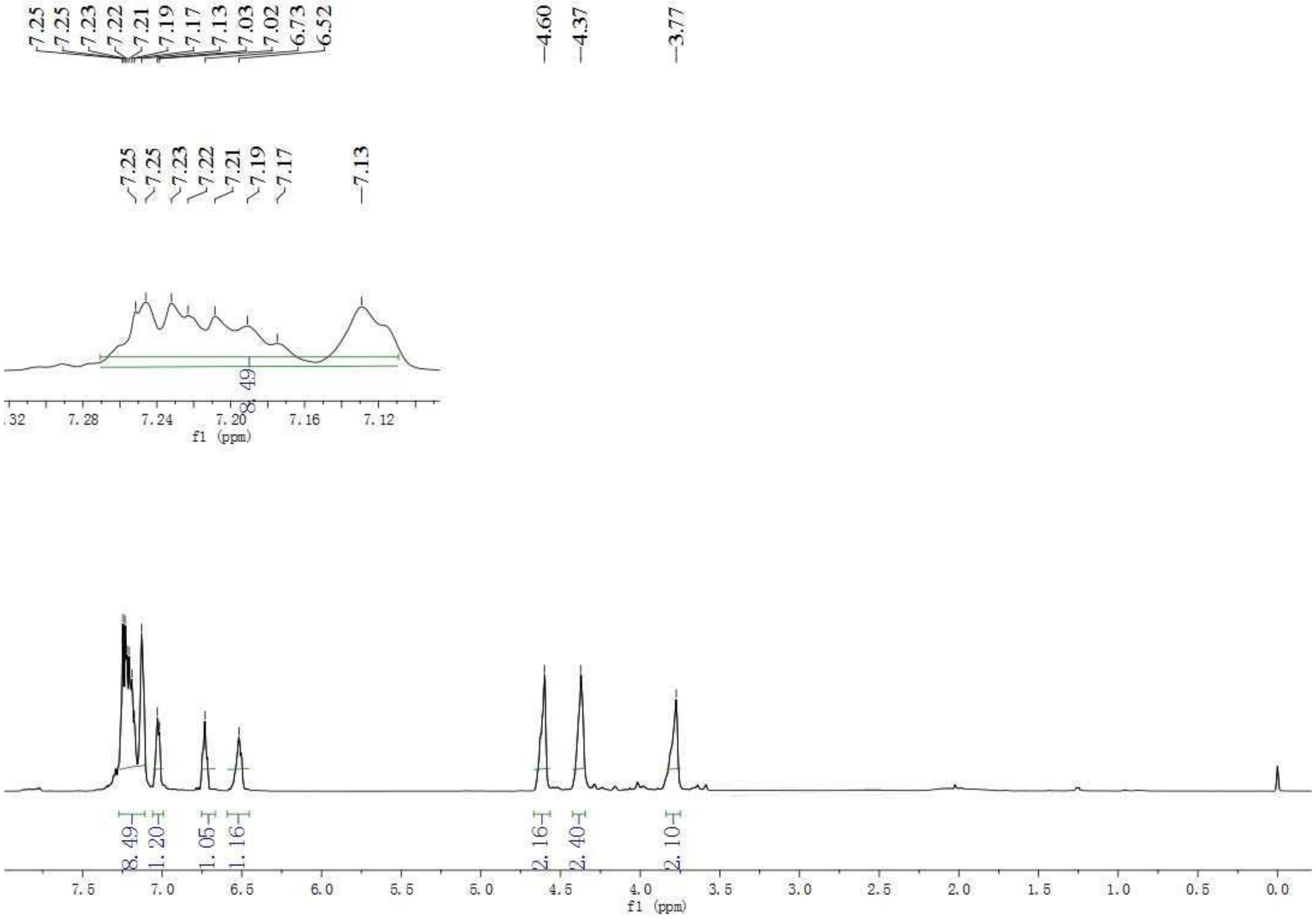

^{1}H NMR of compound **3f**

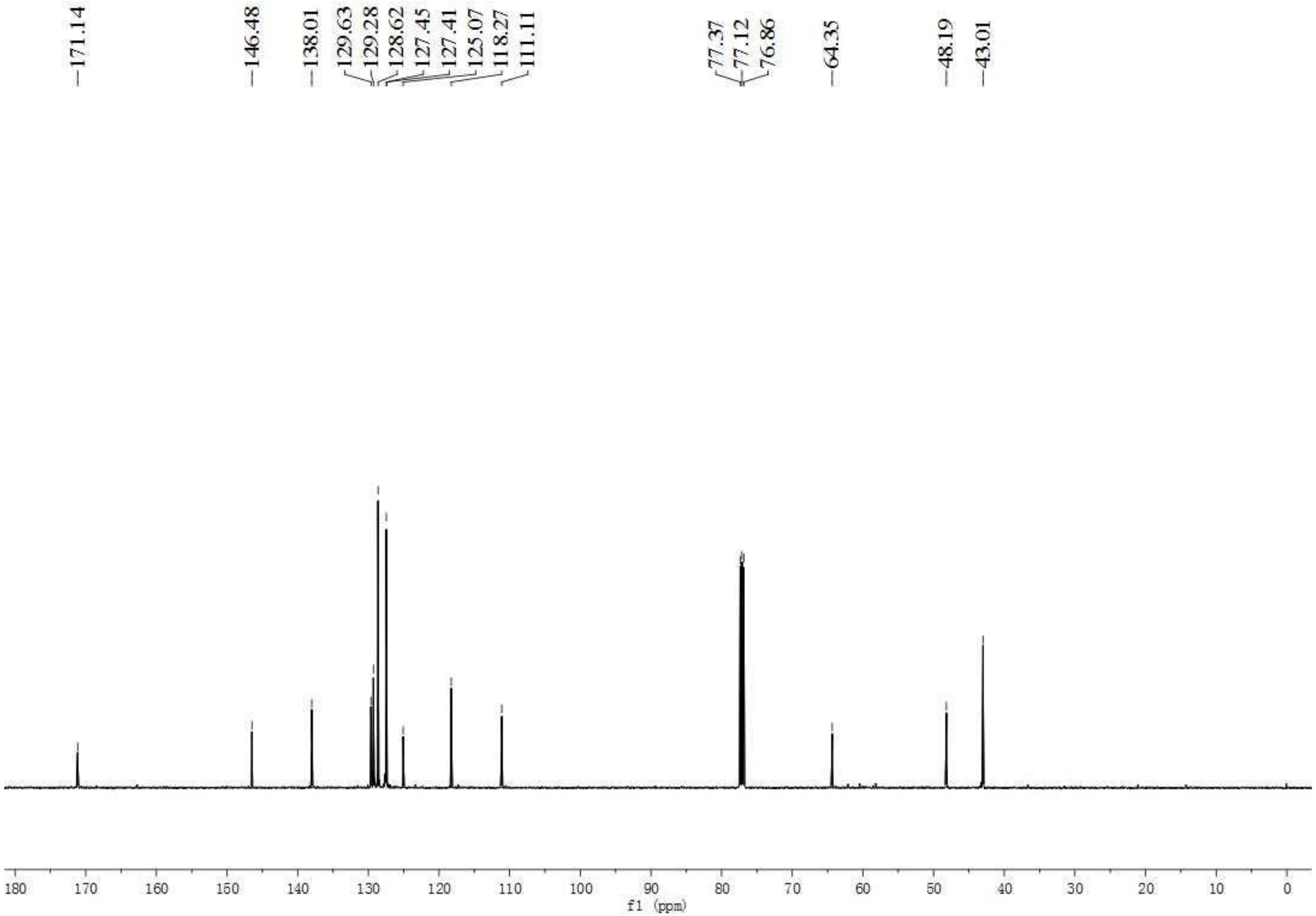

^{13}C NMR of compound **3f**

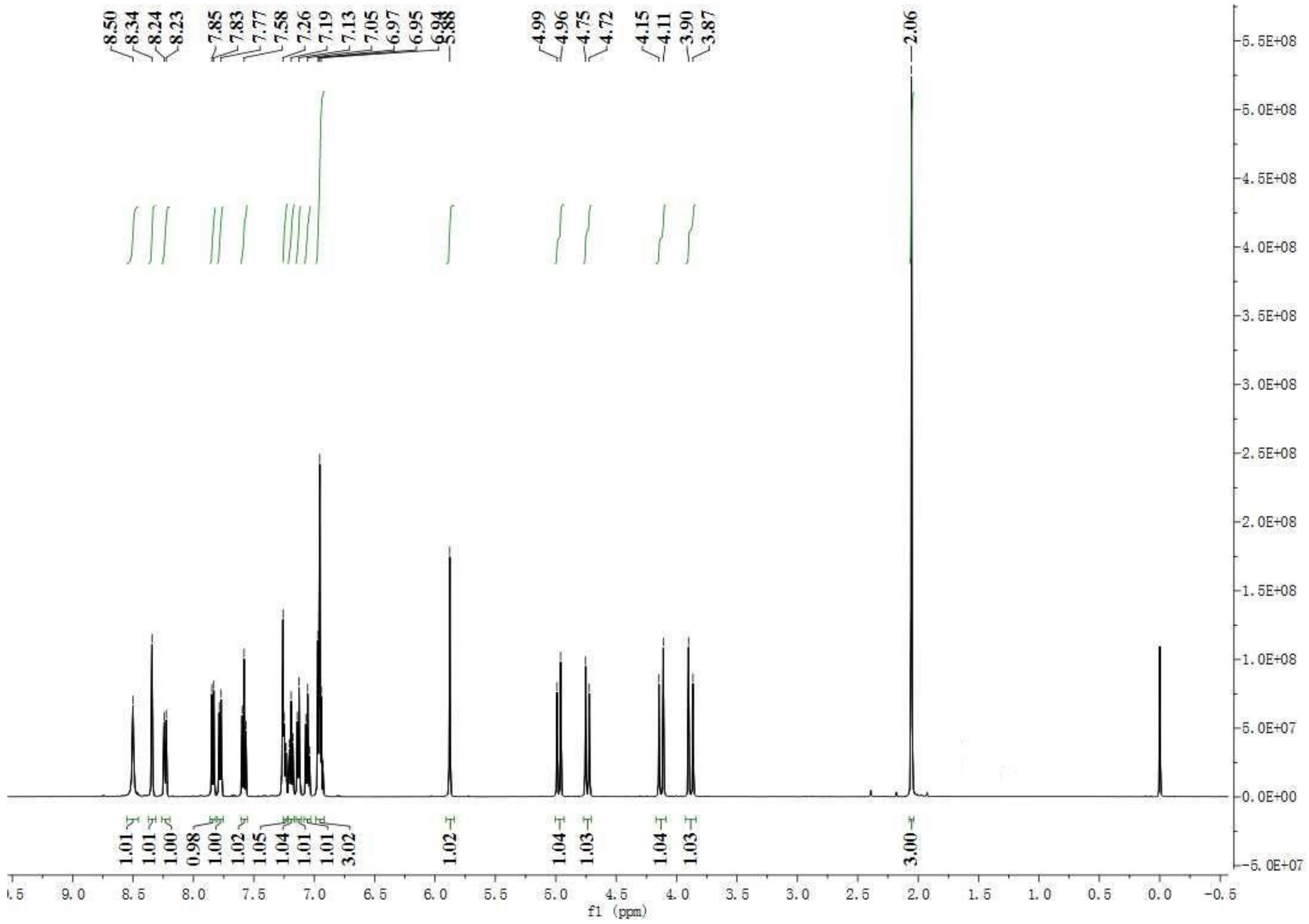

^{1}H NMR of compound **5a**

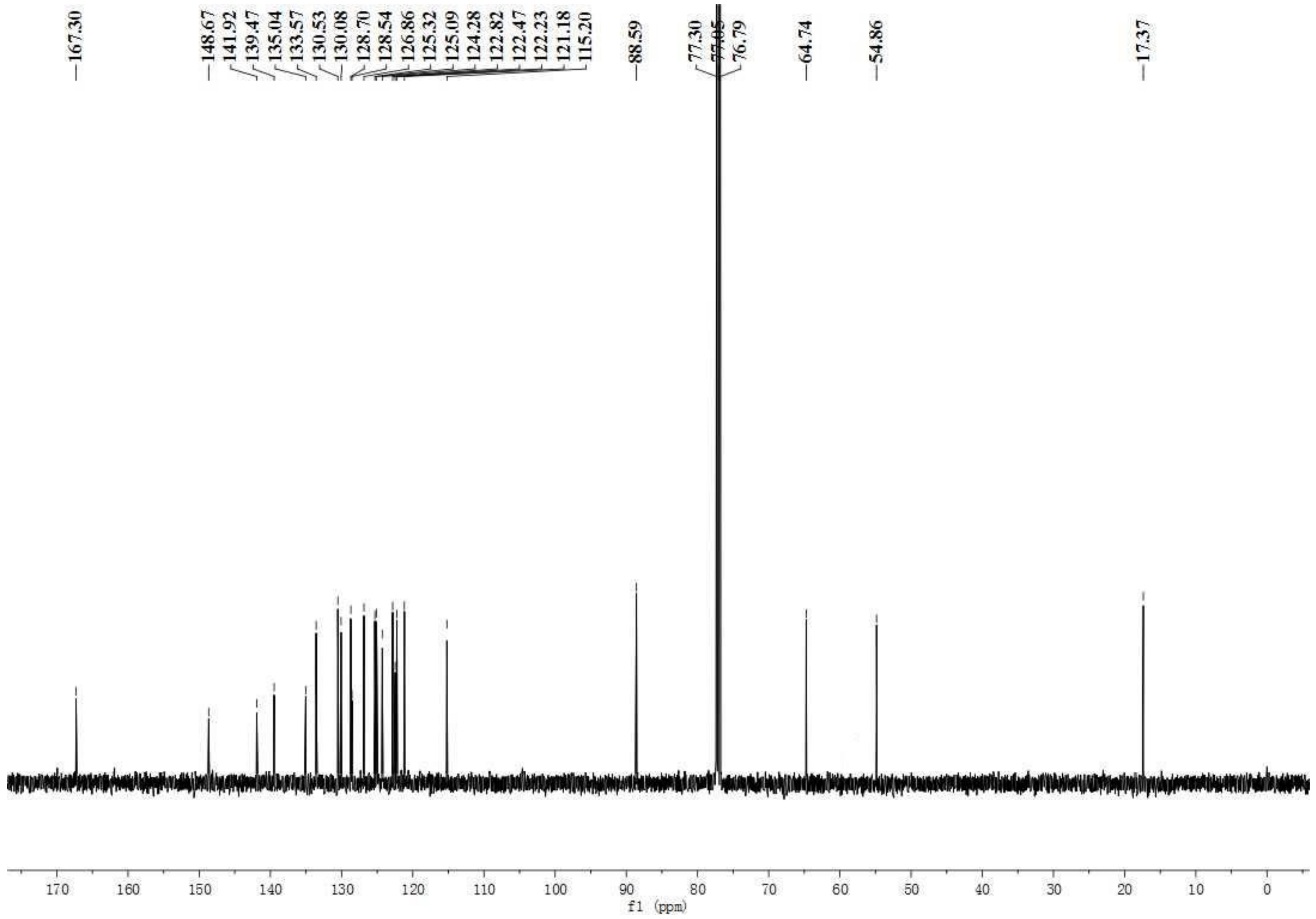

^{13}C NMR of compound **5a**

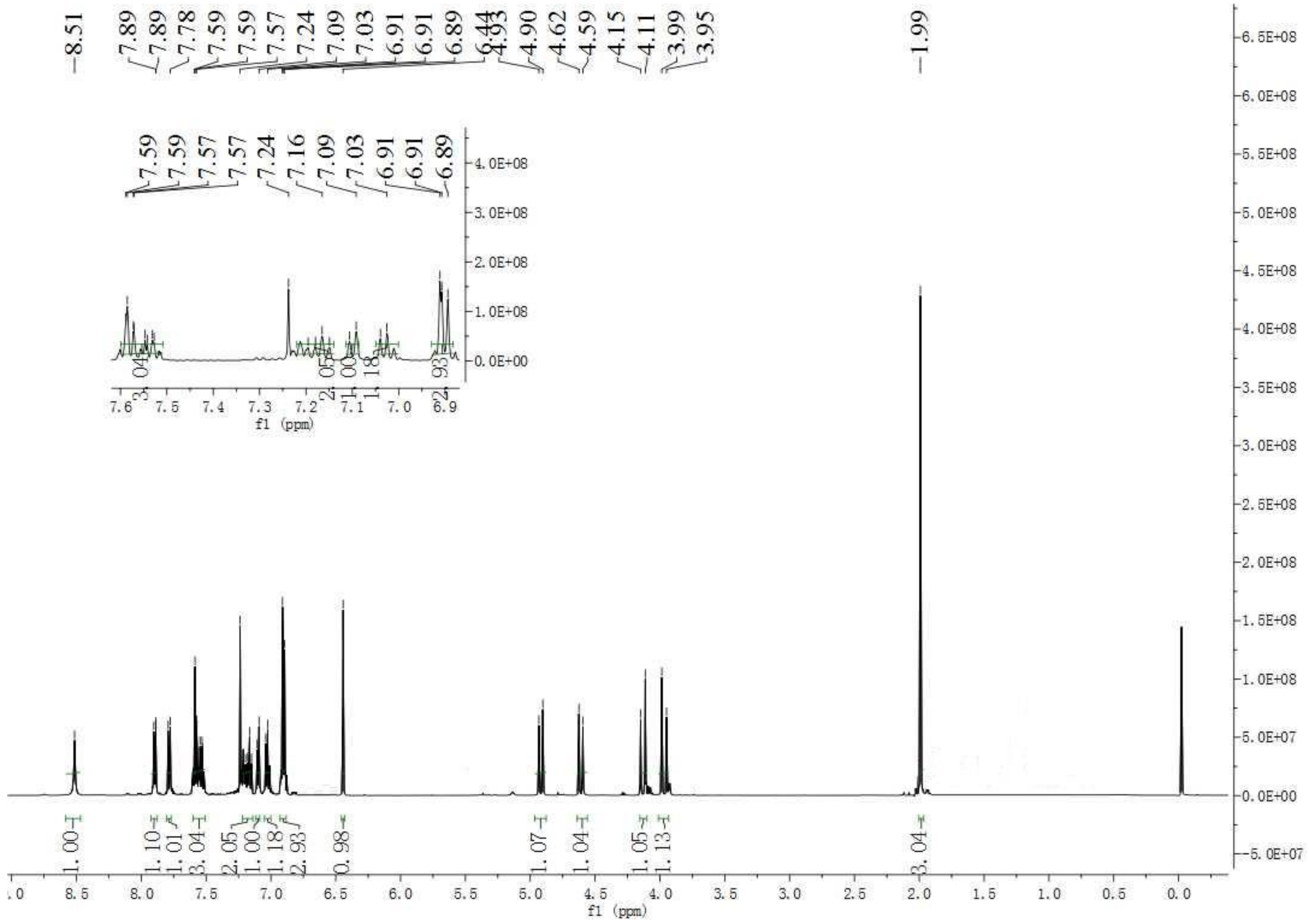

^{1}H NMR of compound **5b**

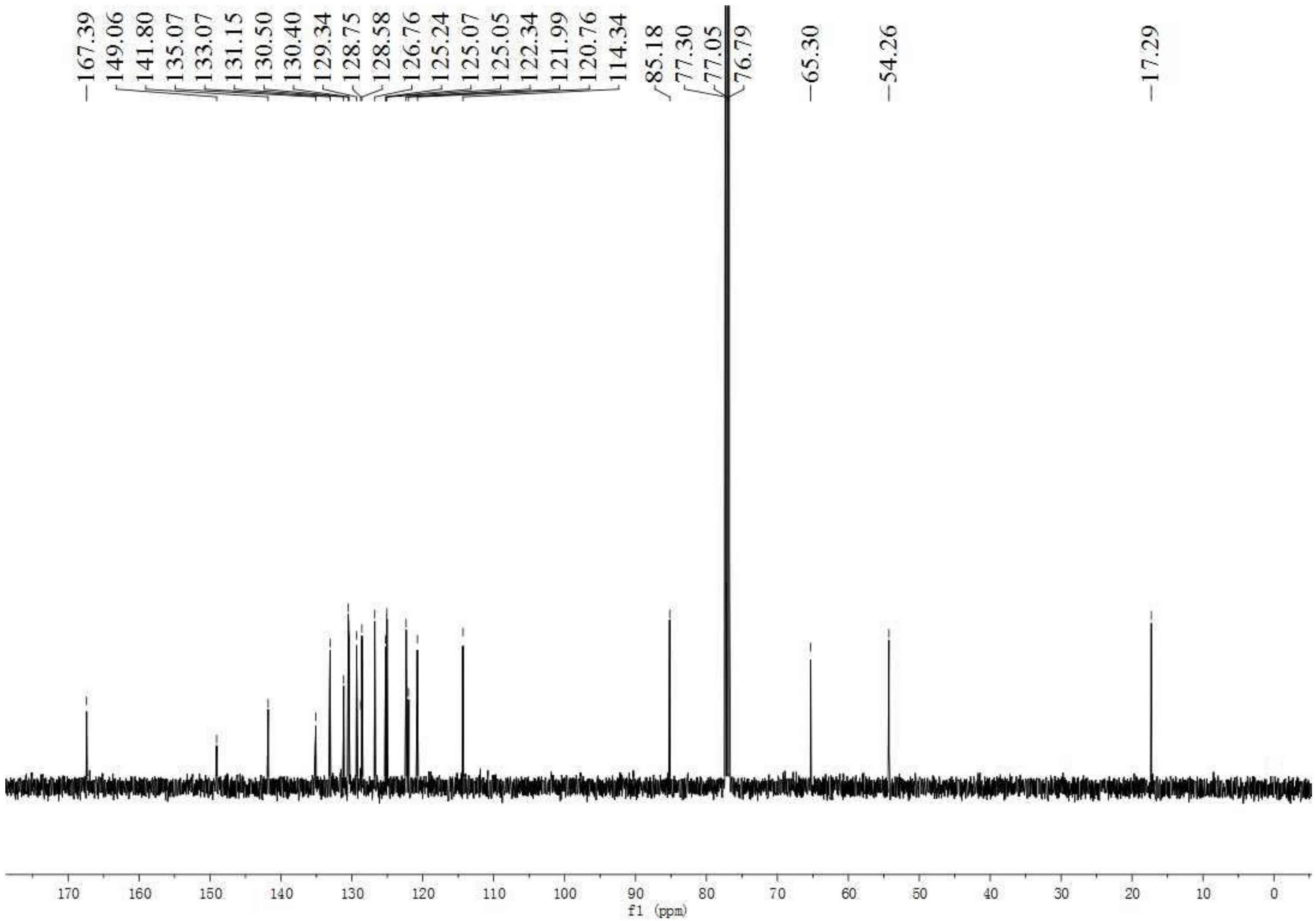

^{13}C NMR of compound **5b**

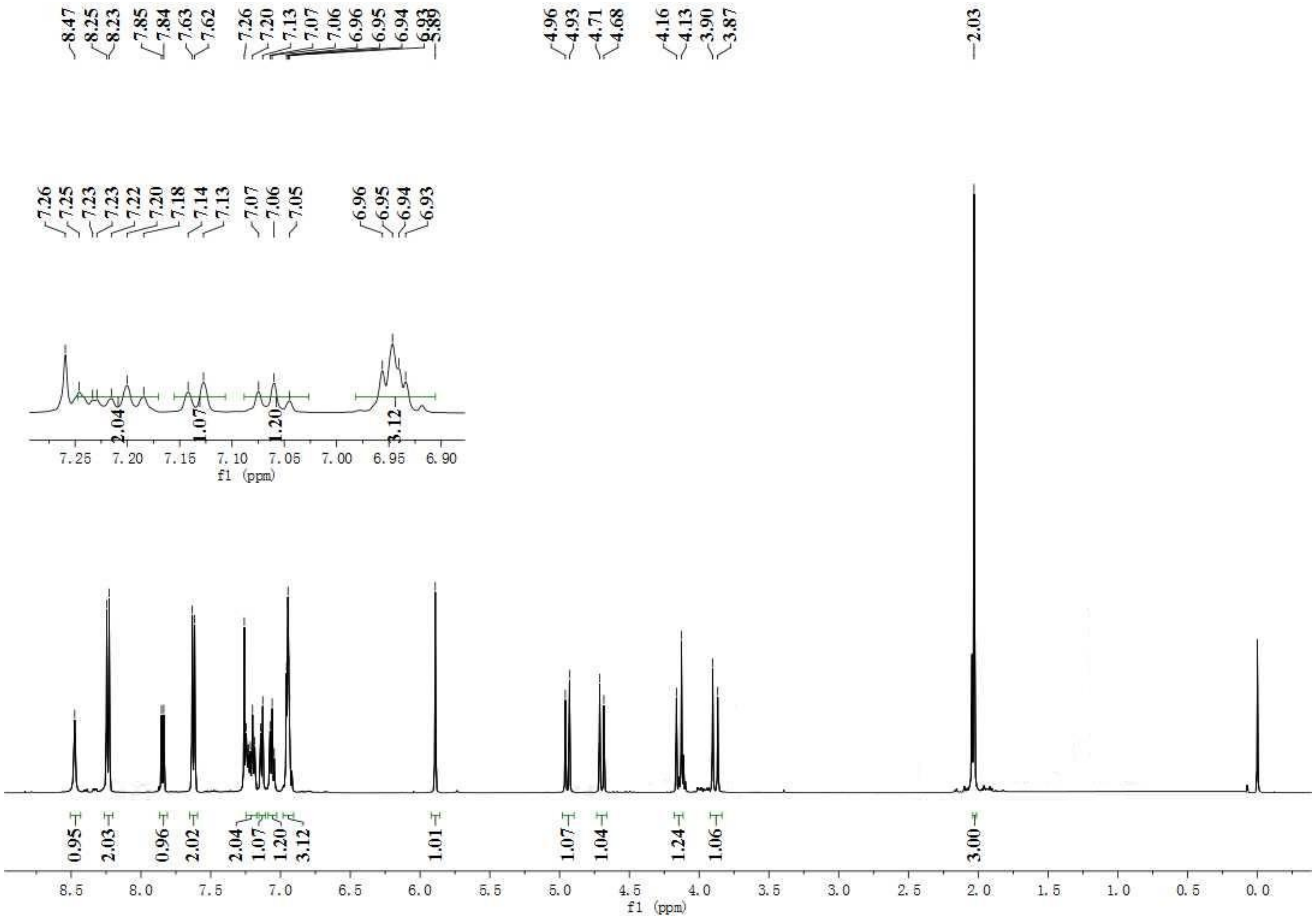

^{1}H NMR of compound **5c**

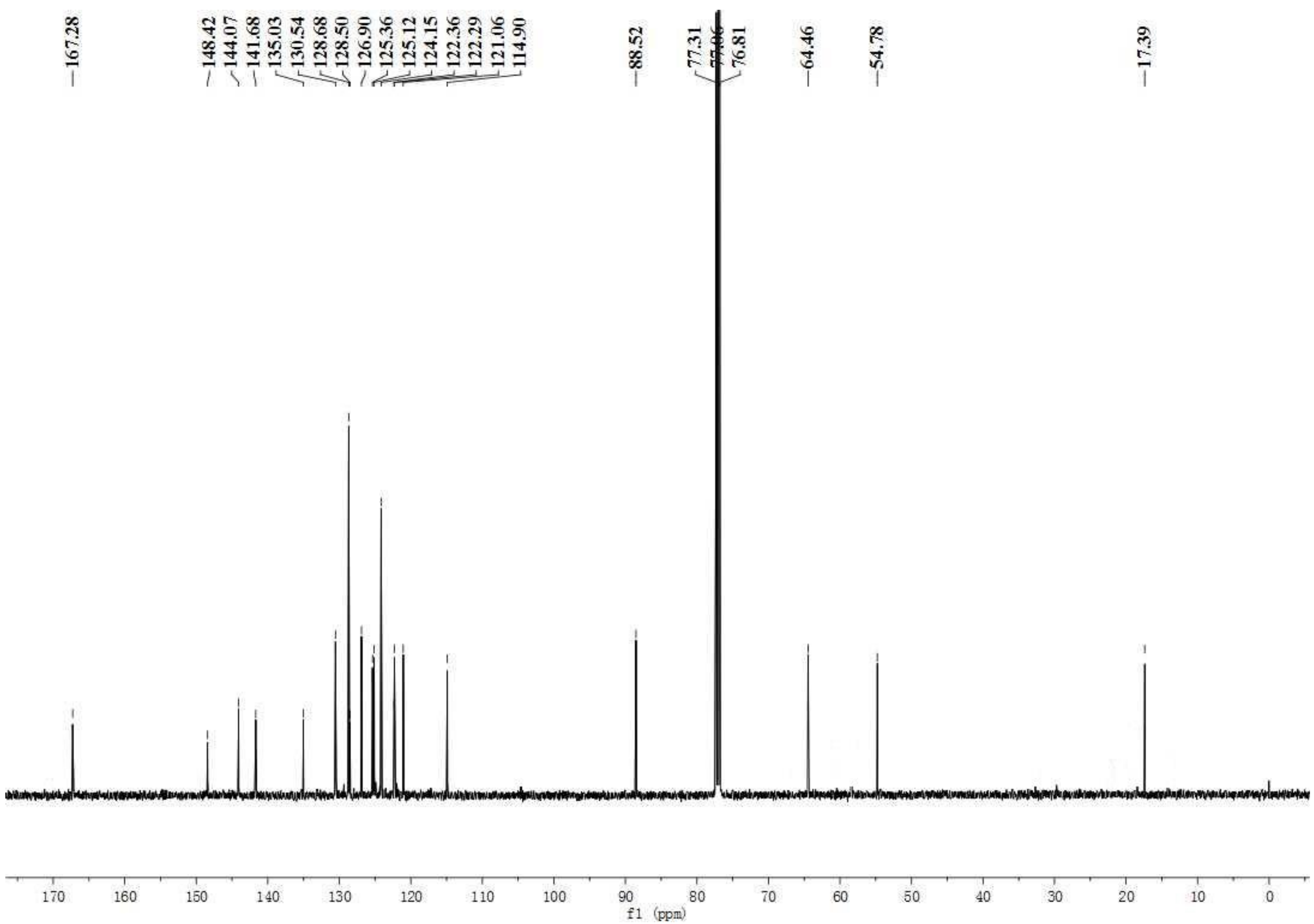

^{13}C NMR of compound **5c**

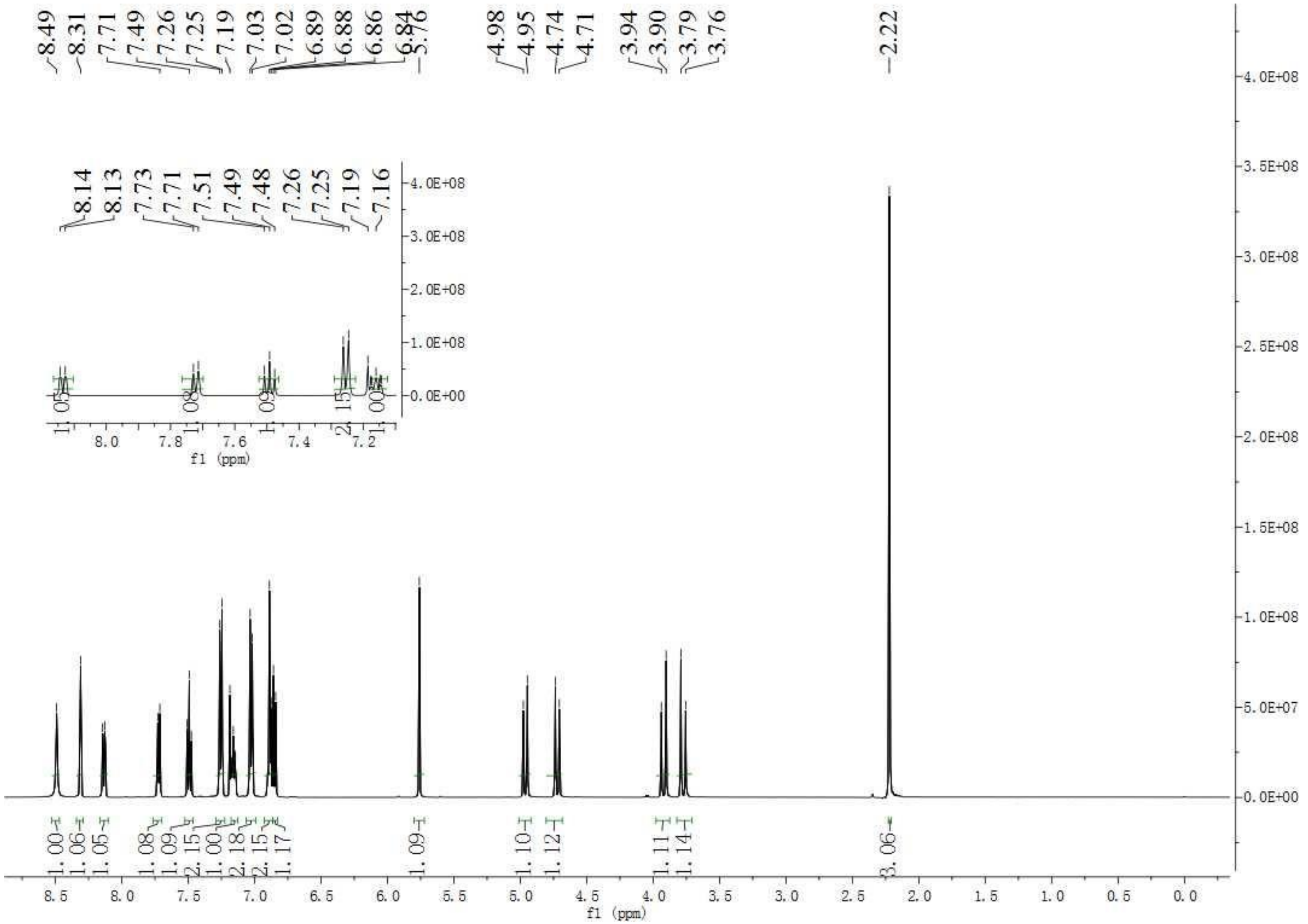

^{1}H NMR of compound **5d**

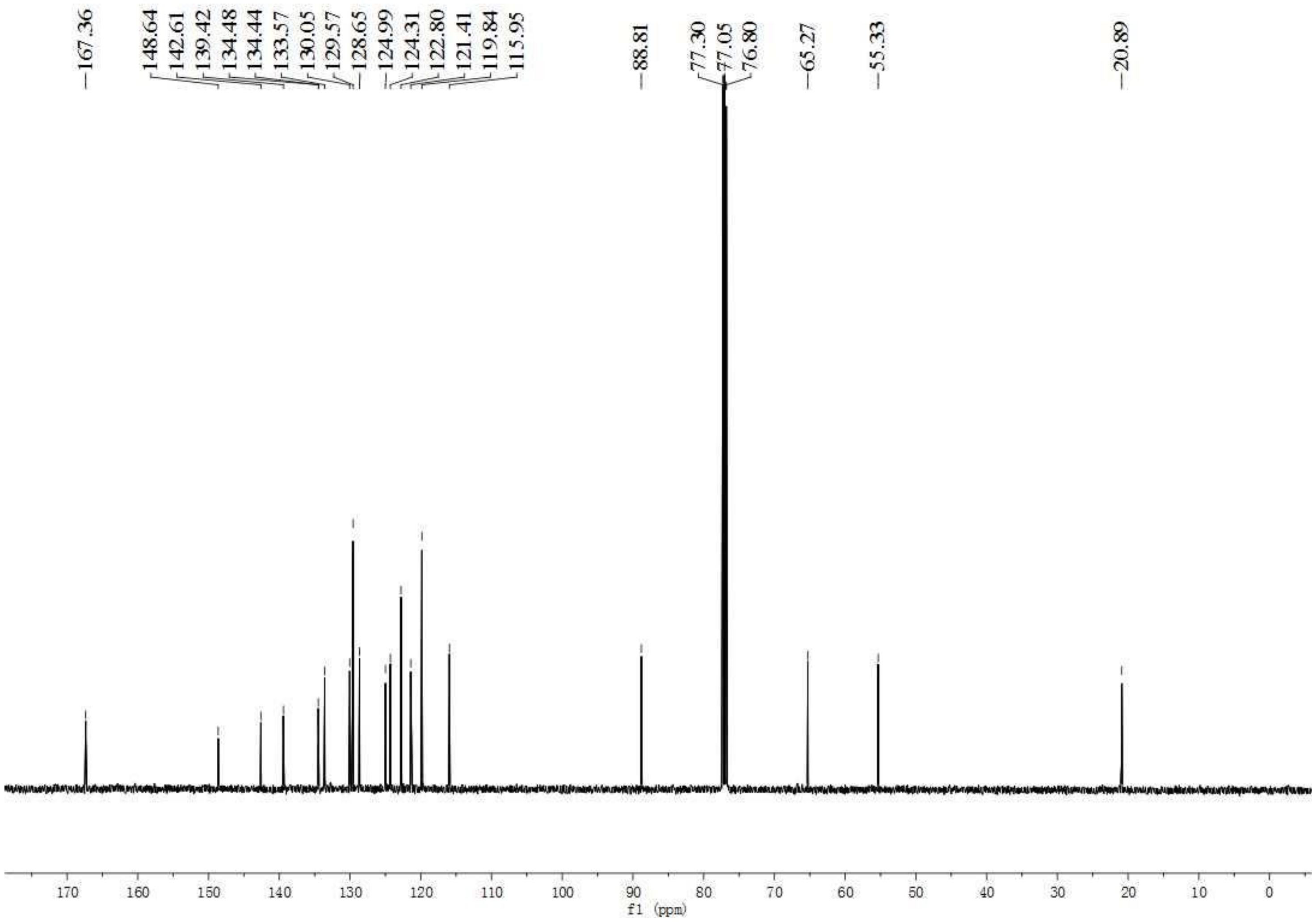

^{13}C NMR of compound **5d**

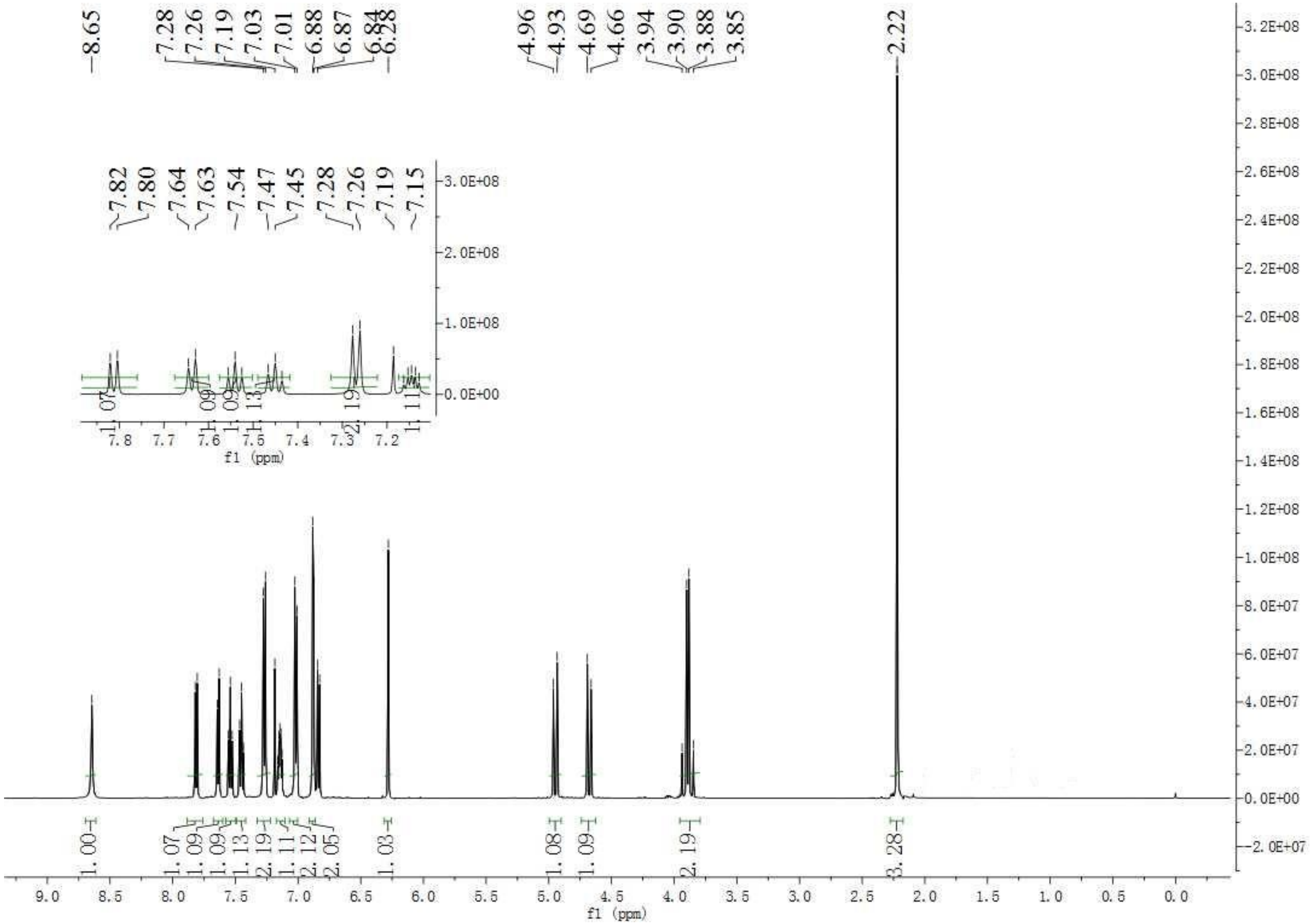

^{1}H NMR of compound **5e**

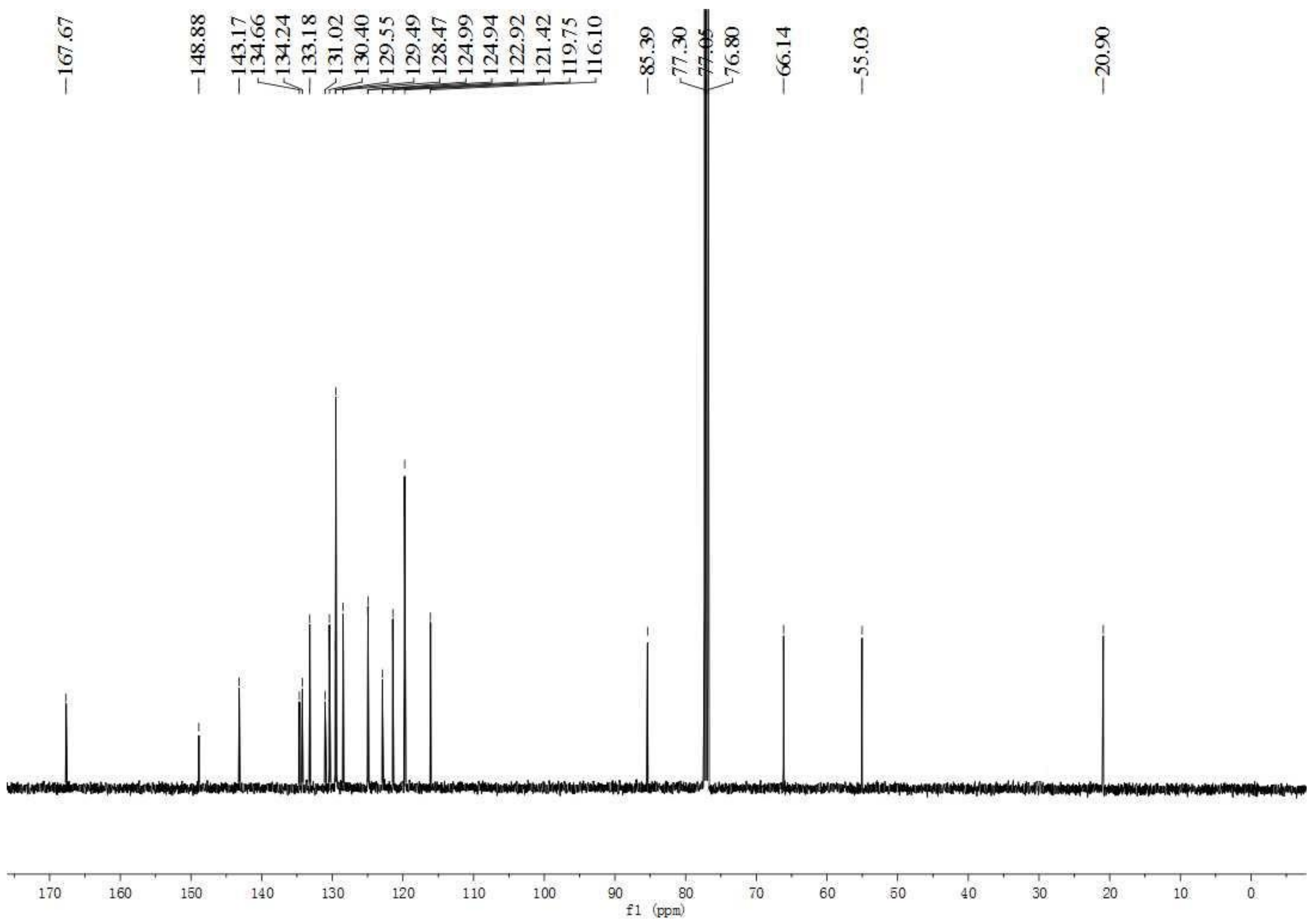

^{13}C NMR of compound **5e**

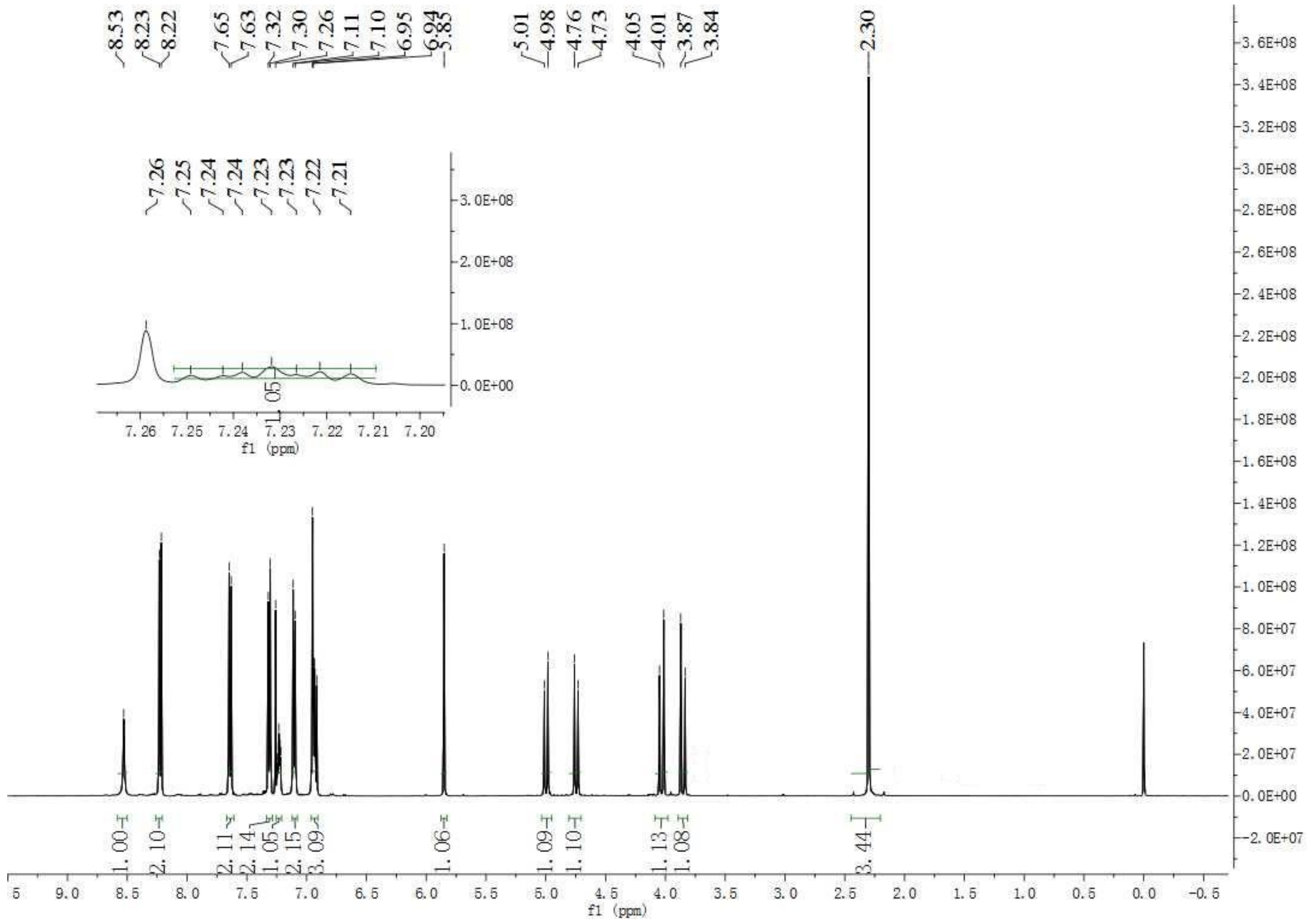

^{1}H NMR of compound **5f**

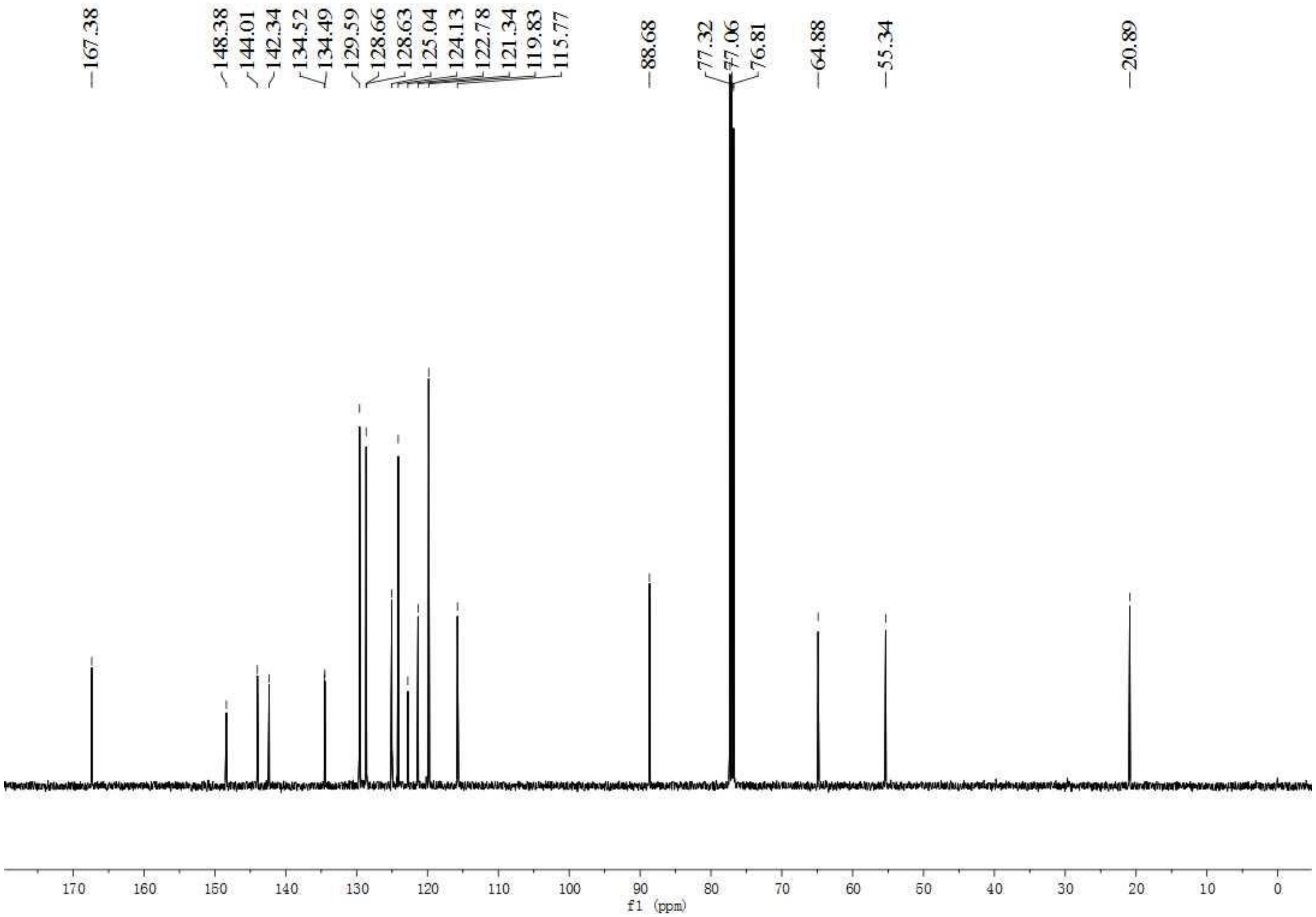

^{13}C NMR of compound **5f**

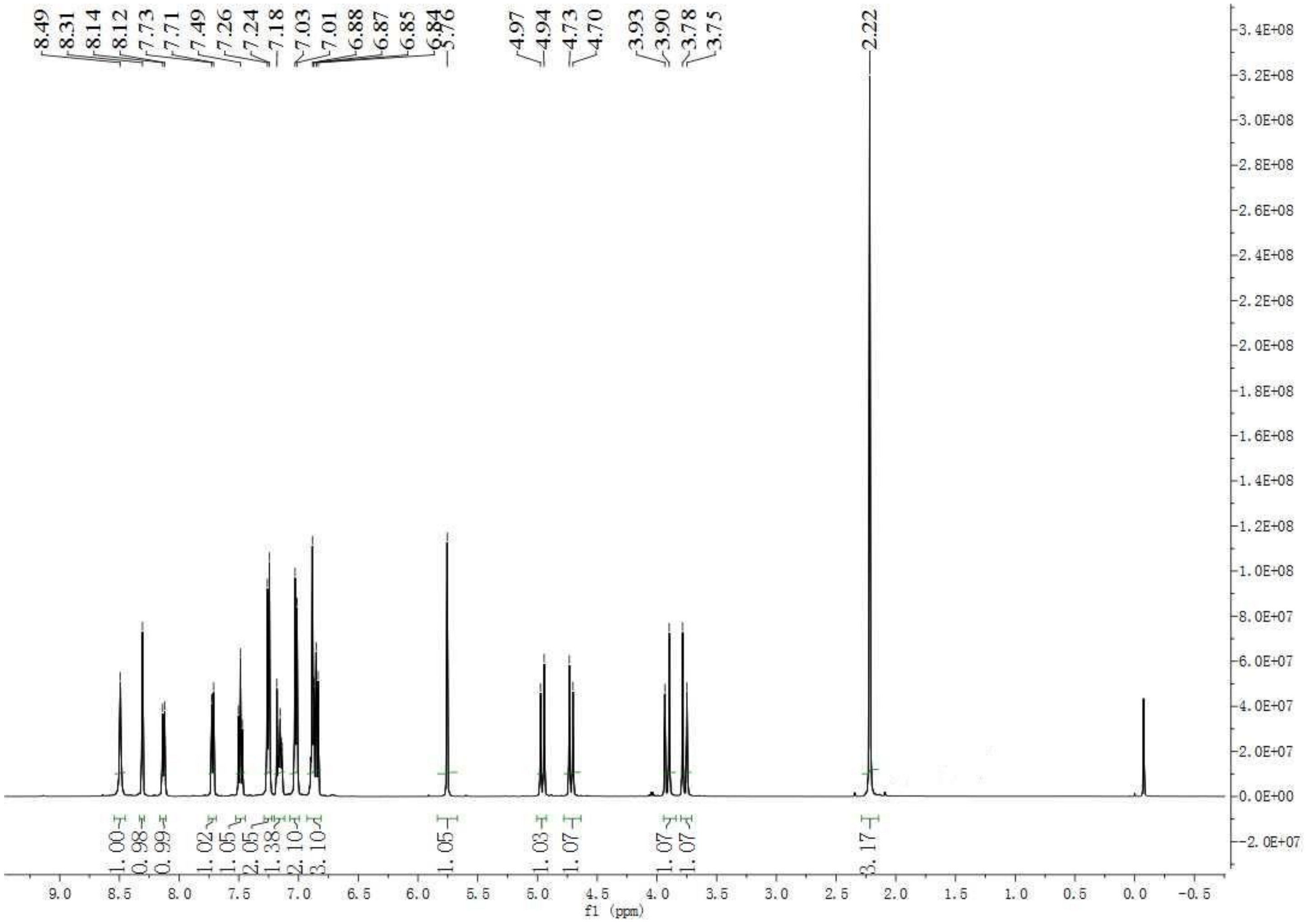

^{1}H NMR of compound **5g**

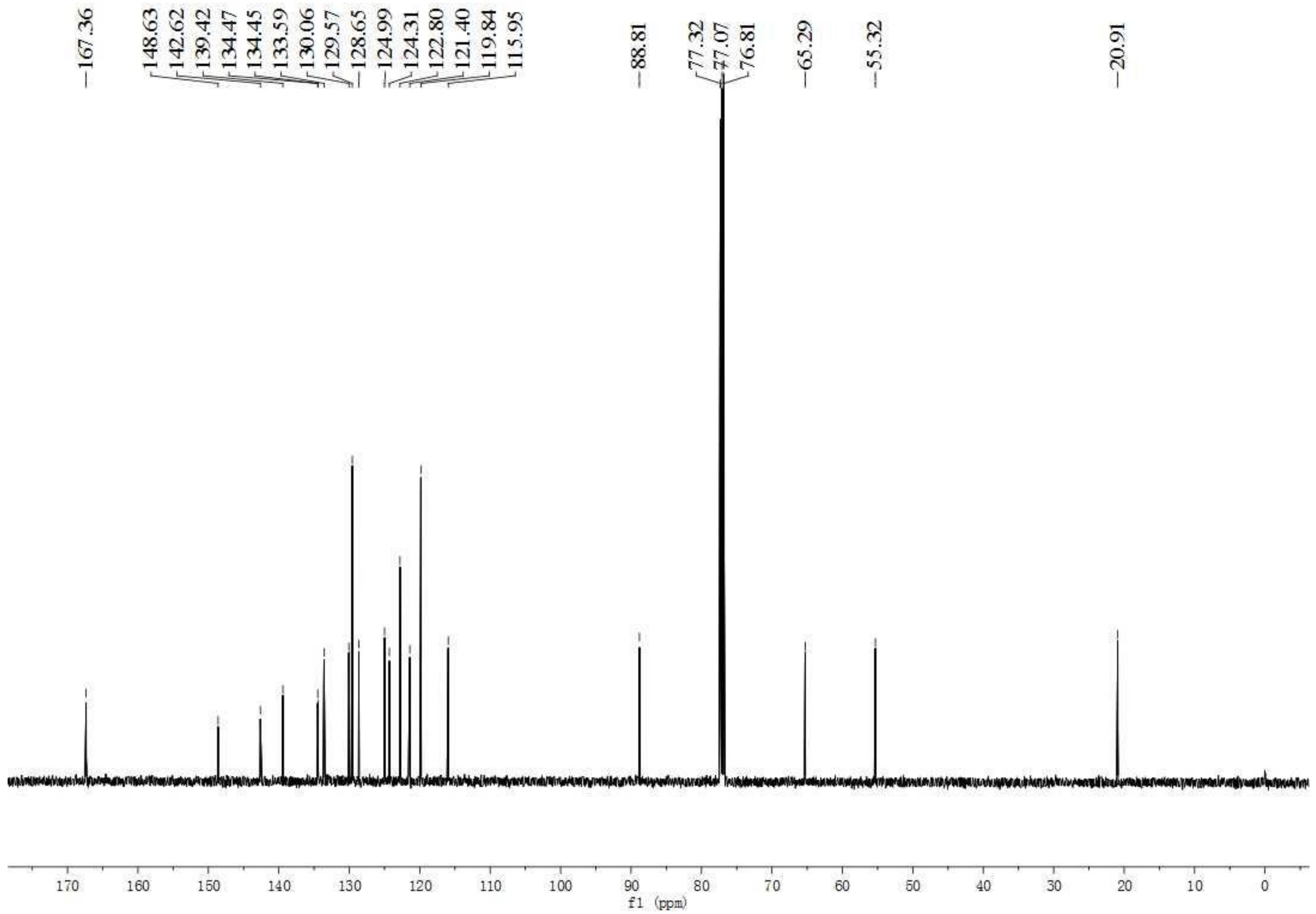

^{13}C NMR of compound **5g**

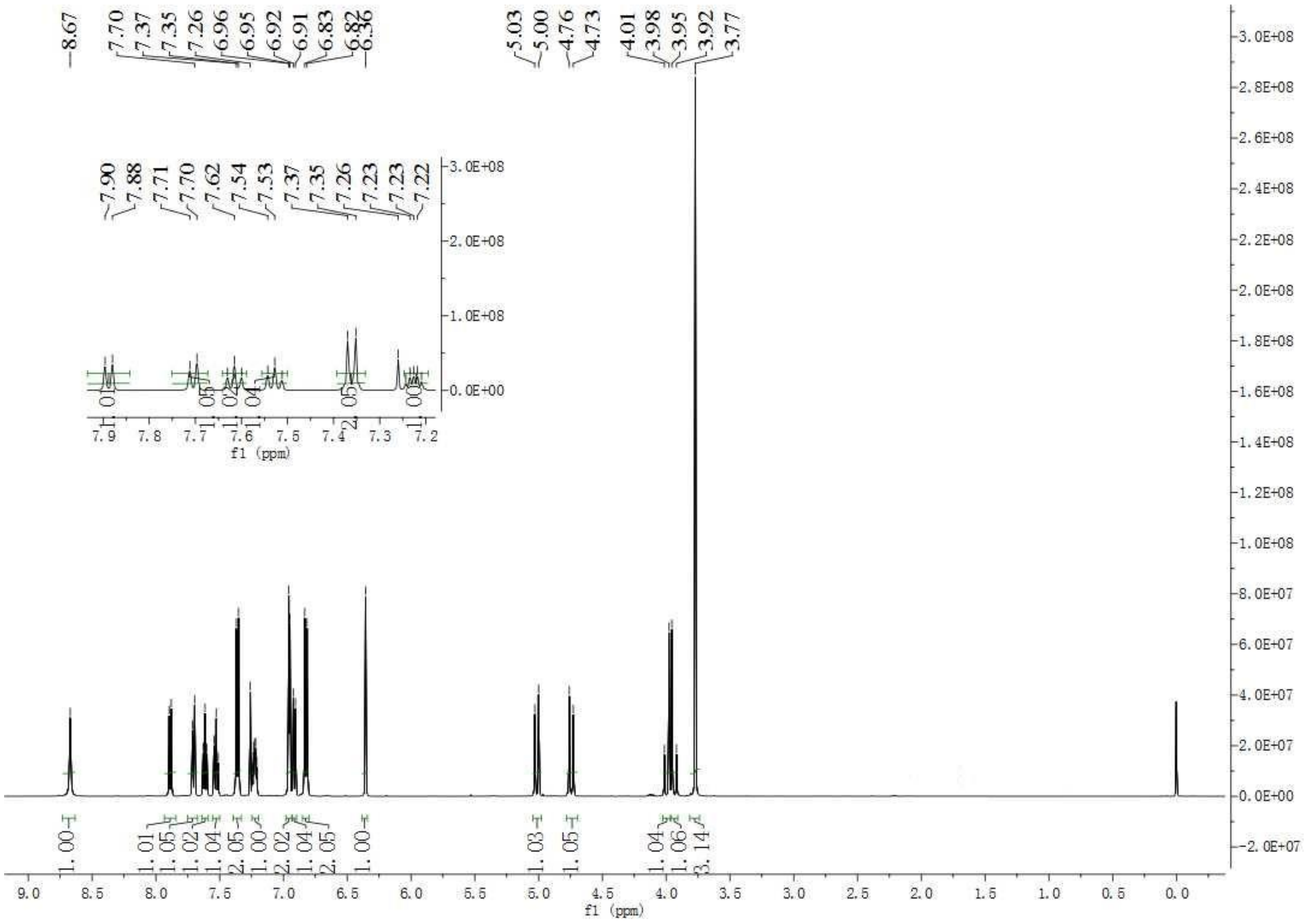

^{1}H NMR of compound **5h**

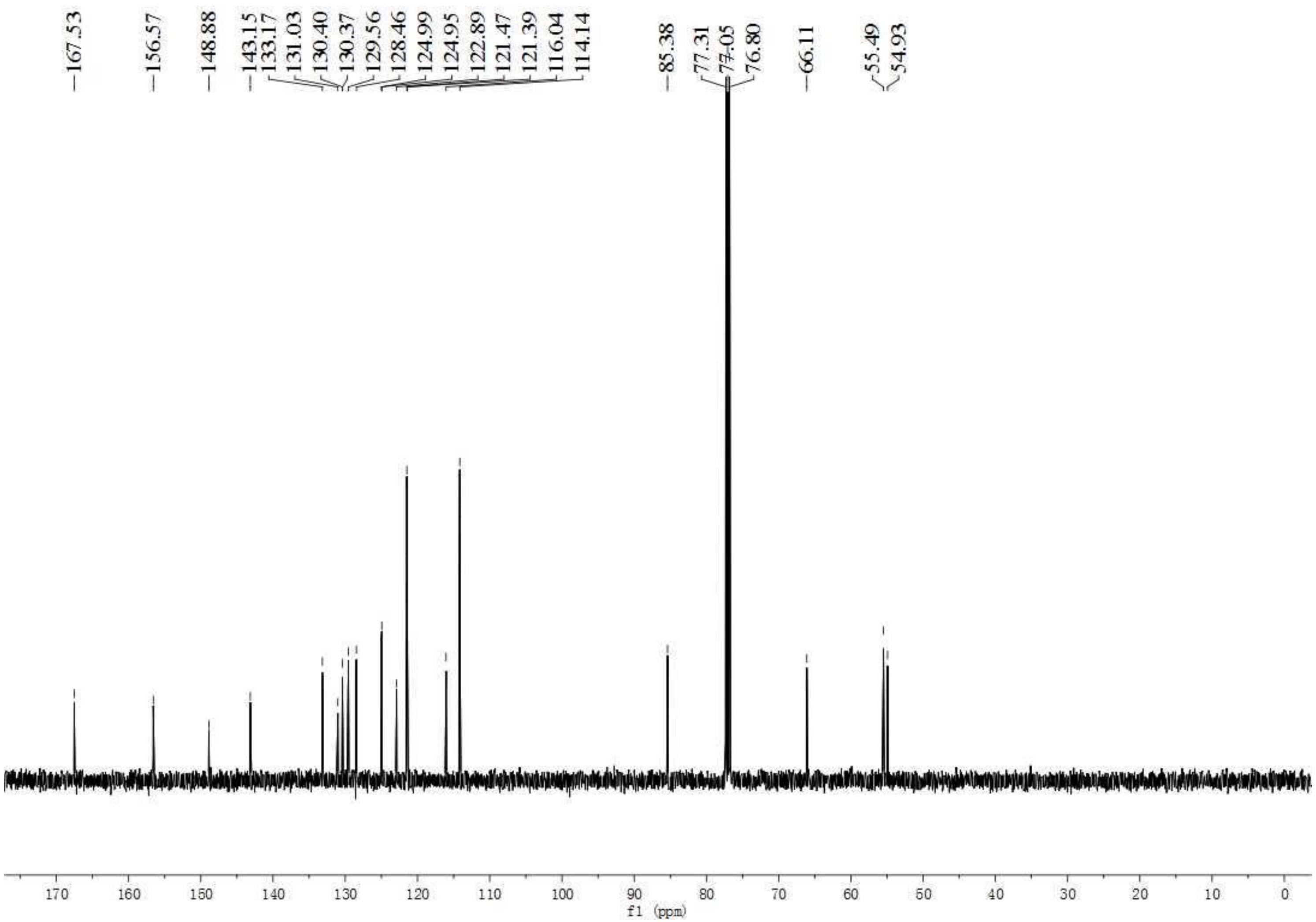

^{13}C NMR of compound **5h**

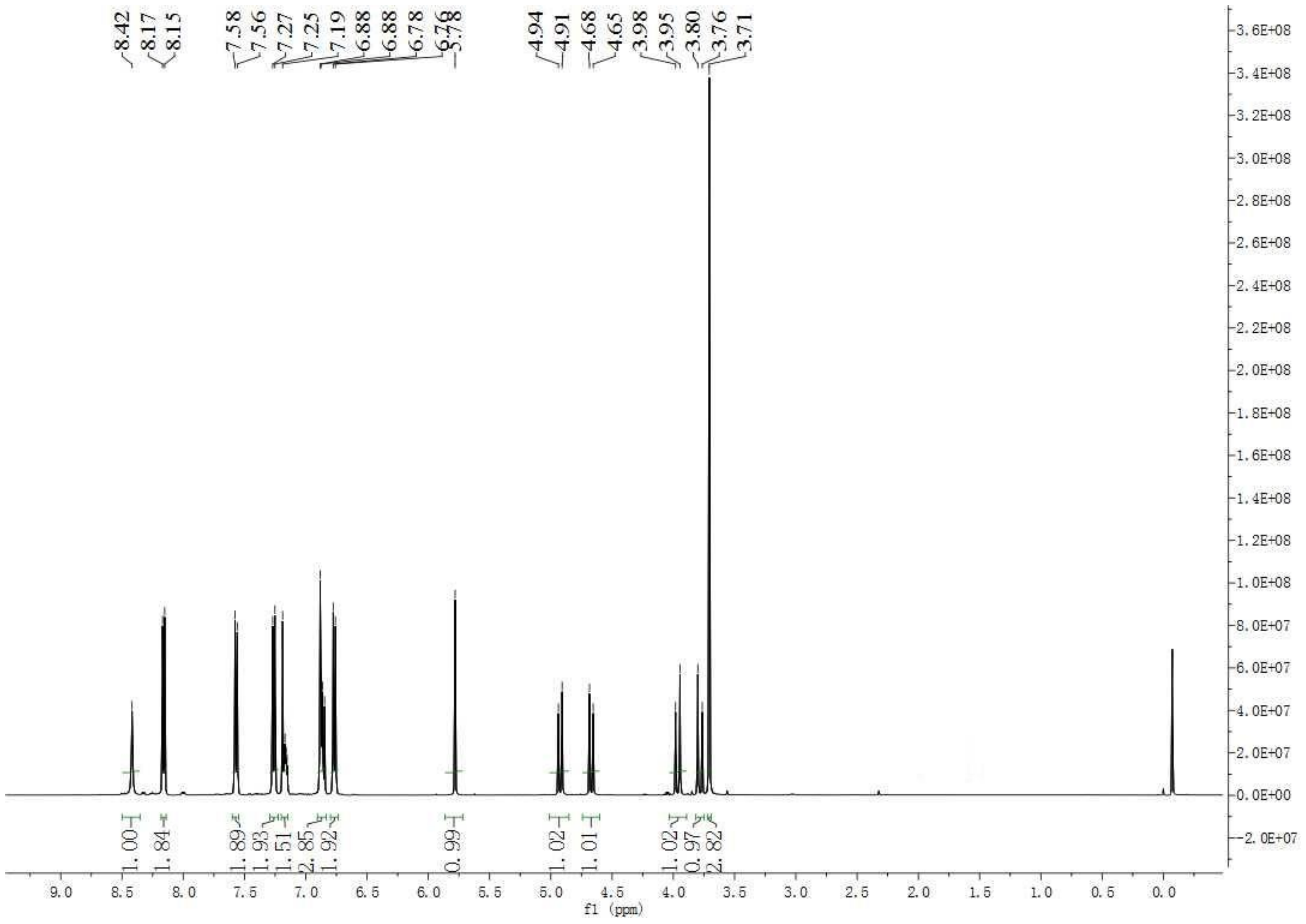

^{1}H NMR of compound **5i**

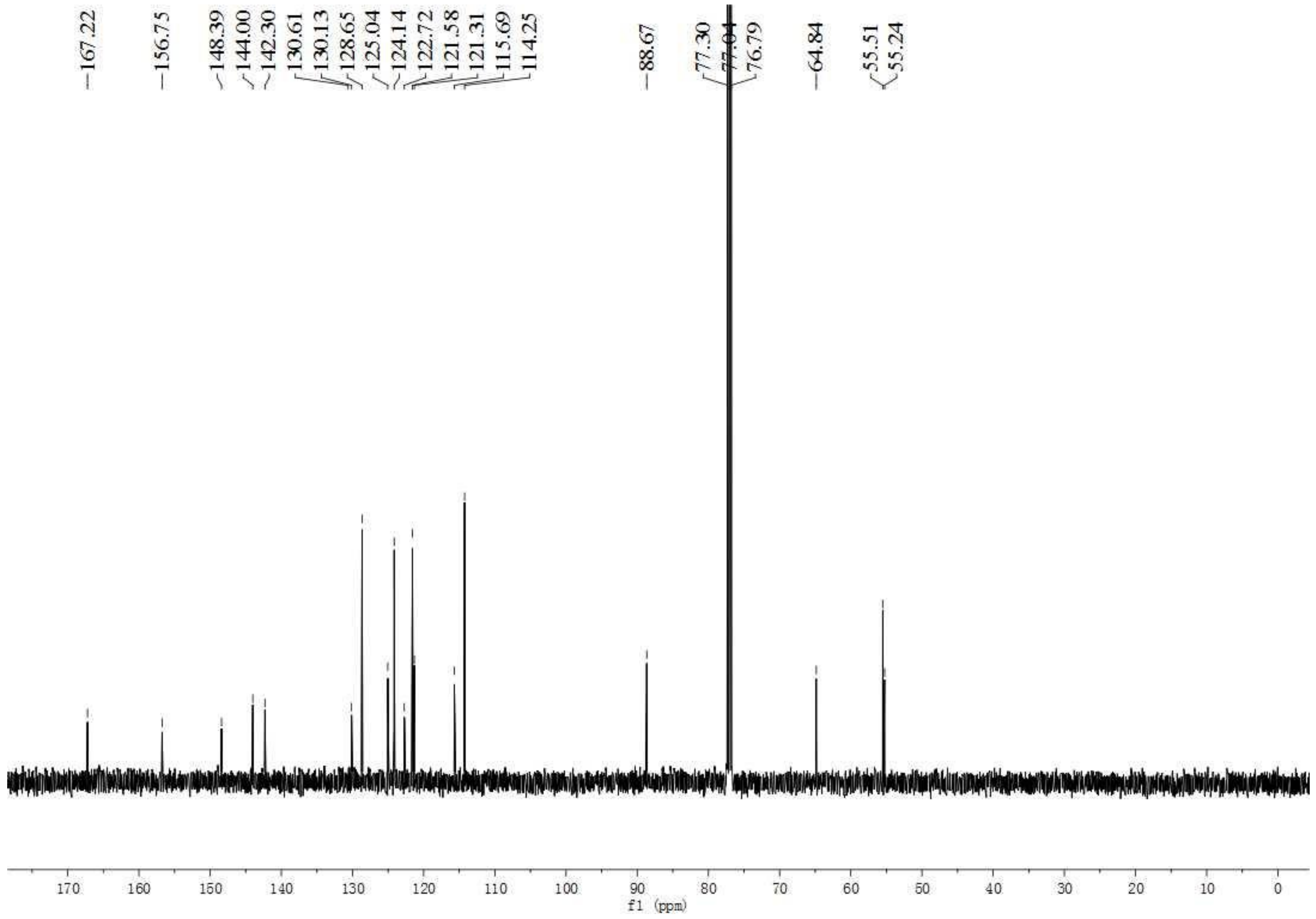

^{13}C NMR of compound **5i**

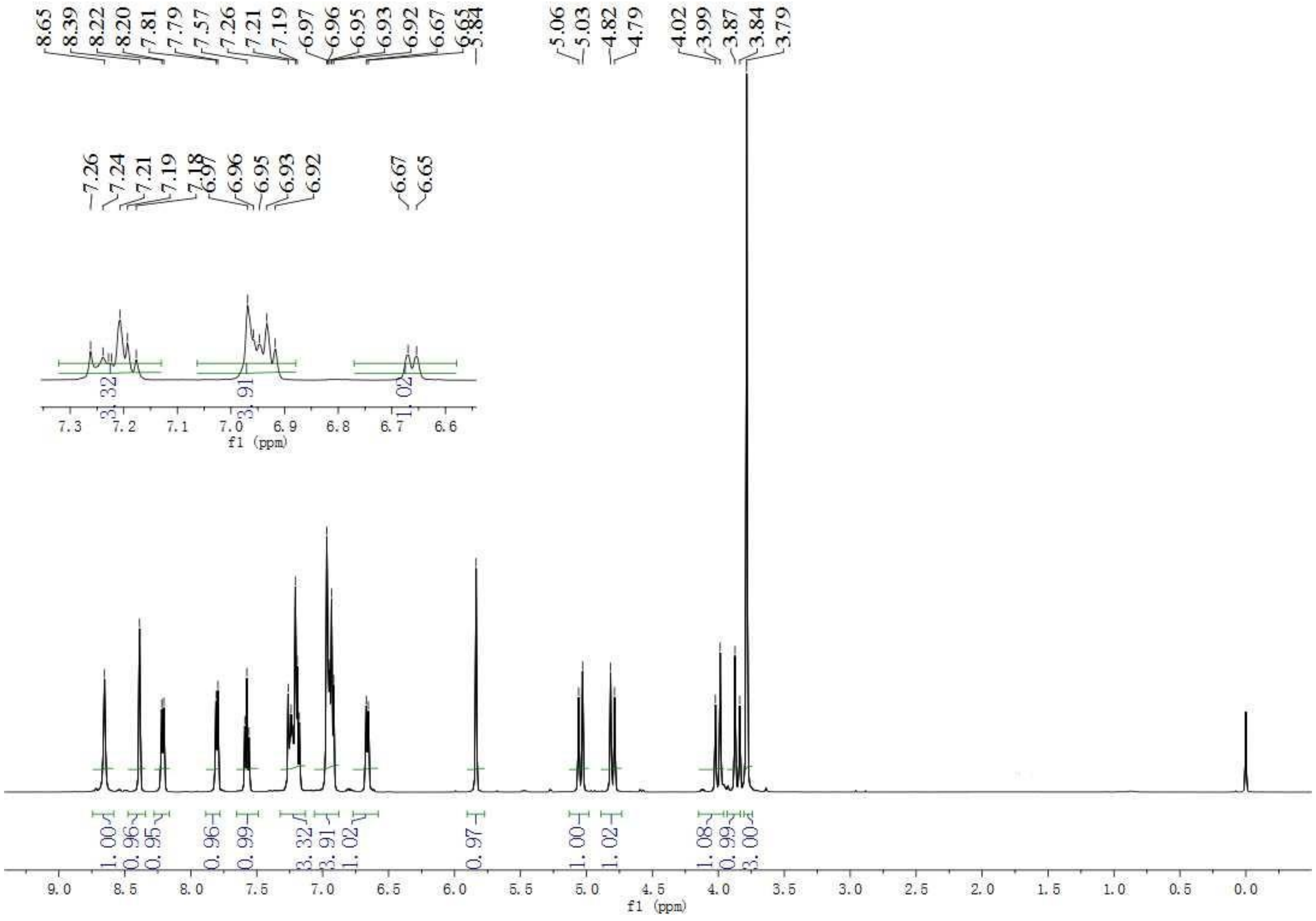

^{1}H NMR of compound **5j**

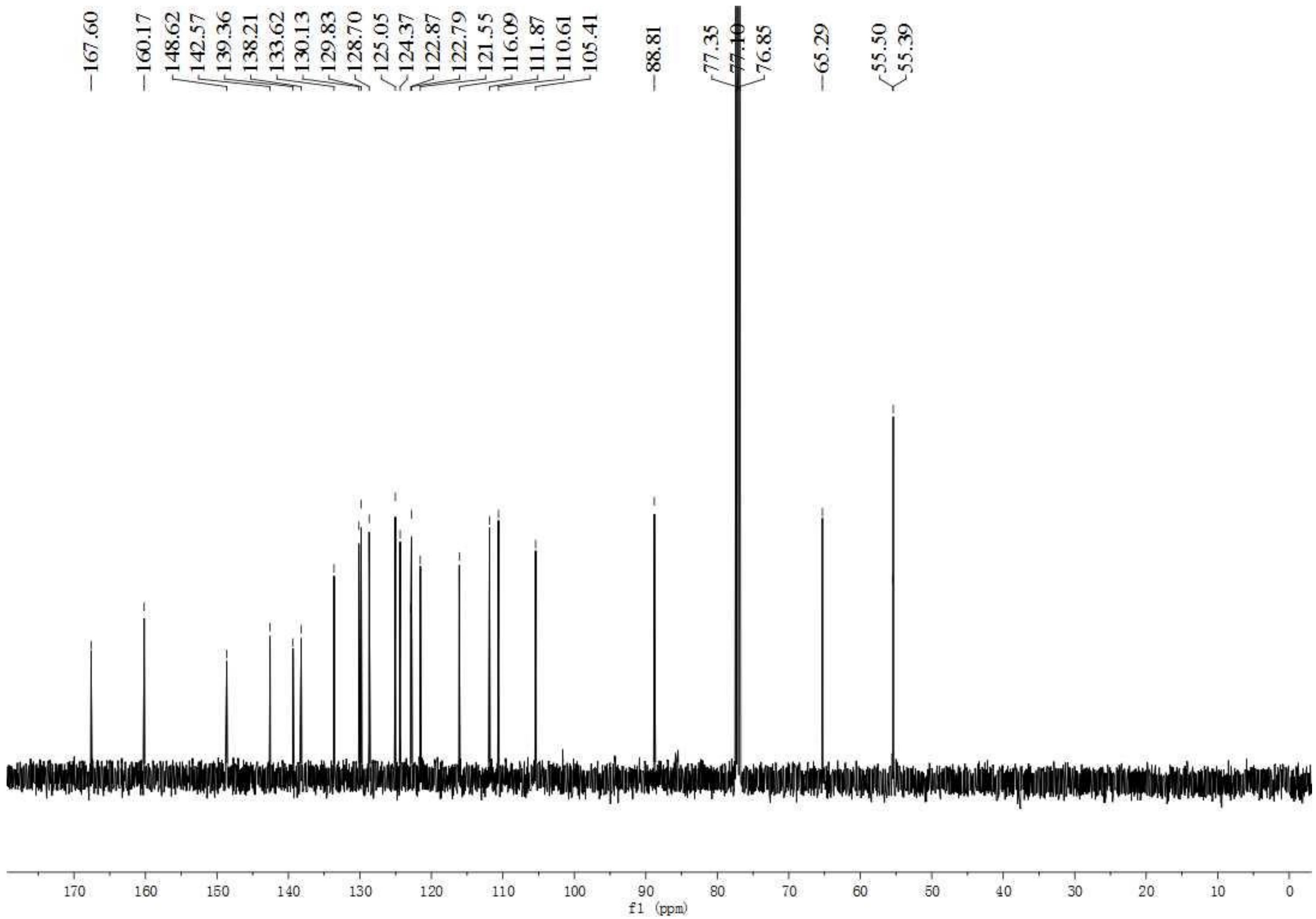

^{13}C NMR of compound **5j**

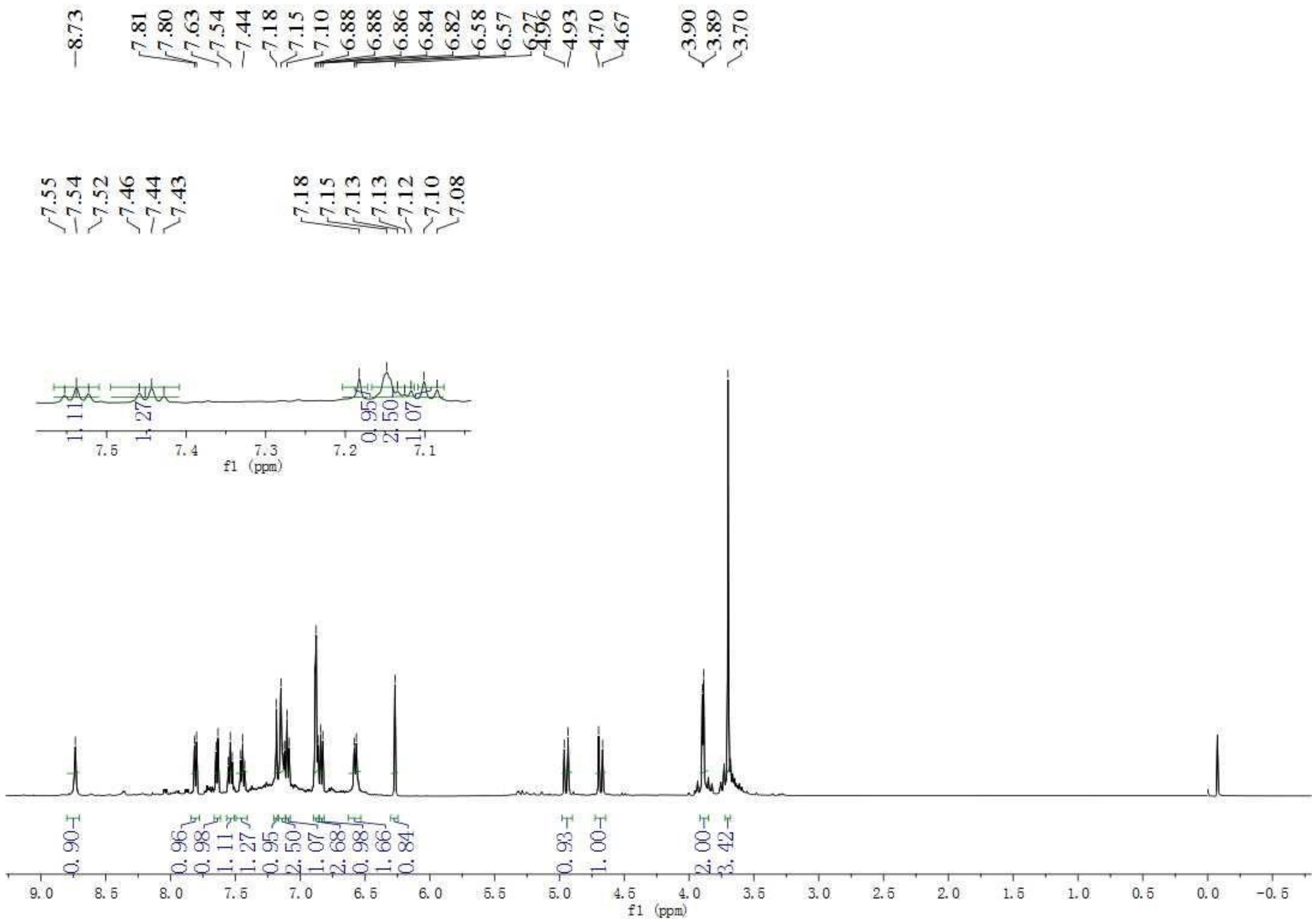

^{1}H NMR of compound **5k**

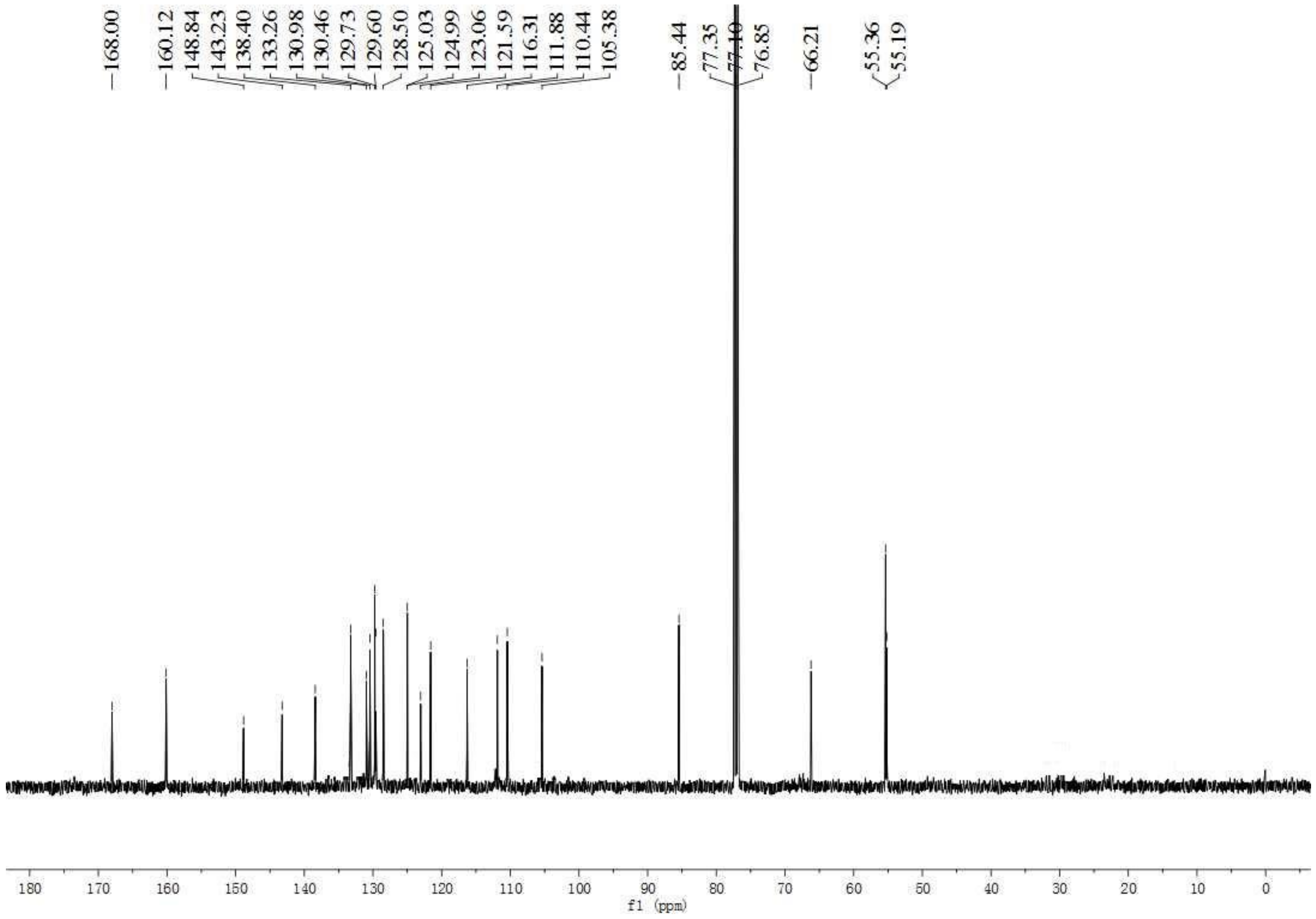

^{13}C NMR of compound **5k**

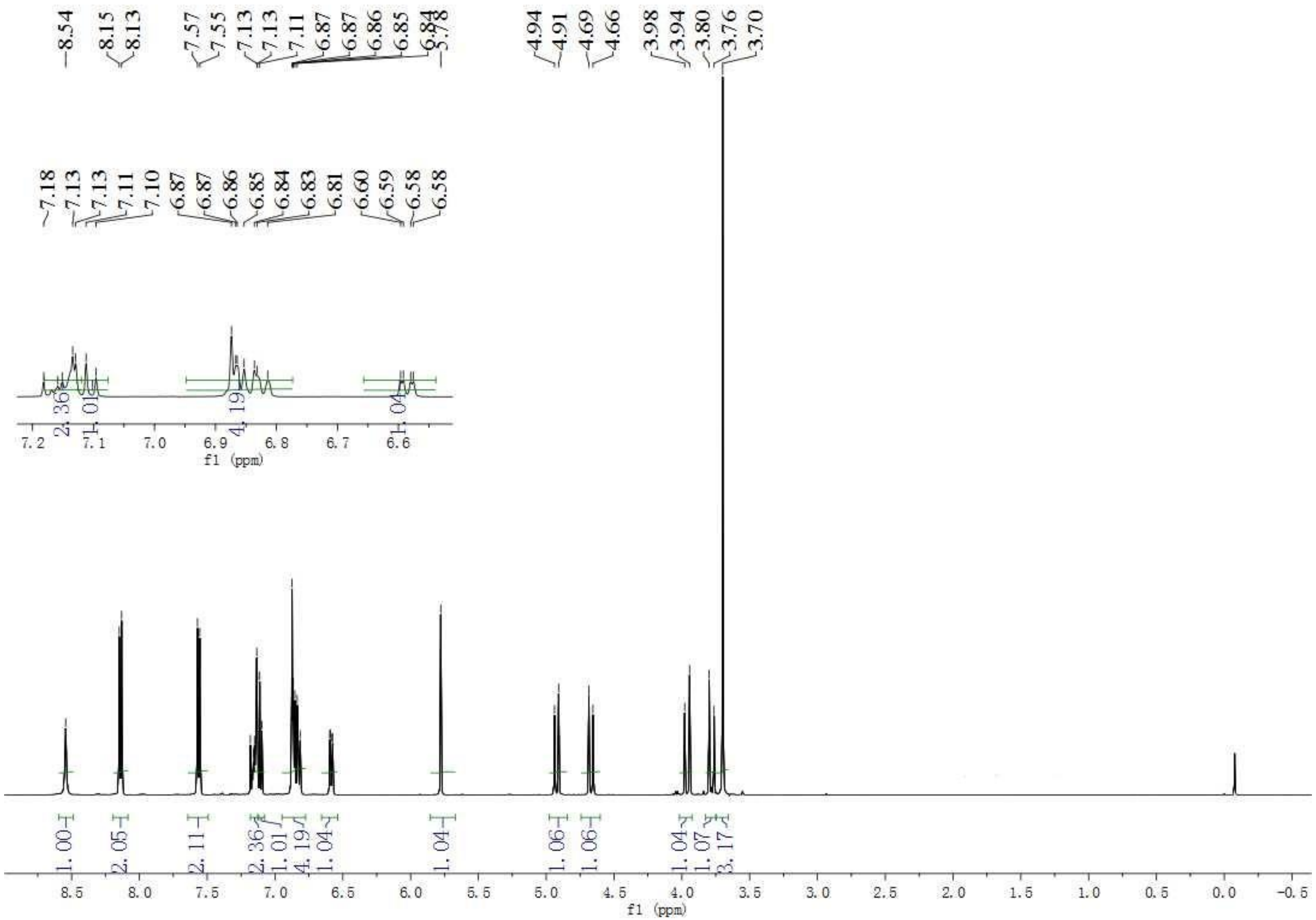

^{1}H NMR of compound **5l**

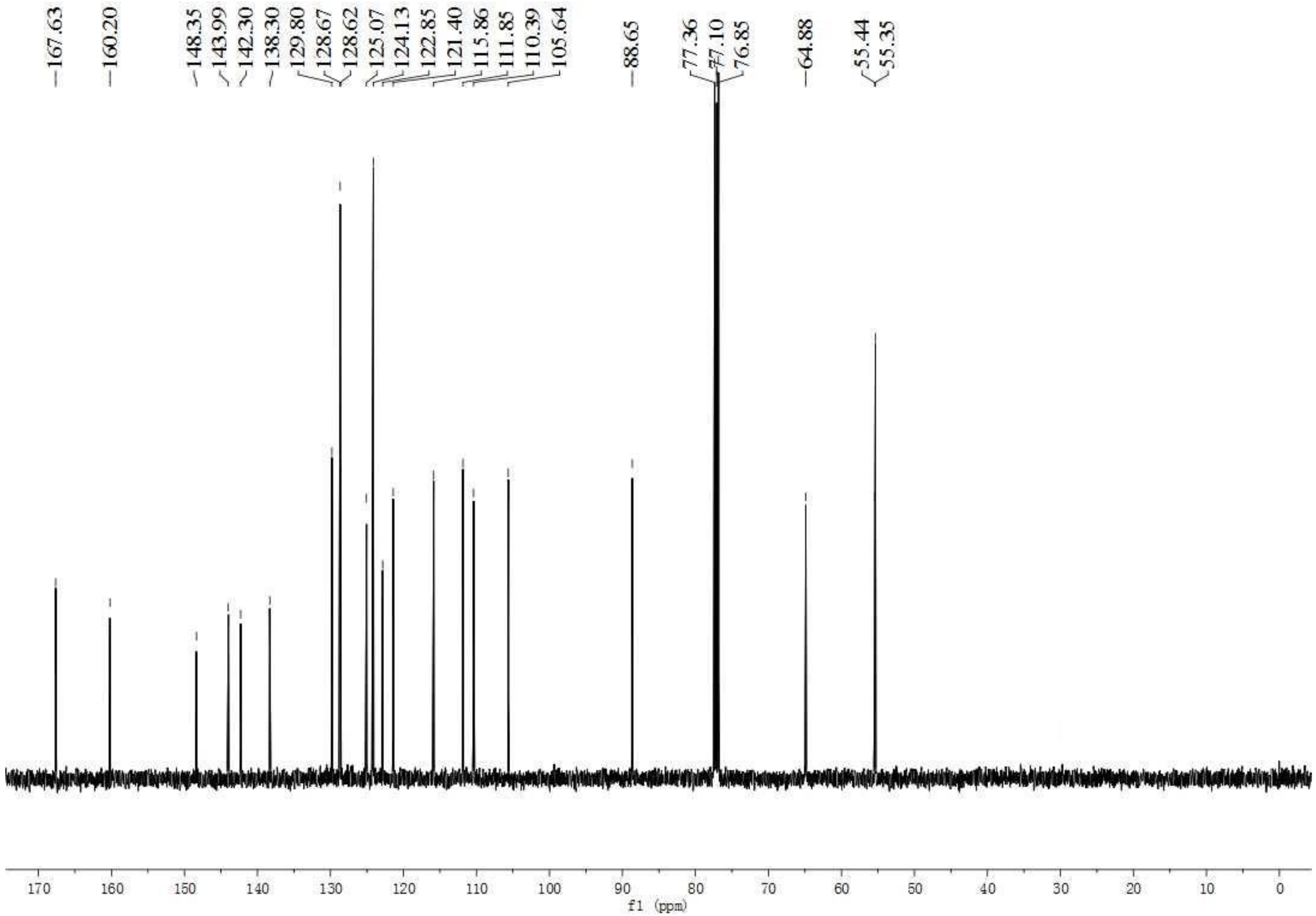

^{13}C NMR of compound **5l**

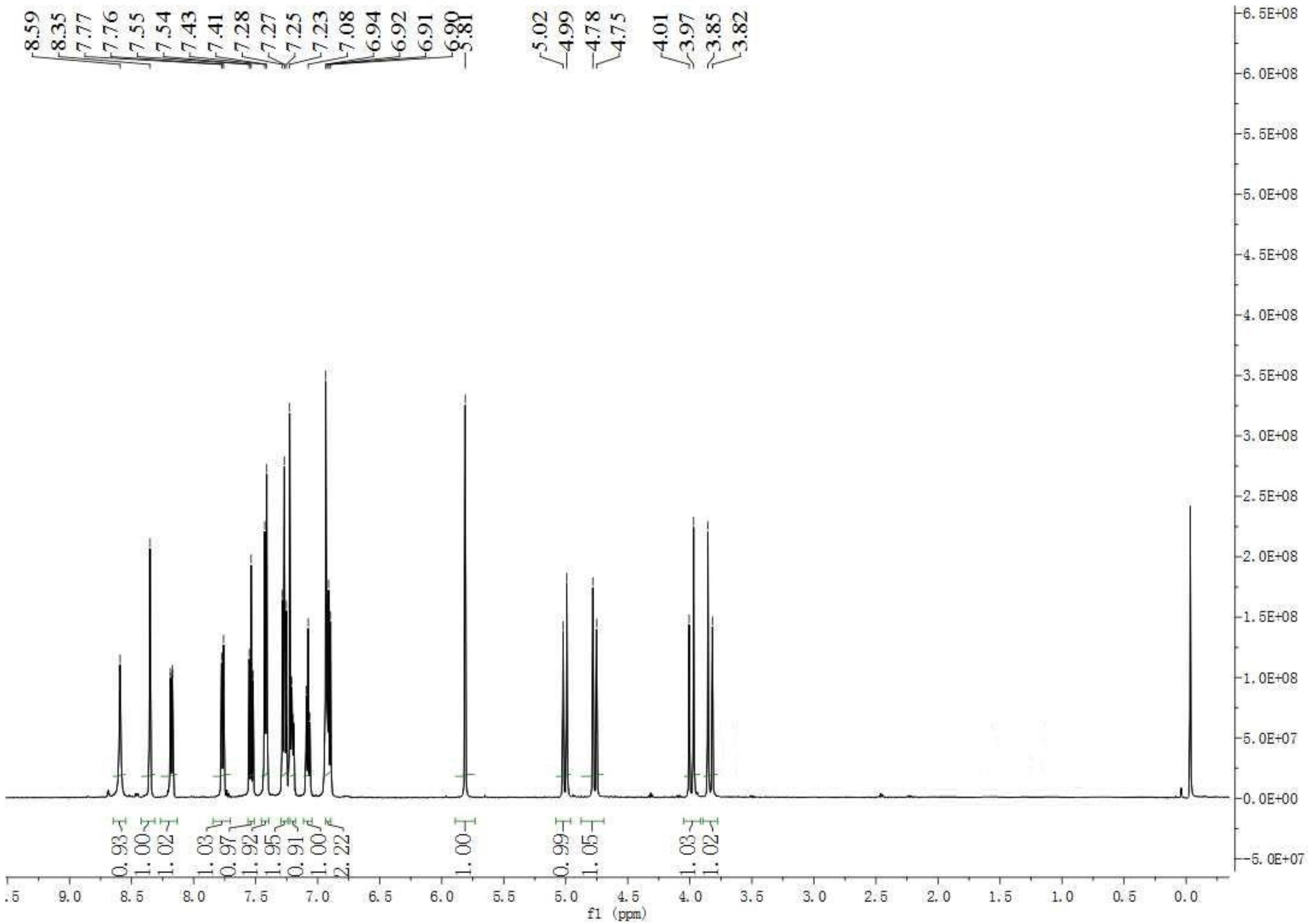

^{1}H NMR of compound **5m**

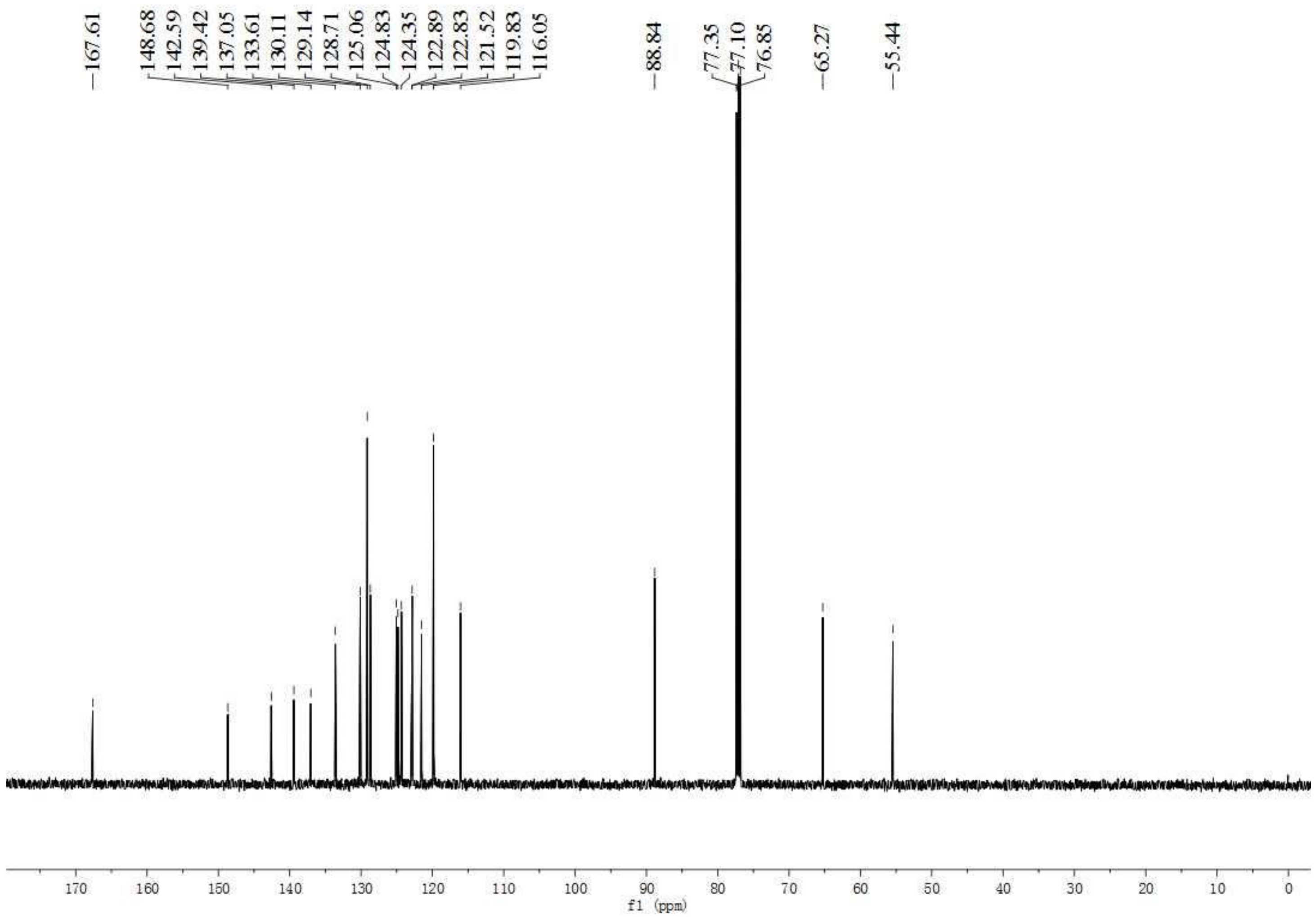

^{13}C NMR of compound **5m**

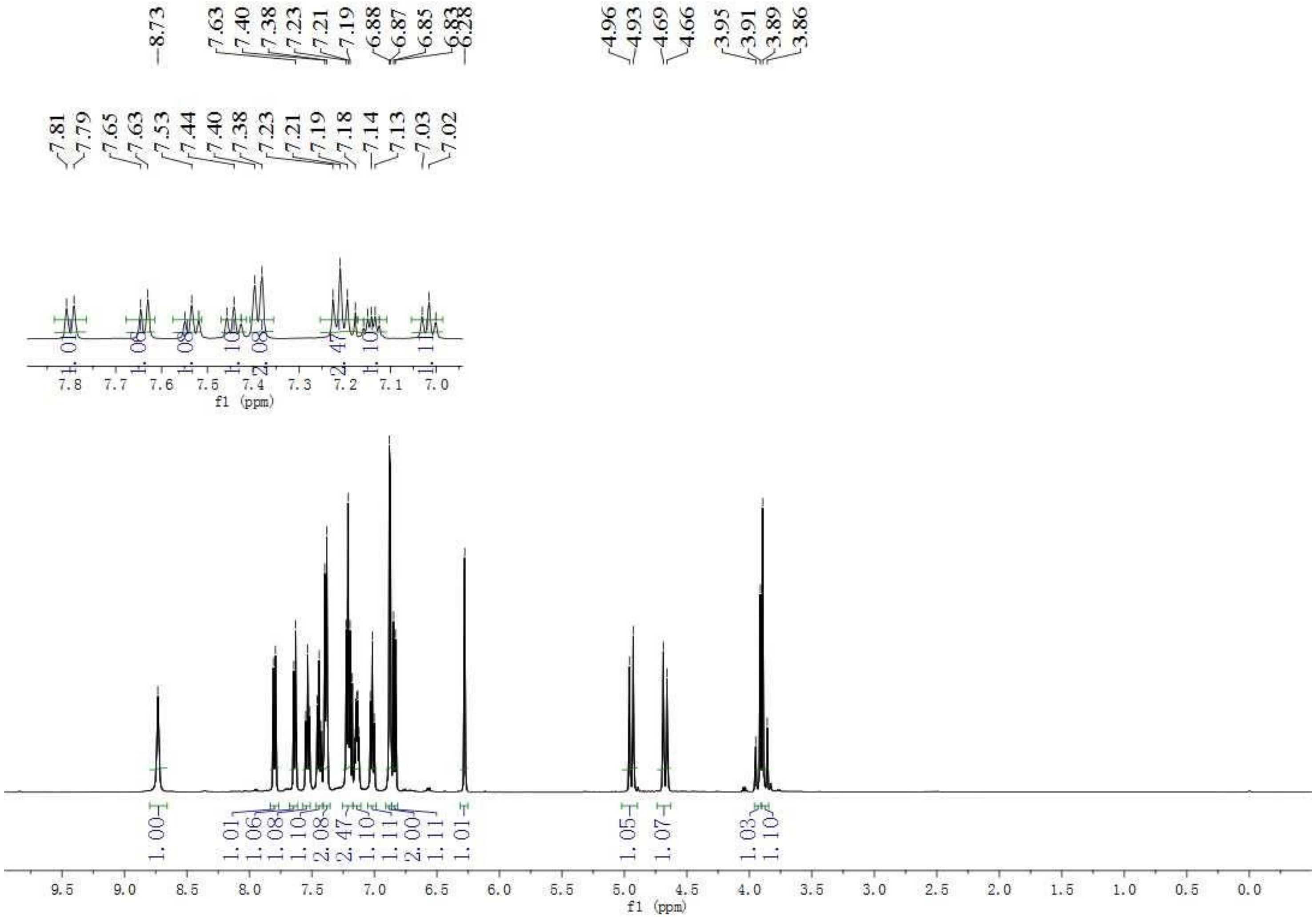

^{1}H NMR of compound **5n**

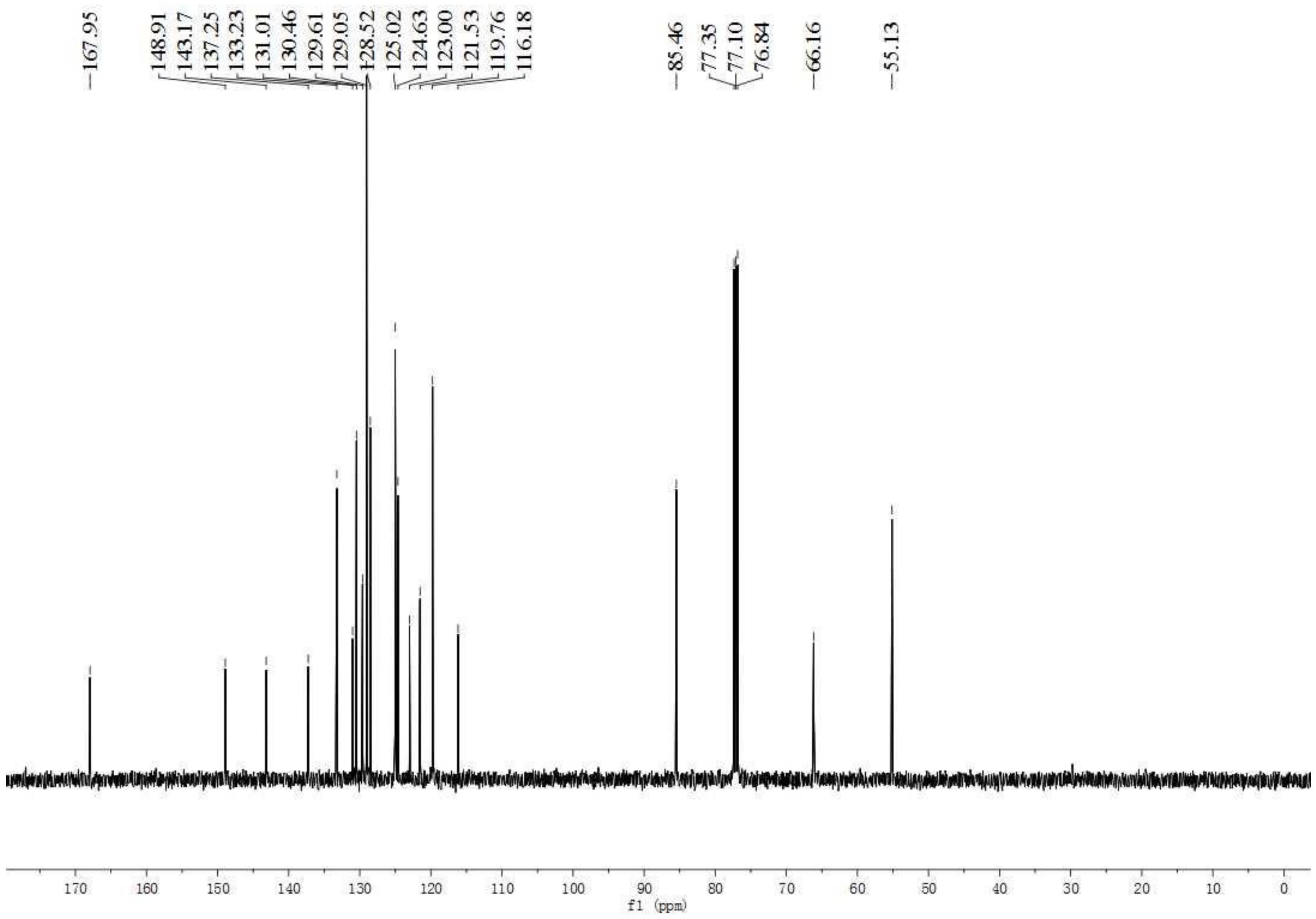

^{13}C NMR of compound **5n**

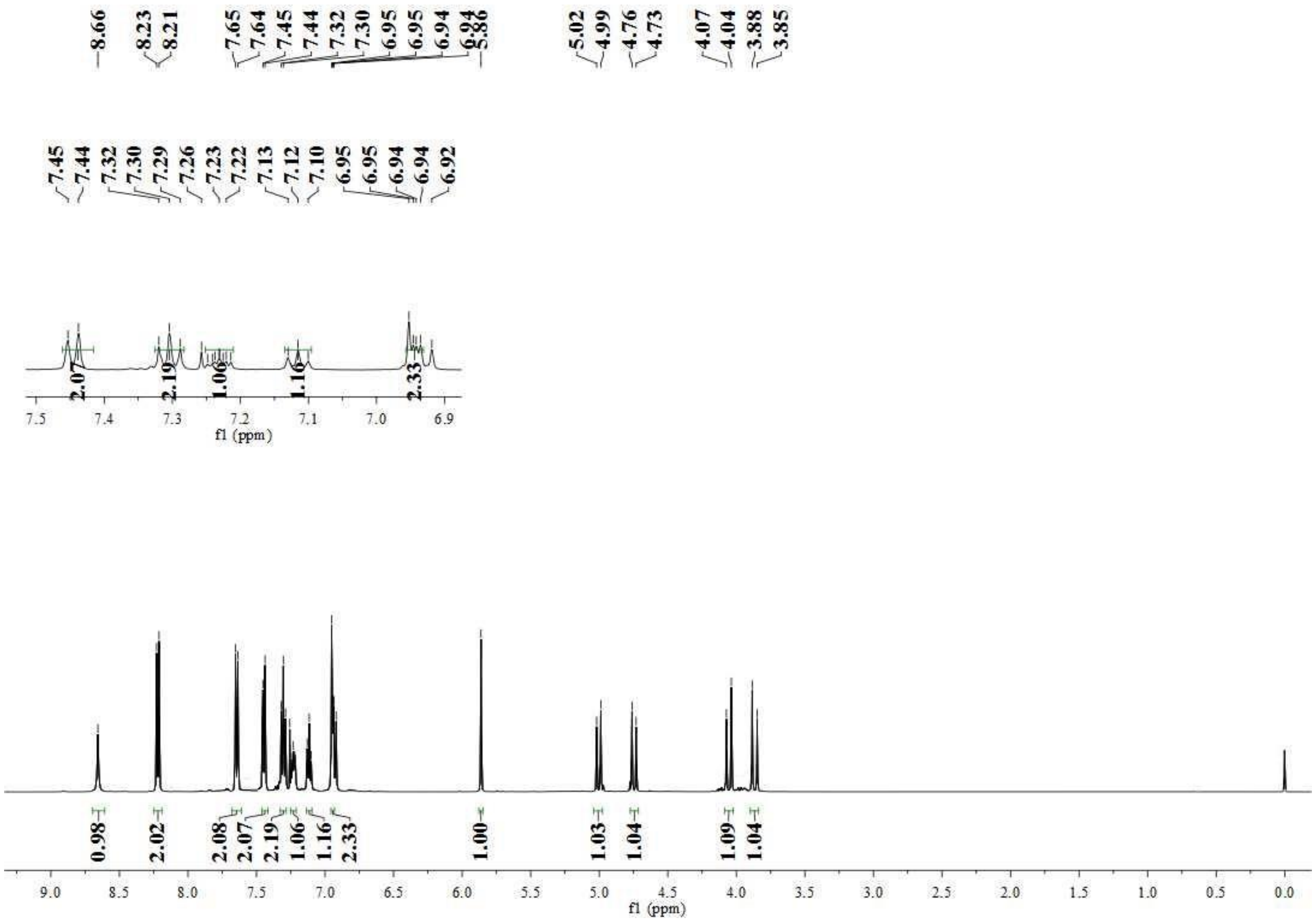

^{1}H NMR of compound **5o**

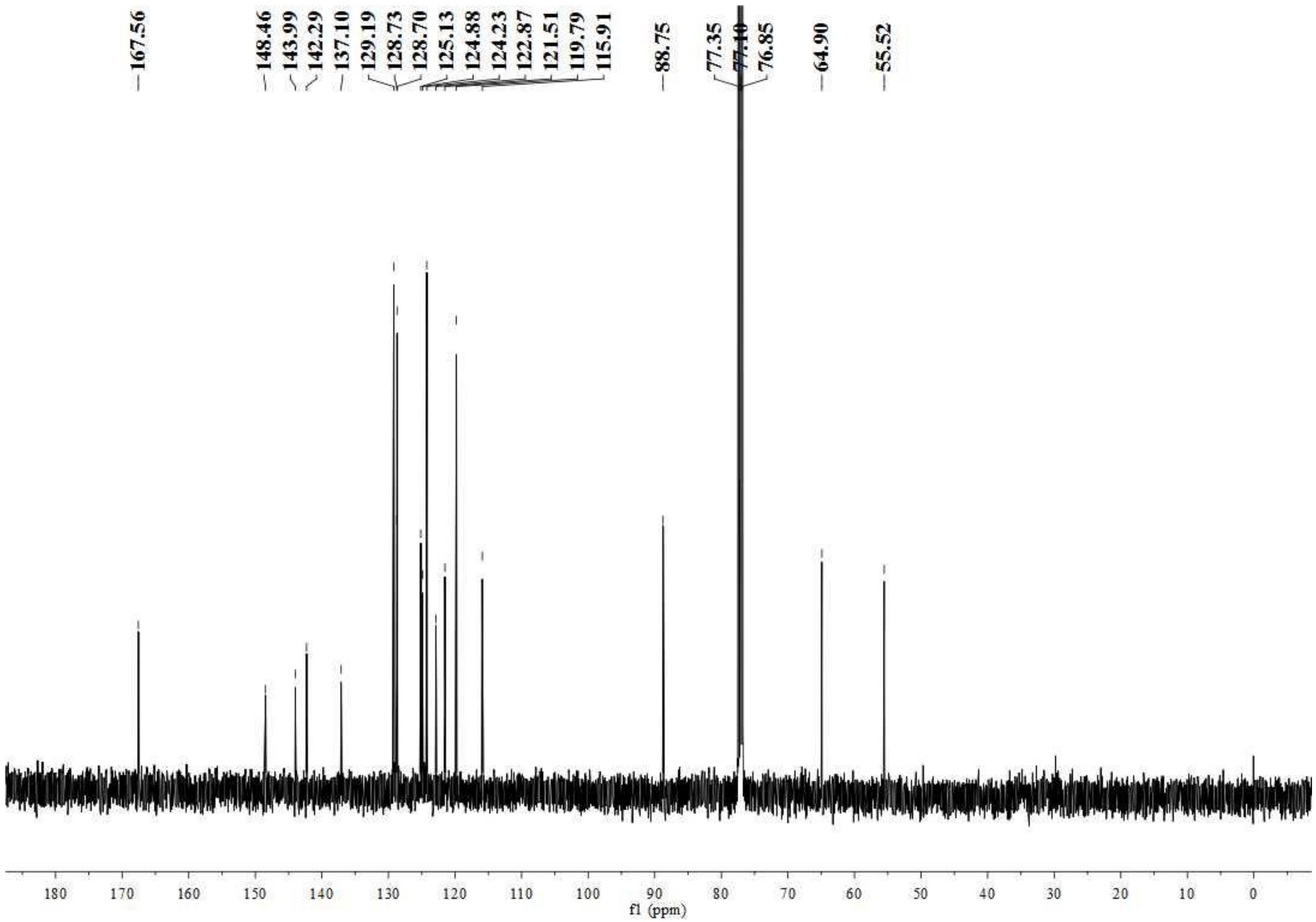

^{13}C NMR of compound **5o**

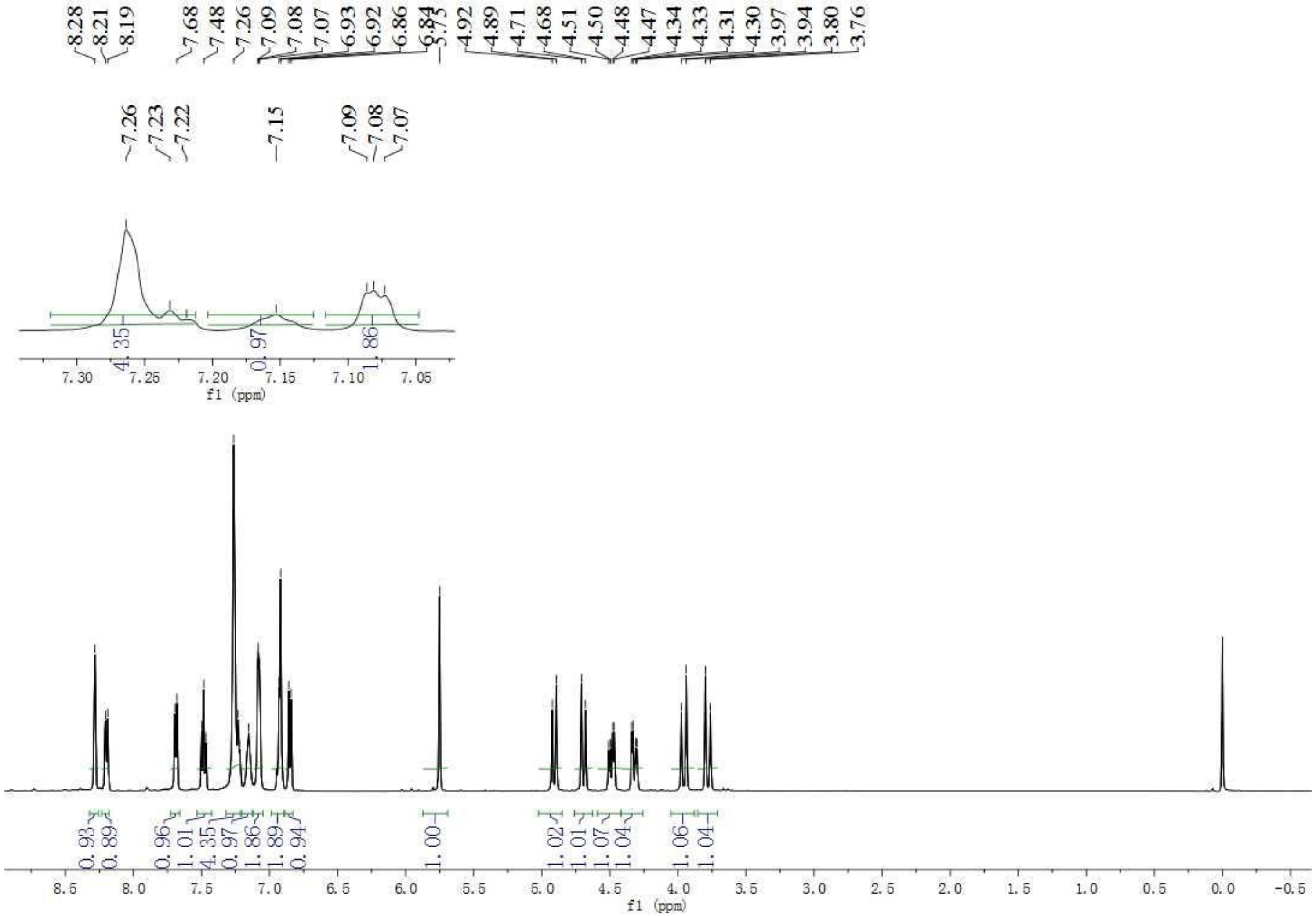

^{1}H NMR of compound **5p**

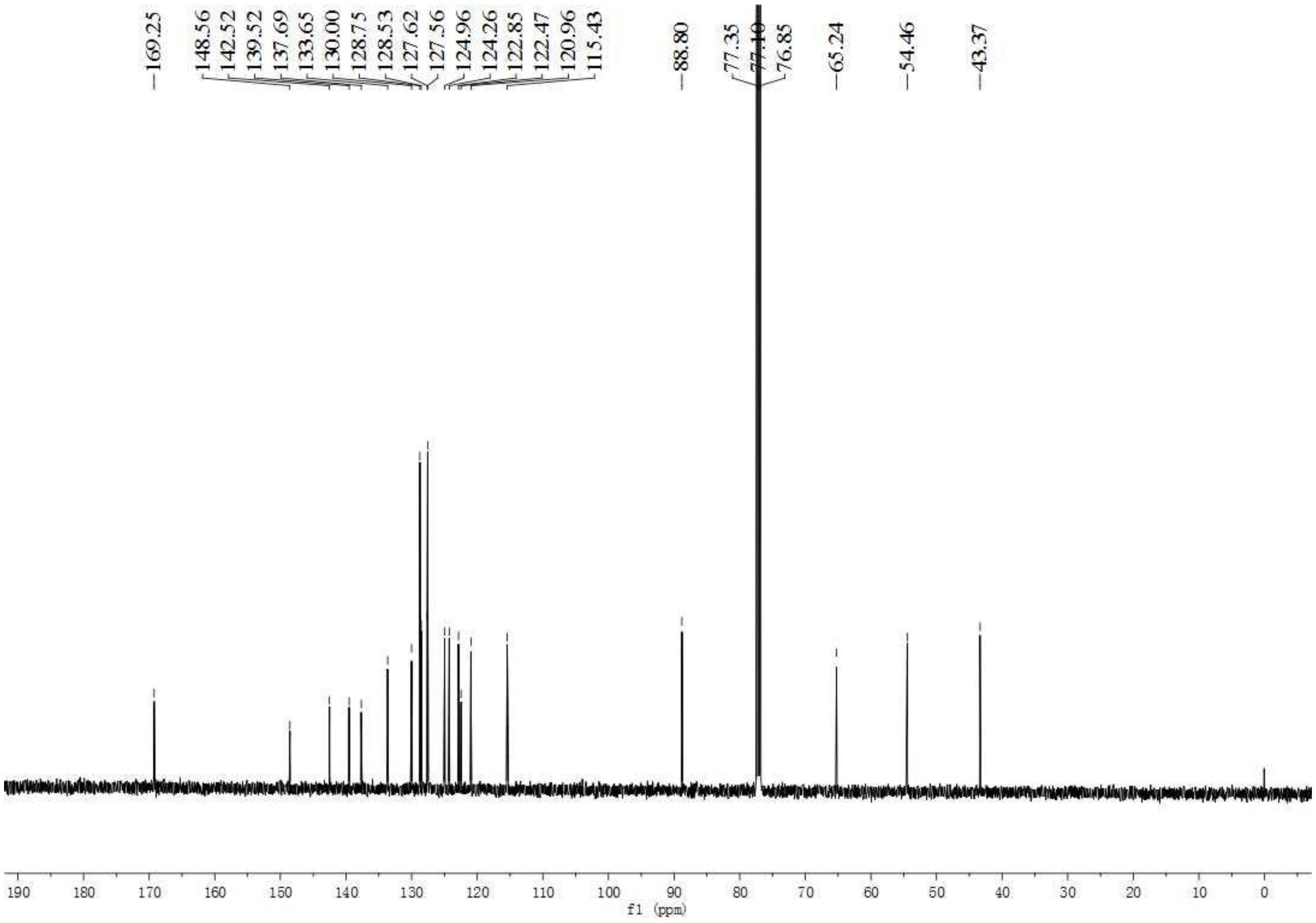

^{13}C NMR of compound **5p**

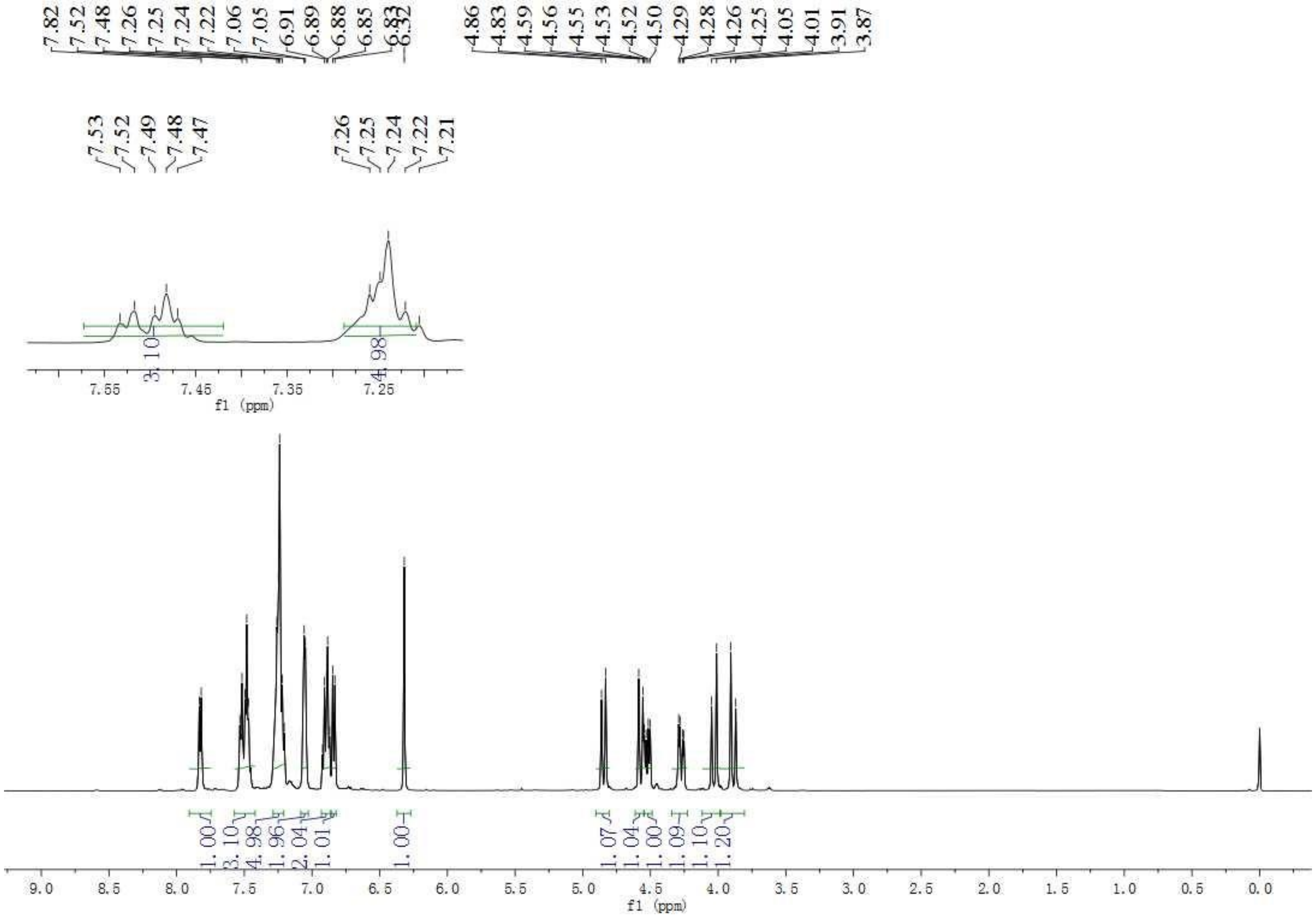

^{1}H NMR of compound **5q**

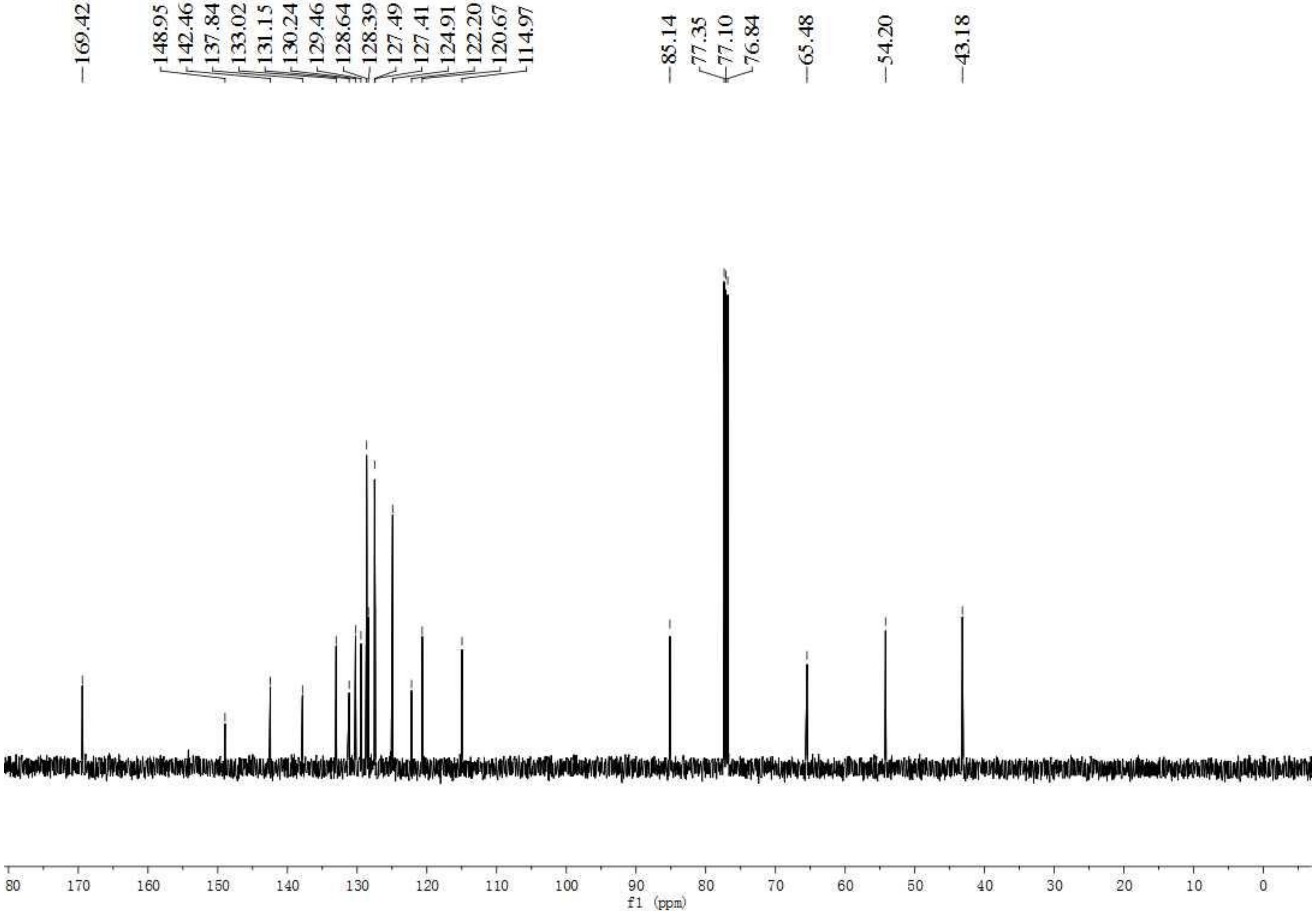

^{13}C NMR of compound **5q**

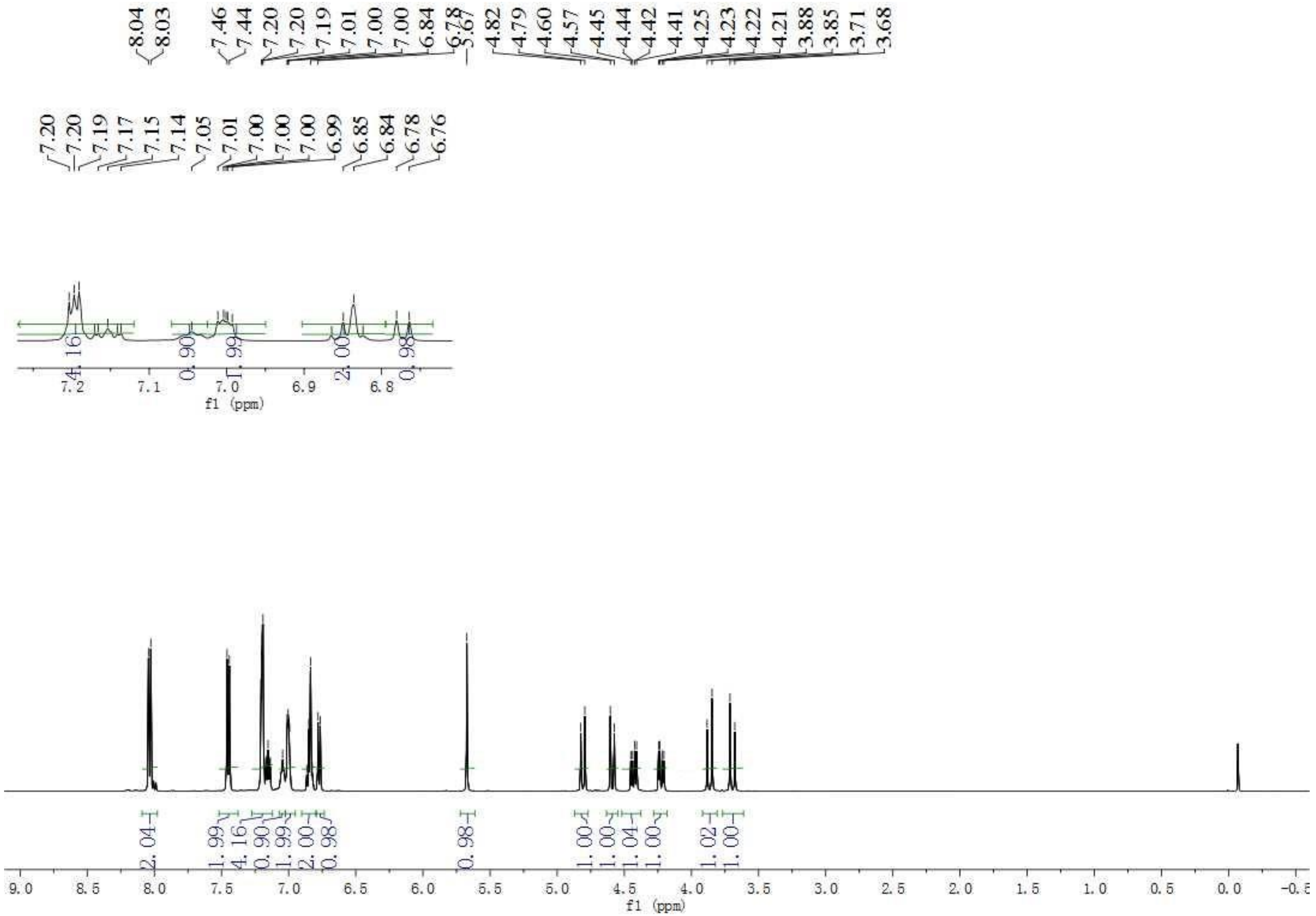

^{1}H NMR of compound **5r**

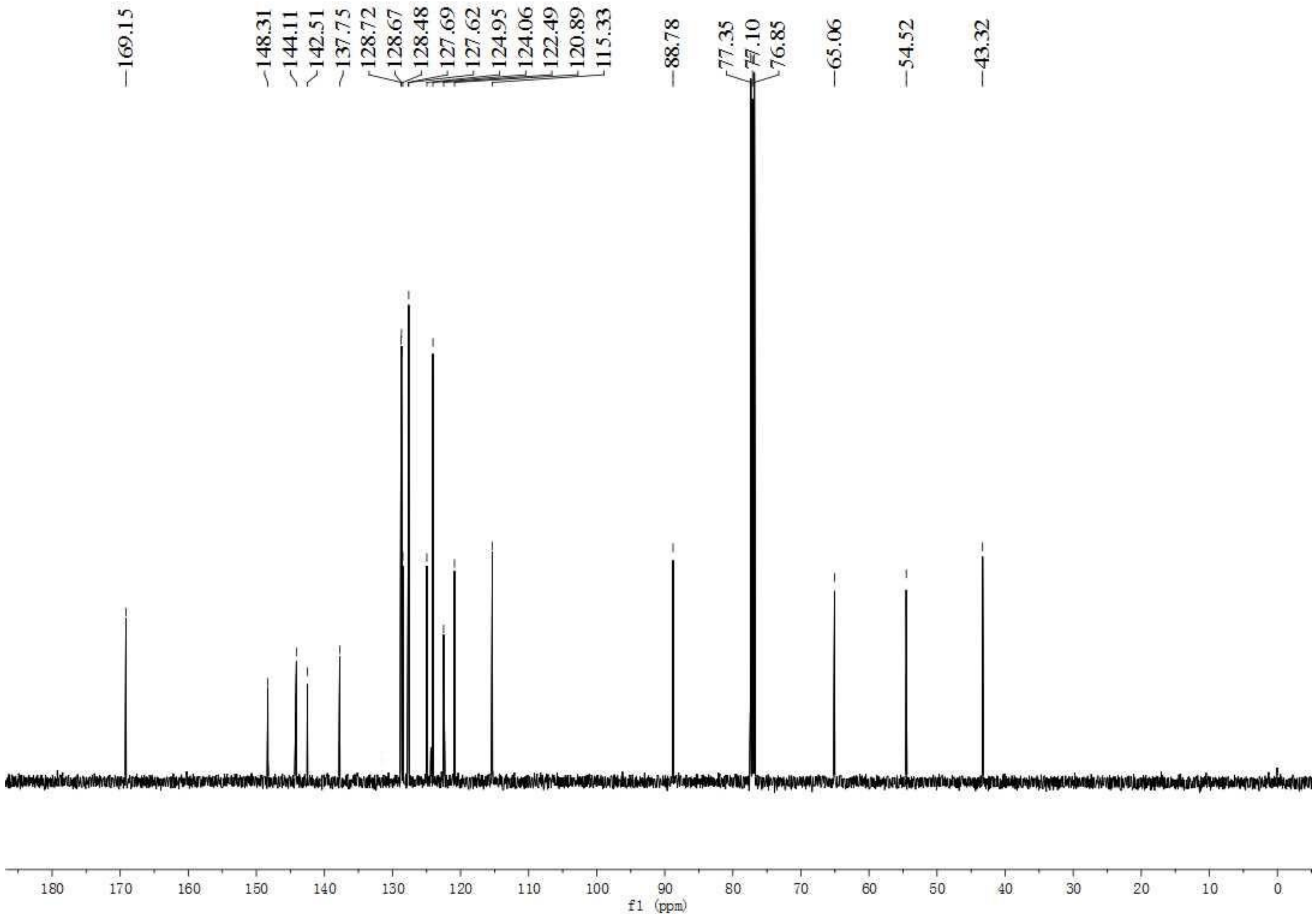

^{13}C NMR of compound **5r**
